# School-Based Nutrition Programs in the Eastern Mediterranean Region: A Systematic Review

**DOI:** 10.3390/ijerph20227047

**Published:** 2023-11-10

**Authors:** Ayoub Al-Jawaldeh, Dana Matbouli, Sarah Diab, Mandy Taktouk, Leila Hojeij, Sally Naalbandian, Lara Nasreddine

**Affiliations:** 1 Regional Office for the Eastern Mediterranean (EMRO), World Health Organization (WHO), Cairo 11371, Egypt; aljawaldeha@who.int; 2 Nutrition and Food Sciences Department, Faculty of Agriculture and Food Sciences, American University of Beirut, Beirut 1107 2020, Lebanon; dse12@mail.aub.edu (D.M.); smd39@mail.aub.edu (S.D.); mt86@aub.edu.lb (M.T.); lah36@mail.aub.edu (L.H.); 3 Science and Agriculture Library, American University of Beirut, Beirut 1107 2020, Lebanon; sn23@aub.edu.lb

**Keywords:** school, nutrition, children, adolescents, program, intervention, Eastern Mediterranean Region

## Abstract

This systematic review aims at documenting government-led school nutrition programs/interventions in countries of the Eastern Mediterranean Region (EMR). A systematic review of the available literature on this topic was conducted between 25 October 2022 and 15 November 2022 using 15 electronic databases as well as grey literature. The search was limited to materials published post 2000 in English, Arabic, or French. Articles/Reports were included in the review if they provided information on school-based nutrition programs/interventions developed, adopted or implemented by a governmental entity in the EMR countries, irrespective of study design. In total, 158 documents were retained until 16 May 2023. School-based programs/interventions were categorized into 13 types. In total, 298 school-based nutrition programs/interventions were identified. The most common were school meals and school feeding programs (all EMR countries) followed by nutrition education within the curriculum (77% of countries), extracurricular nutrition education (64%), standards for school canteens or foods/beverages available in schools (64%), and training of school staff (59%). Approximately half reported the inclusion of fruit and vegetable schemes (54%) or the establishment of hygienic cooking facilities (50%), while less than half reported standards for the marketing of food/beverages (45%), bans/standards for vending machines, milk feeding schemes, or micronutrient supplementation programs (41%). The least common interventions were school gardens (32%) and take-home rations (14%). Countries with the lowest gross domestic product and lowest government effectiveness score had the lowest number of programs/interventions. Many of the programs have tackled both school-aged children as well as preschoolers. We were able to identify monitoring and process evaluation for 21 programs in 14 countries. Few programs have undergone impact assessment.

## 1. Introduction

Child malnutrition is a serious public health issue since childhood and adolescence are important developmental phases in the lifespan when the roots for adult health and economic well-being are laid [1,2]. The Eastern Mediterranean Region (EMR) of the WHO, which encompasses 22 countries distributed over East/North Africa and South/West Asia [3], harbors a high burden of child malnutrition, in all its forms. 

The weighted regional average prevalence of stunting, wasting and underweight were estimated at 28%, 8.69%, and 18%, respectively [4], with wide variations in the prevalence of undernutrition between countries, given the significant inter-country disparities in income, economic development, political stability, and food security. In the region, Djibouti, Somalia, Sudan and Yemen have the highest prevalence of wasting, whereas Afghanistan and Yemen have the highest prevalence of underweight [4]. While undernutrition remains prevalent in many Eastern Mediterranean countries, the burden of overweight, obesity, and diet-related chronic disorders is increasing at an alarming rate [4]. The average weighted prevalence of overweight and obesity was estimated at 16.46% and 4.83%, respectively, amongst 13–15 year-old school-children in the EMR, with the highest burden being reported from high-income countries such as Kuwait (29.6%) [5], Bahrain (21.7%) [6] and the United Arab Emirates (UAE) (14.4%) [7].

To combat malnutrition in all of its forms, including undernutrition, micronutrient deficiencies, and obesity, a combination of evidence-based interventions across several domains is required to guarantee the availability and accessibility of healthy food [4]. In this context, schools represent an important setting to protect, promote, and support adequate nutrition in children, and thus constitute an opportunity to establish healthy dietary practices in childhood and beyond [8,9]. School-based nutrition interventions have the potential to address multiple components such as the establishment of standards for food, meals and beverages served within the school as well as other actions tackling the school environment, such as the bans or standards for vending machines and the regulation of food advertisement [8,10,11]. School nutrition programs also comprise curricula on nutrition and healthy diets, offer school health and nutrition services and collaborate with parents and the communities for the improvement of dietary practices amongst school children [8,10]. 

Available evidence suggests that school-based nutrition programs have favorable impact on the nutritional status and health of school-aged children [12,13]. It was for instance shown that school-based nutrition programs, including nutrition education, school meals, food environment modulation, and parental involvement, were effective in improving BMI levels amongst overweight and obese children, and reducing cardiometabolic risk factors [12,13]. School-based feeding, fortification and supplementation programs were also shown to be effective in reducing micronutrient deficiencies and improving the nutritional status of school-aged children [1]. Favorable cost–benefit ratios were also attributed to school-based nutrition programs [2]. An investment case model found that the cost–benefit ratio of school feeding programs range between 1:3 and 1:8, which means that for each dollar spent on school feeding, the government would receive at least three dollars in economic returns [2,14]. In a study on the economic gains associated with the introduction of school breakfast clubs, the benefits to costs ratio was estimated at 4.38 (the economic gain achieved over the life course amounted to £897,000, compared with £205,000 as the cost of the breakfast club) [15]. Available evidence also indicates that comprehensive, multicomponent school-based programs have a stronger long-term potential for generating and maintaining favorable health outcomes than single-component nutrition interventions [16]. 

Despite the high burden of malnutrition amongst school-aged children in the EMR, the Global Nutrition Policy Review 2016–2017 reported, that amongst all of the WHO regions, the region with the lowest rate of implementation of school-based nutrition programs was the EMR [8]. Acknowledging that school food and nutrition policies are implemented within complex systems that are essentially country specific, these policies are influenced by the country’s legal, political, economic, ethical, and cultural settings [2]. It is in this context that this systematic review is conducted with the aim of identifying and documenting existing government-led school nutrition programs and interventions in various countries of the EMR, and providing an overview of their impacts (when data is available). In addition, and given that the EMR region is characterized by a high diversity with respect to its countries’ social and income level [17,18]. This review also examines, in an exploratory analysis, the relation between the number of nutrition programs/interventions reported by the various countries and indicators pertinent to national economic resources (i.e., the Government Effectiveness Indicator (GEI) and the per capita GDP.

## 2. Materials and Methods

Data related to school-based nutrition interventions were obtained through a search of peer-reviewed and grey literature published up to 15 November 2022. The present systematic review conforms to the Preferred Reporting Items for Systematic Reviews and Meta-Analyses (PRISMA) statement (Refer to PRISMA Checklist in Supplementary Material) [19]. It has been registered in PROSPERO (CRD42023383783) after the completion of the databases’ search (21 January 2023) but before the initiation of screening, data extraction, and analysis. 

### 2.1. Search Strategy

A total of 16 electronic databases were searched between 25 October 2022 and 7 November 2022. These databases included: Academic Search Ultimate, CAB Direct, CINAHL Complete, Cochrane Library, Directory of Open Access Journals, Education Research Complete, Embase, Education Resources Information Center (ERIC), MEDLINE OVID, Scopus, Web of Science Core Collection, Al Manhal, Arab World Research Source (AWRS), E-Marefa and Iraqi Academic Scientific Journals (IASJ); the last four databases being specific to the Arab region. Google Scholar was also searched. In addition to using controlled vocabulary (MeSH in MEDLINE), a comprehensive list of search terms was used in the title/abstract/keyword fields to cover the four concepts (1) school, (2) nutrition, (3) intervention, and (4) EMR countries. The detailed list of search terms is shown in Appendix A (Table A1), an example of a database search is shown in Supplementary Table S1, and the detailed database search is shown in Supplementary Materials File S1. The search of these electronic databases was conducted by one author only (SN). Newly published articles after the execution of the initial search were identified through email alerts (up until 16 May 2023). 

A search of the grey literature was also conducted by another author (MT), using Google, the Global Database on the Implementation of Nutrition Action (GINA), the WHO EMRO (Regional Office for the Eastern Mediterranean) website and governmental websites (e.g., Ministries of Health). 

### 2.2. Inclusion and Exclusion Criteria

- Articles/Reports were included in this review if they provided information relevant to school-based (or preschool-based) nutrition programs or interventions that have been developed, adopted or implemented by a governmental entity in countries of the EMR, irrespective of study design.
- Articles reporting on school interventions that did not involve a governmental entity or governmental oversight were not included in the review.
- Interventions targeting specific populations (individuals on therapeutic diets etc.) were excluded.
- Individual articles/reports were excluded if they were published prior to 2000, or if they were in languages other than English, Arabic, and French.
- There were no exclusion criteria based on the design of the study.

### 2.3. Study Selection

Articles identified through the search of online databases and grey literature were exported to EndNote X9.3.3. In Endnote, duplicates were identified and removed. Duplicates were identified and removed. This was followed by the first screening stage which consisted of screening for title and abstract. The second stage of screening consisted of going over the full text of the identified documents to assess eligibility and to potentially identify additional sources from within the references. 

Two independent researchers (SD and LH) screened the titles, abstracts and full text articles of the potentially relevant articles/reports, based on the inclusion and exclusion criteria described in the section above. The two researchers discussed and resolved the minor discrepancies that resulted from the two screening stages. Conflicts at the level of article selection were resolved by another author (LN).

### 2.4. Data Extraction

For each school-based nutrition program/intervention, the key characteristics were extracted and entered into a database constructed by the researchers. For this purpose, data extraction tables were created, before the search. The development of these tables was guided by the WHO global nutrition policy review 2016–2017 [8] and the classification of interventions adopted within this global review. Accordingly, school-based interventions were categorized as follows: (1) nutrition education included in school curriculum; (2) extracurricular nutrition education; (3) training of school staff (teachers, canteen staff etc.); (4) standards or rules for foods and beverages available in schools; (5) bans or standards for vending machines in schools; 6. standards for marketing of food and nonalcoholic beverage; (7) provision of school meals, school feeding program; (8) take-home rations distributed through schools; (9) school fruit and vegetable scheme; (10) school milk scheme; (11) micronutrient supplementation; (12) hygienic cooking facilities and clean eating environment in schools; and (13) school gardens. 

Therefore, thirteen different extraction tables were prepared, focusing on the type of school-based nutrition program/intervention, its scope of implementation (national or regional), leadership, year of implementation and duration, target population (school children within the age of 6–18 years or preschool children within the age of 3–5 years), objectives and a brief description of the policy/intervention, as well information in its monitoring, evaluation or impact assessment. Data extraction was conducted independently by two researchers (SD and DM), and then a third researcher (LN) reviewed the data for accuracy. Any inconsistency was resolved through discussion until reaching consensus. 

### 2.5. Analysis

Analysis was carried out between 15 June and 15 July 2023. Information on the identified school-based nutrition programs and their core characteristics were entered into the database, according to the pre-developed extraction framework that included the types of school-based programs/interventions listed above and their core characteristics. A quantitative assessment was performed to (1) determine the number and proportion of countries implementing the different types of nutrition programs and (2) identify the most commonly implemented types of programs in the region. An appraisal of the program’s objectives and impact was conducted when applicable. 

The number of programs/interventions implemented by the various countries were examined in function of the countries’ most recent GEI (2022) [20]. The GEI is one of the indicators developed by the Worldwide Governance Indicators project, and reflects the “perceptions of the quality of public services, the quality of the civil service and the degree of its independence from political pressures, the quality of policy formulation and implementation, and the credibility of the government’s commitment to such policies” [21]. It is expressed as a percentile, ranging from 0 (lowest) to 100 (highest) [22]. Data on this indicator were retrieved from the World Bank—Worldwide Governance Indicators website [22]. The number of school-based nutrition programs/interventions was also examined in light of the country’s per capita GDP [23], which “provides a basic measure of the value of output per person, ….an indirect indicator of per capita income” [24]. 

## 3. Results

### 3.1. Search Results

**Figure 1:**
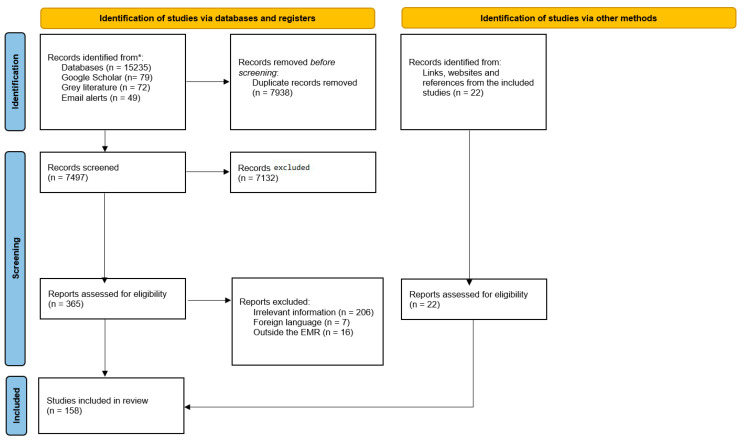
Search and identification process of potential references from the literature. * The records identified from the various databases were as follows: Academic Search Ultimate (n = 1162); Al Manhal (n = 23); Arab World Research Source (AWRS) (n = 412); CAB Direct (n = 1879); CINAHL Complete (n = 1186); Cochrane (n = 636); Directory of Open Access Journals (n = 21); Education Research Complete (n =117); E-Marefa (n = 23) Embase (n = 2043); ERIC (n=131); Iraqi Academic Scientific Journal (IASJ) (n = 12); MEDLINE (n= 1476); Scopus (n = 4195); Web of Science (n =1919). Adapted with permission from Ref. [19], http://www.prisma-statement.org/ (accessed on 2 October 2023).

Figure 1 shows the search and identification process of potential references from the literature. In total, 15,435 documents were initially obtained after searching the databases and grey literature, after which duplicates (n = 7938) were removed. Therefore, a total of 7497 were screened in the first stage, characterized by screening for title and abstract. Accordingly, 7132 documents were excluded. The second stage of screening, which included going over the full text (365 documents), led to the exclusion of 229 documents. In addition, 22 sources were identified from links, webpages and references from within the included studies. Thus, a total of 158 documents were retained in this review.

When examining the number of studies/reports identified per country, the largest number of articles/documents was obtained from Iran (23) [8,25,26,27,28,29,30,31,32,33,34,35,36,37,38,39,40,41,42,43,44,45,46] and the UAE (22) [26,34,37,47,48,49,50,51,52,53,54,55,56,57,58,59,60,61,62,63,64,65]; followed by Bahrain (13) [8,26,37,40,44,48,50,51,55,66,67,68,69], KSA (11) [8,26,37,39,48,50,51,70,71,72,73], Jordan (10) [8,26,39,43,50,51,74,75,76,77], Lebanon (10) [8,26,37,39,50,51,78,79,80,81], Kuwait (9) [8,26,37,39,44,48,82,83,84], Egypt (8) [8,37,43,85,86,87,88,89], Morocco (8) [8,43,90,91,92,93,94,95], Qatar (8) [8,37,39,48,96,97,98,99], Palestine (7) [26,39,50,51,100,101,102], Oman (6) [8,37,39,48,55,103] and Tunisia (6) [8,43,44,104,105,106]. The countries with the lowest number of studies/reports were Pakistan (5) [40,107,108,109,110], Afghanistan (4) [111,112,113,114], Iraq (4) [8,44,115,116], Somalia (2) [117,118], Sudan (2) [119,120], and Yemen (1) [8]. 

### 3.2. Types of School-Based Programs/Interventions 

Table 1 displays in a color-coded format, the types of school-based programs/interventions implemented in each country of the EMR. The most common school nutrition programs implemented in the EMR were: provision of school meals and school feeding programs (all EMR countries) followed by inclusion of nutrition education in the school curriculum (17 countries; 77%), extracurricular nutrition education (14 countries; 64%), establishment of standards pertinent to school canteens or foods and beverages available in schools (14 countries; 64%) and training of school staff (13 countries; 59%).

Approximately half of the countries have reported the inclusion of fruit and vegetable schemes within the school setting (12 countries; 54%) and the establishment of hygienic cooking facilities and clean eating environments (11 countries; 50%). Less than half of the EMR countries have developed standards for the marketing of food and beverages within the school setting (10 countries; 45%), or reported bans or standards for vending machines, milk feeding schemes or micronutrient supplementation programs (9 countries; 41%). The least common type of programs was the school gardens which was reported by only 7 countries of the region (32%) and the take-home rations (3 countries; 14%). 

### 3.3. School-Based Nutrition Programs in Relation to Government Effectiveness and GDP

**Figure 2:**
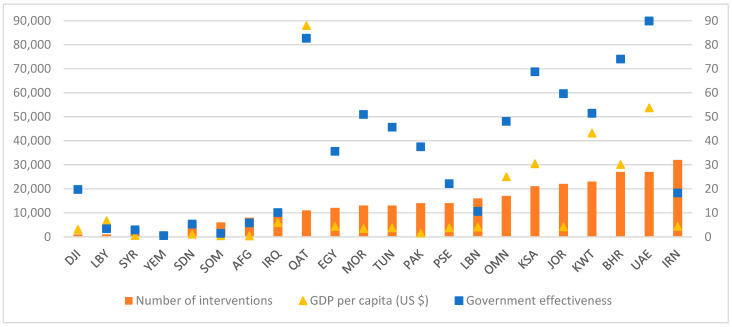
Number of school-based nutrition programs reported from countries of the EMR, in relation to the countries’ government effectiveness rank and per capita GDP. Abbreviations: EMR: Eastern Mediterranean Region; GDP: gross domestic products; YEM: Yemen; SOM: Somalia; SYR: Syria; LBY: Libya; SDN: Sudan; AFG: Afghanistan; IRQ: Iraq; LBN: Lebanon; IRN: Iran; DJI: Djibouti; PSE: Palestine; EGY: Egypt; PAK: Pakistan; TUN: Tunisia; OMN: Oman; MOR: Morocco; KWT: Kuwait; JOR: Jordan; KSA: Kingdom of Saudi Arabia; BHR: Bahrain; QAT: Qatar; UAE: United Arab Emirates.

Data presented in Figure 2 shows that in general, as government effectiveness and GDP increase, the number of government-led school-based nutrition programs increases. In fact countries with the lowest GDP and the lowest government effectiveness rank had the lowest number of school-based interventions (Afghanistan, Djibouti, Libya, Somalia, Sudan, Syria and Yemen). Some exceptions can be noted such as Qatar, which has the highest GDP in the region, coupled with a high government effectiveness rank, but had a rather limited number of interventions. Other exceptions are countries such as Jordan which, despite having a relatively low GDP, had a high government effectiveness index coupled with a high number of school-based interventions.

The countries that have the lowest number of school-based nutrition programs included Djibouti (1 program), Libya (1 program), Syria (1 program), Yemen (3 programs) and Somalia (3 programs). Out of the 12 different categories of school-based nutrition interventions, 6 types or less were implemented in Afghanistan, Djibouti, Libya, Somalia, Sudan, Syria and Yemen. The highest number of school-based interventions were implemented in Iran (11 types of interventions, 32 programs in total), Bahrain (11 types of interventions, 27 programs in total), Kuwait (10 types of interventions, 23 programs in total), Jordan (9 types of interventions, 22 programs in total), KSA (9 types of interventions, 21 programs in total), Oman (10 types of interventions, 17 programs in total), Lebanon (8 types of interventions, 16 programs in total), and UAE (7 types of interventions, 27 programs in total). 

### 3.4. School-Feeding Programs

**Figure 3:**
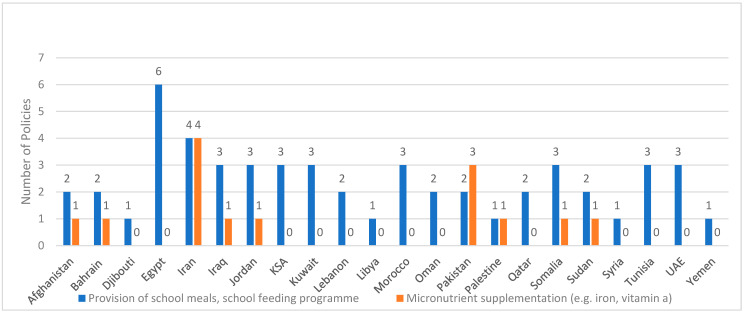
Number of identified school feeding and micronutrient supplementation programs in various countries of the EMR. References on provision of school meals and school feeding programs: Afghanistan: [111,121]; Bahrain: [37,44,48,121]; Djibouti: [121]; Egypt: [8,37,43,85,86,87,88,121]; Iran: [8,25,26,27,121]; Iraq: [8,44,121]; Jordan: [8,43,121]; KSA: [8,48,121]; Kuwait: [8,48,121]; Lebanon: [78,121]; Libya: [121]; Morocco: [8,43,90,91,121]; Oman: [8,44,48,55,103]; Pakistan: [107,108,121]; Palestine: [121]; Qatar: [48,121]; Somalia: [117,118,121]; Sudan: [119,120,121]; Syria: [121]; Tunisia: [8,43,44,121]; UAE: [47,48,61,65,121]; Yemen: [121]. References on micronutrient supplementation: Afghanistan: [111]; Bahrain: [44]; Iran: [8,28,29,30,44]; Iraq: [44]; Jordan: [75]; Pakistan: [40,109,110]; Palestine: [100,101]; Somalia: [117]; Sudan: [119,120]. Abbreviations: EMR: Eastern Mediterranean Region; KSA: Kingdom of Saudi Arabia; UAE: United Arab Emirates.

As shown in Figure 3 and Supplementary Table S2, there is evidence of implementation of school feeding programs in all countries of the region, although information on coverage was not clearly provided for all countries. Based on the school feeding programs implemented in coordination with the World Food Programme (WFP), the estimated coverage varied between 6% in Lebanon and 73% in Egypt in 2022 [121] (Table 2). In countries with low GDP (Afghanistan, Lebanon, Morocco and Sudan), the majority of school feeding programs were supported by external entities, such as the WFP. In addition to the programs listed in Figure 3, there was evidence of implementation of take-home rations distributed through schools in Egypt, Iraq, and Yemen [8,44]. The majority of the countries have also included preschool children enrolled in kindergartens in addition to children enrolled in schools (Supplementary Table S2).

The objectives of the school feeding programs reported from EMR countries are shown in Supplementary Table S2. These objectives can be categorized into four broad categories: reduction or prevention of undernutrition, micronutrient deficiencies, food insecurity or hunger; reduction or prevention of overweight and obesity; enhancing access to education, school enrollment, attendance and performance; promoting healthy dietary practices and improving knowledge about healthy meals and diets. Programs implemented in Qatar and UAE had more focus on obesity prevention while those implemented in Afghanistan, Lebanon, Pakistan, Somalia, and Sudan were more focused on prevention of undernutrition and improving food security. 

### 3.5. Micronutrient Supplementation Programs

As shown in Figure 3 (and Supplementary Table S2), micronutrient supplementation programs in the school setting are reported from nine countries of the region (41%). Different implementation schemes have been reported from the various countries. For instance, the provision of foods/school meals fortified with micronutrient powders (MNPs) was reported from Afghanistan [111] while the provision of biscuits fortified with vitamins A, B1, B2, B3, B6, B12 as part of the school lunch was reported from Jordan [75]. Other countries have reported the distribution of micronutrient supplements (e.g., iron, folic acid, vitamin D, vitamin A, or multiple micronutrients) in the school setting, including Iran, Iraq, and Pakistan [8,28,29,30,40,44,109,110]. Countries such as Bahrain, Iran, Iraq and Jordan have also included kindergartens in their micronutrient supplementation programs (Supplementary Table S2). 

The objectives of the various micronutrient supplementation programs have essentially focused on increasing the intake of certain micronutrients amongst children/adolescents, reducing the prevalence of micronutrient deficiencies and undernutrition such as stunting, as well as improving cognitive performance and school attendance (Supplementary Table S2). The prevention of NCDs has been also highlighted as an objective of some of these micronutrient supplementation programs. For instance, the vitamin D supplementation program in Iran had, as an objective, the reduction of cardiovascular disease risk in adulthood through improving adolescents’ serum levels of vitamin D [28,29]. The objectives of the program reported from Jordan also included the reduction of diet-related diseases and NCDs [75].

### 3.6. Inclusion of Nutrition Education in School Curricula 

**Figure 4:**
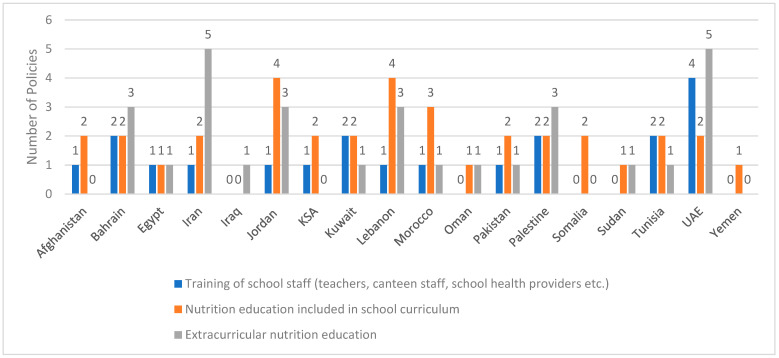
Number of identified school-based programs that include curricular nutrition education, training of school staff or extracurricular nutrition education in various countries of the EMR. References on training of school staff: Afghanistan: [113]; Bahrain: [50,55]; Egypt: [89]; Iran: [27]; Jordan: [50]; KSA: [50]; Kuwait: [8,26,82]; Lebanon: [50]; Morocco: [94,95]; Pakistan: [107]; Palestine: [50,100,101]; Tunisia: [104,105,106]; UAE: [49,50,57,58]. References on nutrition education in school curriculum: Afghanistan: [112,113]; Bahrain: [8,26,50,51]; Egypt: [8]; Iran: [8,27,31,33]; Jordan: [8,26,50,51,74,75]; KSA: [8,26,50,51]; Kuwait: [8,26,82]; Lebanon: [8,26,50,51,79,80]; Morocco: [8,92,93,94,95]; Oman: [8]; Pakistan: [109,110]; Palestine: [26,50,51,100,101]; Somalia: [117,118]; Sudan: [119,120]; Tunisia: [8,104]; UAE: [26,49,50,51]; Yemen: [8]. References on extracurricular nutrition education: Bahrain: [55,66,67]; Egypt: [85]; Iran: [8,27,31,32,33,34,35,36]; Iraq: [115]; Jordan: [8,26,50,51,75,76,81,83,84]; Morocco: [8]; Oman: [55,103]; Pakistan: [110]; Palestine: [50,100,101,102]; Sudan: [119,120]; Tunisia: [105,106]; UAE: [26,34,52,53,54,55,56,63,64]. Abbreviations: EMR: Eastern Mediterranean Region; KSA: Kingdom of Saudi Arabia; UAE: United Arab Emirates.

Seventeen countries have reported programs that comprise nutrition education in school curricula (Figure 4; Supplementary Table S3). The topics covered within these nutrition education programs included lessons on healthy diet, the links between nutrition and health, healthy eating practices for the prevention of overweight and obesity, as well as healthy cooking practices. Many of these programs have also covered kindergartens in addition to schools. The objectives of many of these programs focused on improving children’s knowledge and fostering healthy diet and lifestyle habits. In other instances, the programs had broader objectives such as “to reduce nutrition related mortality and morbidity and contribute to economic development of the nation through reduction in all forms of malnutrition particularly stunting, micronutrients deficiency and acute malnutrition” in Afghanistan or “to focus on remedial measures for addressing nutritional issues that have not only been adversely affecting the behavioral, cognitive, scholastic, physical performances but have also been increasing morbidity and mortality and impairing socioeconomic development” in Pakistan.

### 3.7. Inclusion of Extracurricular Nutrition Education

Extracurricular nutrition education initiatives were reported from 14 countries (Figure 4; Supplementary Table S4). Some of these programs were in the form of extracurricular lectures given in the school setting, the provision of written educational materials, the organization of healthy cooking activities or class activities that promote awareness around nutrition and health. Other extracurricular programs extended beyond the school, targeting parents, family or even communities. For instance, in Iran, the Weight and Obesity Control in Students (Kouch) program aimed to promote healthy food environment not only in the school setting but also at home, through nutrition education of students as well as their parents [27]. After the COVID-19 pandemic, these educational activities have been continued virtually through the Shad application designed by the Iranian Ministry of Education (MOE) [27]. In Palestine, and through the Nutrition Friendly School Initiative, extracurricular initiatives included the development of educational and communication material for community awareness [100,101]. 

### 3.8. Training of School Staff

Programs that comprise the training of school staff on issues pertinent to food and nutrition were reported from 13 EMR countries (Figure 4; Supplementary Table S5). Some of these programs were specifically focused on capacity-building and raising nutrition awareness amongst school and/or kindergarten teachers, staff and administrators. For instance, in the UAE, the new school canteen rules for Abu Dhabi included a training manual for administrators, nutritionists, nurses and supervisors of school canteens in schools [57]. Other implemented trainings were part of broader programs such as the Health-Promoting Schools program in Bahrain [55], the National School Screening and Management Programme in Egypt [89], the Kuwait National Programme for Healthy Living [26,82] and the National Multisectoral Strategy for the Prevention and Control of Non-Communicable Diseases (NCD) in Tunisia [105,106]. 

### 3.9. Standards Pertinent to School Canteens, Marketing of Food/Beverages or Vending Machines

**Figure 5:**
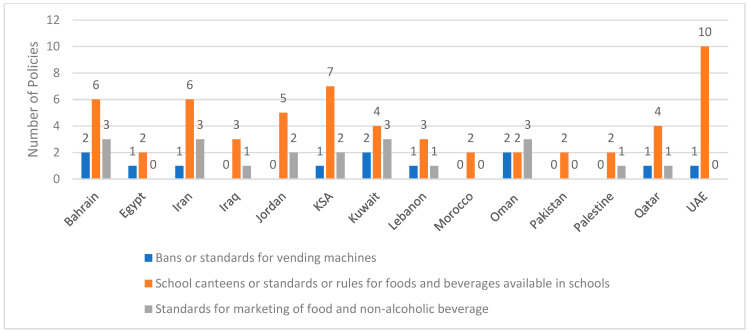
Number of identified school-based programs that include standards pertinent to school canteens, marketing of food/beverages or vending machines in various countries of the EMR. References on bans or standards for vending machines: Bahrain: [8,44]; Egypt: [8]; Iran: [8]; KSA: [8]; Kuwait: [8,44]; Lebanon: [8]; Oman: [8,44]; Qatar: [8]; UAE: [59]. References on school canteens of standards or rules for foods and beverages available in schools: Bahrain: [8,26,37,50,55,66,69,76,122]; Egypt: [8,85]; Iran: [8,27,31,32,37,38,39,40,41,42,43,45]; Iraq: [8,115,116,123]; Jordan: [8,26,39,50,75,77,122]; KSA: [8,37,50,70,71,72,73,124,125]; Kuwait: [8,26,37,82,83,84]; Lebanon: [8,26,37,50,124]; Morocco: [8,94,95]; Oman: [8,55,103]; Pakistan: [40,110]; Palestine: [26,50,102,124]; Qatar: [8,37,96,97]; UAE: [26,37,47,50,55,57,59,60,61,62,63,64,65,124]. References on standards for marketing of food and non-alcoholic beverage: Bahrain: [8,37,40,44]; Iran: [27,39,44]; Iraq: [116,123]; Jordan: [8,39]; KSA: [8,39]; Kuwait: [8,39,44]; Lebanon: [39]; Oman: [8,39,44]; Palestine: [39]; Qatar: [39]. Abbreviations: EMR: Eastern Mediterranean Region; KSA: Kingdom of Saudi Arabia; UAE: United Arab Emirates.

As shown in Figure 5, 13 countries have reported the establishment of standards for school canteens or for foods and beverages available in schools and kindergartens. The highest number of reported programs were from GCC countries, namely the Bahrain, KSA and the UAE, as well as Iran. In addition, some countries have reported on standards related to food vendors and outlets around the schools. For instance, the IRAN-Ending Childhood Obesity program aimed at limiting the availability of unhealthy snacks and fast foods by street food vendors around the schools [27,32]. Similarly, in Egypt, the 100 Million Health Initiative included the provision of outlets for selling healthy and safe food in the vicinity of schools and public places [85].

In addition, nine countries have reported bans or standards for vending machines in the school setting (Figure 5) (more details on the various programs are given in Supplementary Table S6). Complete bans on vending machines in schools and kindergartens were reported from Egypt, KSA and Lebanon [8], while other countries have reported the adoption of standards on foods/beverages sold in vending machines, including Iran [8], Qatar [8] and the UAE [59]. Although bans on vending machines were described in earlier reports from Bahrain [44], Kuwait [44] and Oman [44], recent reports indicate that these countries have reported the adoption of standards on foods/beverages sold in vending machines instead of bans [8]. 

Ten countries have reported on the establishment of standards for the marketing of foods and beverages in schools (Figure 5). Accordingly, the marketing of high-fat, high-energy and micronutrient-poor foods and beverages is prohibited in the school premises in these countries, based on pre-specified lists and standards. These foods/beverages include, amongst others, soft drinks, potato crisps and sweet biscuits. In addition, a clear reference to energy drinks is included in the marketing bans in Bahrain. Except for Palestine and Qatar, reports from other countries indicate that kindergartens are also covered by these standards. 

The objectives of the various programs that adopt standards for school canteens/foods offered in schools, vending machines or marketing of food/beverages are all shown in Supplementary Table S6. Some of these objectives are very specific such as “enhancing the food prepared in school canteens, quantity and quality wise” in Bahrain [69], or “improving healthy nutrition of school students to treat and prevent diseases resulting from malnutrition” in Egypt [85], and “increase access to healthy snacks; and prevent the supply of foods with low nutritional value” in Iran [27,39,40,41,42,45]. In other instances, the objectives are broader since these standards are part of wider programs implemented in the country. For example, in the UAE, the objectives were to “improve the nutritional status of all population in the UAE with a collective vision of a healthier and sustainable future; guided by the international, regional, and national policies and strategies to promote health” [59].

### 3.10. Fruit, Vegetable and Milk Schemes

**Figure 6:**
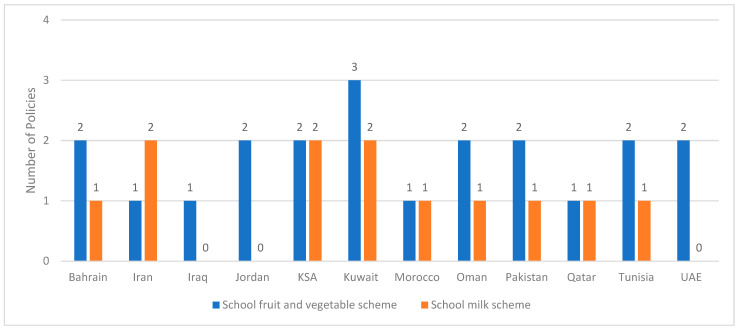
Number of identified school-based programs that include fruit/vegetables or milk schemes in various countries of the EMR. References on school fruit and vegetable scheme: Bahrain: [44,67]; Iran: [27,42]; Iraq: [8]; Jordan: [8,26]; KSA: [70,71,72]; Kuwait: [8,37,44]; Morocco: [8]; Oman: [8,44,55,103]; Pakistan: [40,110]; Qatar: [37]; Tunisia: [44,105,106]; UAE: [47,59,61,65]. References on school milk scheme: Bahrain: [44]; Iran: [8,27]; KSA: [8,70]; Kuwait: [37,44]; Morocco: [8]; Oman: [8,44]; Pakistan: [110]; Qatar: [37]; Tunisia: [44]. Abbreviations: EMR: Eastern Mediterranean Region; KSA: Kingdom of Saudi Arabia; UAE: United Arab Emirates.

As shown in Figure 6, twelve countries had reported fruit and vegetable schemes, while nine had reported milk schemes in the school or kindergarten setting. Various approaches were adopted within these schemes, including improving the visibility and attractiveness of fruits and vegetables in school cafeterias/shops/canteens; including fruits and vegetables in the students’ daily menu, offering fruit and vegetables as part of breakfast [8,26,27,37,42]. Milk schemes were also implemented via the direct provision of milk to students, or via the inclusion of milk as part of the healthy options in the school cafeteria/canteen/shops (Supplementary Table S7).

### 3.11. Hygienic Cooking Facilities and Clean Environments in Schools

There were reports from 11 countries on such interventions, the highest number being reported from Bahrain (3), Iran (3) and Oman (2), followed by Afghanistan, Iraq, KSA, Kuwait, Lebanon, Palestine, Qatar and Tunisia (one in each). The programs implemented in Afghanistan, Palestine, and Sudan were reported to cover schools, while the programs scope was extended to kindergartens in the other countries (Supplementary Table S8). 

### 3.12. School Gardens

School gardens were the least implemented type of intervention in countries of the region, with only seven countries reporting such interventions (Supplementary Table S8). The scope of these interventions covered schools in Afghanistan, Palestine, and Sudan [100,101,114,119,120], while it extended to both schools and kindergartens in Jordan, Morocco, Oman and Tunisia [8]. Although some of these programs were reported to be implemented two decades ago (in 2002 in Afghanistan, 1999 in Jordan and 1996 in Oman), there is no information on whether these were continued/sustained. 

### 3.13. Monitoring and Process Evaluation

We were able to identify monitoring and process evaluation for 21 programs in 14 countries of the region, including Afghanistan, Bahrain, Iran, Iraq, Jordan, KSA, Kuwait, Lebanon, Morocco, Oman, Pakistan, Palestine, Qatar and the UAE (Supplementary Table S9). Some of these programs had a clear set of evaluation indicators such as the meal fortification program (number of children receiving MNP fortified meals; number of days MNP was used) and the school garden project (number of schools with school gardens available; number of children participating in school gardening recreational activities) in Afghanistan [111,114]. Other programs with established evaluation indicators included the RASHAKA Initiative in KSA (e.g., number of education campaigns conducted in a year; number of schools that carry out the initiative; number of training sessions per month given to primary healthcare workers and teachers) [71,72], the National Action Plan in Nutrition 2017–2021 in the UAE (e.g., number of schools that have made fruits and vegetables available to students; number of schools that have incorporated and implemented nutrition education and activities in the school curriculum) [59], the National Nutrition and Physical Activity Action Plan 2011–2016 (e.g., number of schools participating in the national school snack program) and the Food Safety and Health Guide (e.g., breakfast and food meals provided in schools or sent from home) in Qatar [98,99]. 

Other programs had described process evaluation measures such as internal and external auditing (e.g., Health-Promoting Schools program in Bahrain [55]; the Iranian health promoting schools (IHPSs) program and the Healthy schools canteen program in Iran [31,33]). Supplementary Table S9 provides more details on the various evaluation activities conducted within the identified programs. 

### 3.14. Impact Assessment

Few programs in the region have undergone impact assessment. In Iran, Sayyari et al. [32] investigated the impact of a one-year school-based intervention conducted within the Iran-ending childhood obesity (Iran-ECHO) program. A quasi-experimental study was conducted in six provinces of Iran to assess nutritional knowledge amongst 7149 students aged 7–18 years, from urban areas in six provinces of Iran. Findings showed that, although a high proportion of students had acceptable knowledge about the adverse health effects of overweight/obesity, their knowledge about the low nutritional value of unhealthy snacks such as puffs, potato chips, carbonated drinks, and industrial juices, remained suboptimal. A non-negligible proportion of students reported the undesirable practice of skipping one of the main meals for weight loss purposes [32]. Another study in Iran was conducted with the aim of assessing the impact of the Isfahan Healthy Heart Program-Heart Health Promotion from Childhood, which aims to improve knowledge, and behavior towards healthy nutrition and decrease the prevalence of obesity and other cardiovascular risk factors [34,35,36]. The evaluation of this program showed an increase in nutrition knowledge, and a reduction in mean waist circumference and the levels of total and low-density lipoprotein-cholesterol amongst children in the intervention community. Another study in Iran examined the effectiveness of the National iron supplementation program, which targeted adolescent girls attending public or private senior high schools in rural and urban areas [46]. Compliance (defined as the full intake of the received tablets during the 16 weeks of the intervention) was estimated at 62%, while refusal to take the tablets and intermittent compliance were reported at 27%, and 11%, respectively. From the perspective of students, factors that decreased compliance (and hence the potential impact of the intervention) included the absence of a direct classroom water supply, abdominal discomfort, unpleasant taste of the pills, and a feeling of dizziness. From the perspectives of school principals and teachers, the low quality of tablets, absence of classroom water supply, parent illiteracy, as well as poor collaboration on behalf of both the school teachers and students were the key factors affecting compliance rates. 

In Jordan, Khatib and Hijazi [126] evaluated the impact of the School Snack Service (SSS) program led by MOE, in a cross-sectional study of 468 school children (66–120 months), who were recipients of SSS, from 43 villages, affiliated to eight disadvantaged areas of Jordan. Blood samples and anthropometric measurements were obtained. The study documented a persistent burden of micronutrient deficiencies whereby the prevalence of anemia was estimated at 19.9%, and that of subclinical vitamin A deficiency at 32.9%. The proportion of children at risk of becoming vitamin A deficient was as high as 58.3%. 

In KSA, Aldubayan and Murimi 2019 [127] assessed school compliance with the Saudi policy chapter “Meals and beverages offered in school canteens”. For this purpose, 76 public high schools were selected from four areas in Riyadh, and the nutritional compositions of food offered in these schools were assessed. Although the selected schools had moderate compliance with the policy and all of the schools banned soft drinks and energy drinks, 96.1% of these schools had low alignment with the policy when comparing the offered foods to the nutritional standards. The study concluded that, while the Saudi policy is clear on what foods should not be served in school canteens, it fails to offer guidance on what foods should be served to enhance the meals’ nutritional value. Another study conducted in KSA by Gordon et al. [128] aimed to assess the nutritional content of breakfast meals in all-girl intermediate and secondary public schools in Mecca, and whether these meals are aligned with the School Healthcare Department guidelines. The results showed that the foods served had a low content of iron, calcium and vitamin D, coupled with a high content of sugar and sodium. The refusal and reluctance of students to purchase healthy food options were the main barriers against serving healthy foods in the school. In addition, the majority of foodservice managers did not have a nutrition background, although they were responsible for food ordering and planning food lists for the schools.

In Lebanon, a cross-sectional survey was conducted to compare between the 10 Health Promoting School (HPS) networks and 10 control public/schools from the same geographic regions (n = 2105 students aged 10–15 years; grades 6–9) [80]. More specifically, the study aimed at exploring school health services, students’ dietary practices and the extent to which the HPS was contributing to adolescents’ well-being. Data collection was based on the Youth Risk Behavior Survey as well as anthropometric measurements. Findings showed that the school health program failed to improve health behaviors amongst adolescents, including dietary practices. Evidence gathered from this research highlighted poor investment at all levels for implementation, sustainability and evaluation. Another study conducted in Lebanon, examined the impact of the Ajyal Salima program (formerly known as Health-E-PALS), which aims to promote healthy eating and active lifestyles amongst school children aged 9–11 years [26]. The study showed that nutritional knowledge and self-efficacy had significantly increased amongst students in intervention group as well as the odds of consuming breakfast. It also showed a reduction in students’ intake and purchase of salty energy-dense snacks. In contrast, the intervention did not have a significant effect on fruit consumption, and this was explained by the fact that the program failed in changing the schools’ food environment and in increasing the availability of fresh fruit in the school shops. 

In Tunisia, Ghammam et al. [129] conducted a quasi experimental study to assess the long term effect of the “Together in Health” school-based program, which aims to reduce NCD risk factors. The study was conducted amongst school children, aged 11 to 16 years. In order to assess the program’s effectiveness, dietary knowledge and attitudes of students in the intervention and control groups were evaluated at baseline, at the end of the three-year program period and at one year after the program’s cessation (follow-up). Anthropometric measurements were also obtained. The study findings showed that the proportion of students who reported the consumption of 5 fruits and vegetables per day had increased significantly in the intervention group (even one year after the program’s cessation), while a significant decrease was observed in the control group. The study also showed a significant decrease on the prevalence of overweight in the intervention group, while an increase was noted in the control group. The study concluded that the majority of the program’s outcomes showed significant improvement, at least in the short term, which supports the need for continued and sustained action.

In the UAE, the impact of the “Eat Right Get Active” program was investigated [58], in a pilot study conducted amongst 25 public schools. The study reported an increase by 60% in the proportion of students consuming servings or more of fruits per week. In addition, the findings highlighted an increase in the availability of nutrition policies and of educational tools fostering healthy eating.

## 4. Discussion

This systematic review is the first to identify and characterize government-led school nutrition programs in various countries of the EMR, a region that harbors a double burden of malnutrition amongst children and adolescents [4,130,131]. It showed that the most commonly implemented type of school nutrition programs was the provision of school meals/school feeding programs (all EMR countries; 100%) while the least implemented type were school gardens and take-home rations. The global analysis of school-based nutrition programs that was featured in the WHO Global Nutrition report of 2016–2017 provided a different picture, with nutrition education in the school curriculum being the most common type of nutrition program implemented worldwide (61% of countries worldwide), while school meals/school feeding programs were reported by only 54% of countries [8].

Apart from few exceptions, the study findings suggest a link between government effectiveness, national income (GDP), and the number of government-led school-based nutrition programs in countries of the region. The lowest number of interventions was in fact observed in countries that had the lowest GDP as well as the lowest government effectiveness score (Djibouti, Libya, Syria, Yemen, Sudan, Somalia and Afghanistan). Previous studies that have examined the implementation of health policies in relation to national economic and political indicators have also reported similar observations [132]. For instance, Mackenbach and Mckee, 2013 [133], showed that national income and government effectiveness in European countries were amongst the main predictors of countries’ performance in several areas of health policy, including fruit and vegetable consumption and alcohol-related policies. Expectedly, richer countries (with higher GDP) would, on average, have more resources to implement a higher number of health policies compared to poorer countries. In addition, countries with better scores on government effectiveness, as determined by, for instance, the functioning of governmental departments and agencies, the professionalism of the civil service and the absence of corruption, are also expected to have implemented more health policies [134]. To further compound the issue, the countries that, based on our review, had the lowest implementation of school-based nutrition programs were also the ones harboring the highest level of undernutrition. For example, the levels of child stunting exceed 40% in Yemen (46.5%) [135] and Afghanistan (40.9%) [136], while being also high in Sudan (35%) [137], Djibouti (29.7%) [122], Somalia (23.2%) [138], Syria (22.3%) [139] and Libya (21%) [140]. The prevalence of wasting in some of these countries were in the range of 16–17% such as in Djibouti (17.8%) [122], Sudan (16.4%) [141] and Yemen (16.3%) [135]. Our observation that some countries had a relatively high implementation of school-based nutrition programs, despite their low GDP, highlight substantial inter-country differences in “will” vs. “means”. 

The objectives of the various school-based nutrition programs/interventions identified in this review have tackled the promotion of healthy dietary practices and lifestyles; reduction or prevention of undernutrition, food insecurity or hunger; reduction of micronutrient deficiencies; reduction or prevention of overweight and obesity; enhancing access to education, school enrollment, attendance and performance; and reduction of NCD risk. Less common objectives were those aimed at improving children’s cooking and/or food hygiene skills. Objectives focusing on the alleviation of undernutrition, micronutrient deficiencies or food insecurity were more commonly reported from countries that have a high burden of these public health threats, such as Afghanistan, Pakistan, Somalia, and Sudan, amongst others. In contrast, objectives focusing on reducing the risk of obesity and NCDs were more commonly reported by countries that are harboring a high burden of obesity and associated chronic diseases, such as Qatar and the UAE. Such programs could in fact play a crucial role in slowing or preventing the rise in obesity and diabetes, which is stipulated in target 7 of the “Global Action Plan for the Prevention and Control of Noncommunicable Diseases 2013–2020” [142]. The fifth recommendation of the report of the WHO Commission on Ending Childhood Obesity [143] has also focused on the implementation of interventions that promote nutrition literacy and healthy food environments in schools [8]. 

The review undertaken in this study has also identified the target population for the various school-based nutrition programs. Our findings showed that the majority of the identified programs/interventions were implemented at the levels of both schools and kindergartens. These findings are in agreement with those reported from countries in the South-East Asia Region, while interventions targeting preschoolers were less common in other regions worldwide [8]. The preschool period represents a critical period for young children to acquire and develop good dietary habits, since practices formed at this time are likely to persist in later childhood, and beyond [144,145]. Thus interventions implemented in the kindergarten setting may play an important role in helping preschoolers develop healthy dietary behaviors in the long-term [146,147]. 

Our review showed that all countries of the region (100%) had reported at least one type of school-based nutrition program, which is higher than the estimate reported by the WHO Global Nutrition report of 2016–2017 (79% of EMR countries) [8]. The 100% estimate obtained in this review results from the fact that all countries of the region had implemented school meals/school feeding programs. This proportion is higher than that reported from several other regions worldwide (74% in Africa, 70% in South-East Asian Region, 68% in America, 40% in the Western Pacific Region and 37% in Europe) [8]. School meals and school feeding programs have been described to be amongst the most impactful nutrition intervention programs [148]. A 2017 report by the Global Child Nutrition Foundation [148] indicated that the significant impact of school food programs comes from the integration of the three main pillars of development (i.e., education, health/nutrition, and agriculture) into one program, the anchoring of such programs in schools, and their intergenerational impact. The 2017 report provided an evidence-based overview, showing that school food programs are effective in addressing caloric and specific nutritional needs, improving school enrolment, attendance and retention, promoting cognitive functioning, education, and learning, as well as reducing family food insecurity and poverty [148]. 

Despite being reported from all countries of the region, there is limited information on coverage, sustainability, and up-scalability of the school feeding programs implemented in the EMR. The sustainability of these programs may in fact be the weakest in “the very places where the need is the greatest” [148], such as those countries with the highest burden of undernutrition. The main threat to their sustainability is their reliance on external support/funding, such as the support from the WFP. These programs may therefore be vulnerable to decisions that are outside the countries’ control, although there is a vital need for their continuity and enhanced effectiveness [145]. As to their impact, it is important to acknowledge that the limited data on impact from the region is not a unique observation. Due to their complexity and the effect of other factors outside the programs’ control, the measurement of the impact of school meal programs is extremely difficult, even when financial resources and expertise are available [148]. 

Although all countries of the region face a double burden of malnutrition, with persistent micronutrient deficiencies, micronutrient supplementation programs were implemented in only nine countries (Afghanistan, Bahrain, Iran, Iraq, Jordan, Pakistan, Palestine, Somalia and Sudan). Of concern, there was no evidence of implementation of such programs in other low income countries such as Egypt and Djibouti, or countries witnessing conflict or severe economic crises such as Libya, Lebanon, Syria, and Yemen. There was also no evidence of implementation or development of such programs in high income GCC countries (except for Bahrain), although several of these countries have a high burden of micronutrient deficiencies [137,149,150,151,152,153,154,155,156]. Available evidence suggests that micronutrient deficiencies can be addressed in the school setting with adequate micronutrient supplementation, especially when targeting adolescent girls [157] such as in Iran and Pakistan [28,29,30,40,109,110]. 

The findings of this review also showed that 77% of countries in the region had integrated nutrition education in the school curriculum, and this was the second most implemented type of programs in the region. This proportion is similar to that reported by other regions worldwide, such as 71% in Africa and 70% in South-East Asian [8]. Nutrition education interventions ought to be supported by appropriate staff training within the schools [8]. In the EMR, 59% of countries reported programs pertinent to training of school staff which is comparable to estimates reported from Africa (58%) and South-East Asian Region (60%), while being lower than that reported from the Western Pacific Region (68%) [8]. Evidence-based research has shown that interventions at the level of school curricula and staff training may have an impact on the knowledge, attitudes, and practices of children [158,159,160,161]. 

Taking into consideration both the fruit and vegetables offered through school meals and through separate fruit and vegetable schemes, overall, 50% of countries in the region provided fruit and vegetables in schools. This is in line with the estimate reported by the WHO Global Nutrition report of 2016–2017, where the EMR was described as the region with the lowest implementation of such schemes (46%). It can be also noted that some countries are including fresh fruit juices, which are high in free sugars, as part of the school fruit and vegetable schemes [8]. Therefore, there is a need for better advocacy and awareness amongst school authorities who are responsible for school food procurements in the various countries. The most common milk-schemes focused on full-fat or whole milk, while some have also included fortified milk. The potential to offer low fat milk was reported from KSA only [70], although available scientific evidence promotes the consumption of low fat milk in children aged above two years [162,163]. 

Although less than half of the EMR countries have developed standards for the marketing of food and beverages within the school setting (45%), this proportion was relatively higher than that reported from other regions in the world (35% in Europe, 32% in the Western Pacific Region, 30% in South-East Asian Region, 16% in America and 11% in Africa) [8]. Exposure to marketing of food and beverages within the schools is in fact a factor linked with higher food purchasing and consumption, specifically for processed foods that are high in fat, sugar and salt [164,165,166]. Food marketing was found to affect children’s food preferences and dietary intakes, and to be associated with pediatric overweight and obesity [167]. The WHO and the United Nations Children’s Fund (UNICEF) have just released, in 2023, a guidance document on the protection of children from the adverse impacts of food marketing, while adopting a “child rights-based approach” [167]. The document argues that “Children are rights holders, and governments are the corresponding duty bearers”. It quoted the Committee on the Rights of the Child, stating that “the marketing of [foods that are high in fat, sugar, or salt, energy-dense and micronutrient-poor]—especially when such marketing is focused on children–should be regulated and their availability in schools and other places controlled” [167]. The document further called for legislations and regulations governing the marketing of foods/beverages in settings where children gather, such as schools and preschool centers [167]. Evidence also shows that, for food marketing restrictions to be effective, they need to be implemented as part of a wider package of programs, which includes the regulation of the school food environment such as foods sold in the school setting [167]. Our review shows that 64% of EMR countries have reported standards pertinent to the school canteen or foods available in school shops, as compared to 77% in Europe, 68% in the Western Pacific Region, 52% in America, and 50% in South-East Asian Region and 21% in Africa [8]. The availability of healthier and more nutritious food and beverage options in schools was suggested to promote healthier eating behaviors and nutritional status amongst school-aged children [168,169]. 

School gardens were amongst the least reported interventions in countries of the EMR (32%). The Global Nutrition Policy Review 2016–2017 has also shown that the EMR had the lowest implementation of school gardens (21%) in comparison to other regions worldwide (26–60%) [8]. One of the main limitations for the implementation of school gardens may be linked to the availability of space for this type of activities [8]. However, this type of interventions ought to be encouraged where feasible, given that they provide a valuable opportunity to educate school-aged children on nutrition, and to increase their fruit and vegetable intakes. These interventions can also complement and boost the nutritional value of take-home rations or school feeding programs [8], while contributing to the promotion of more sustainable and healthier diets [8].

The implementation of clear and specific monitoring activities is essential to demonstrate program effectiveness, and to elicit greater impact. We were able to identify monitoring and process evaluation for only 21 programs in 14 countries of the region, including Afghanistan, Bahrain, Iran, Iraq, Jordan, KSA, Kuwait, Lebanon, Morocco, Oman, Pakistan, Palestine, Qatar and the UAE. Therefore, the majority of programs/interventions implemented in the region did not have clear monitoring/evaluation plans. In addition, few programs have undergone impact assessment, and few have provided information on the interventions’ duration. Given that interventions with a duration lasting longer than five months were suggested as ideal in facilitating behavioral change and positive nutrition-related outcomes [170], we made every effort in extracting information pertinent to the interventions’ duration. The few studies that have assessed the impact of intervention are those implemented in Iran, Jordan, KSA, Lebanon, Tunisia and the UAE, and their duration (when available) exceeded eight months [26,32,34,35,36,46,58,80,126,127,128,149]. The results showed a positive impact in some instances, such as the Iran Isfahan Healthy Heart Program-Heart Health Promotion from Childhood, which showed an increase in nutrition knowledge and a reduction in cardiometabolic risk factor amongst children in the intervention group [34,35,36], as well as the Ajyal Salima program in Lebanon [26], which showed a significant increase in the students’ nutritional knowledge and self-efficacy. In other instances, the investigations were not able to document an impact for the implemented nutrition programs [32,126,127,128]. For example, in Lebanon, El Halabi Ezzeddine, and Salameh [80] reported that the school health program failed to improve health behaviors amongst adolescents, including dietary practices. They indicated that, as a result of the absence of appropriate follow-up and process evaluation, and health education and services, activities in the schools became dependent to a great extent on individual initiatives and intuition [80]. Given that intuition and spontaneous initiatives cannot be duplicated, reproduced or tailored to the specific needs of student communities, such adhoc arrangements are a threat to the longevity, sustainability, and impact of the program.

Although each type of school-based interventions implemented in the region have their own positive outcomes when considered as individual components, it is the multifaceted programs that exhibit a more impactful effect, such as improved weight, dietary habits and physical activity levels in school children [8]. Comprehensive school-based nutrition programs (such as the Nutrition-Friendly Schools Initiative) tend to address multiple components such as the school food/beverage standards, and other interventions tackling the school environment, including marketing regulations and bans of vending machines. Comprehensive programs also comprise curricula on healthy diets and nutrition and staff training, while involving parents and the communities [8]. Unlike programs reported from Djibouti, Libya, Somalia, Syria and Yemen, many of the programs reported by other countries of the region were multifaceted. 

This review is characterized by a number of strengths and limitations. It is the first systematic review to identify and characterize existing government-led school nutrition programs and interventions in various countries of the EMR. It followed a systematic approach in conducting the search of published and grey literature in order to identify data from multiple sources. However, although every effort was made to locate all pertinent references and resources, the possibility of missing certain programs in the region cannot be disregarded, especially if information about these programs is not published. In addition, it is important to acknowledge that a formal evaluation of the methodological quality of studies and reports included in the review was not performed, a factor that may have influenced the study findings. Furthermore, this review was not exhaustive in its language coverage and hence articles/reports that may be available in languages other than Arabic, English or French would have been missed. Another limitation is the scarcity of information on the interventions’ duration in the various countries, and the paucity of data on the impact of the identified interventions. 

## 5. Conclusions

In summary, this review showed that all countries of the region have implemented at least one type of government-led school-based program/intervention. The most commonly implemented type of interventions were school feeding/meal programs, and the inclusion of nutrition education within the school curricula, while the least implemented were school gardens and take-home rations. The study findings showed a potential link between government effectiveness, national income and the number of government-led school-based nutrition programs in countries of the region, with the lowest number of interventions being observed in countries having the lowest GDP as well as the lowest government effectiveness score (Afghanistan, Djibouti, Libya, Somalia, Sudan, Syria and Yemen). Of concern is the fact that few programs in the region have reported monitoring and process evaluation activities, and few programs have undergone impact assessment. These findings call for the adoption of best practices for the implementation and monitoring of multi-faceted school-based nutrition programs in countries of the region, while acknowledging that this would require the elimination of country-specific barriers related to both the ‘will’ and the ‘means’ to implement school-based nutrition programs. 

## Figures and Tables

**Table 1:** Types of school-based programs/interventions implemented in each country of the EMR.

Government Effectiveness	0.48	1.44	2.88	3.37	5.29	5.77	10.1	10.58	18.27	19.71	22.12	35.58	37.5	45.67	48.08	50.96	51.44	59.62	68.75	74.04	82.69	89.9
Country *	YEM	SOM	SYR	LBY	SDN	AFG	IRQ	LBN	IRN	DJI	PSE	EGY	PAK	TUN	OMN	MOR	KWT	JOR	KSA	BHR	QAT	UAE
Take-home rations																						
School gardens																						
Bans or standards on vending machines																						
School milk scheme																						
Micronutrient supplementation (e.g., iron, vitamin a)																						
Standards for marketing of food and non-alcoholic beverage																						
Hygienic cooking facilities and clean eating environment in schools																						
School fruit and vegetable scheme																						
Training of school staff (teachers, canteen staff, school health providers etc.)																						
School canteens or standards or rules for foods and beverages available in schools																						
Extracurricular nutrition education																						
Nutrition education included in school curriculum																						
Provision of school meals, school feeding program																						

* Countries are featured based on their government effectiveness score, in ascending order. Abbreviations: EMR: Eastern Mediterranean Region; YEM: Yemen; SOM: Somalia; SYR: Syria; LBY: Libya; SDN: Sudan; AFG: Afghanistan; IRQ: Iraq; LBN: Lebanon; IRN: Iran; DJI: Djibouti; PSE: Palestine; EGY: Egypt; PAK: Pakistan; TUN: Tunisia; OMN: Oman; MOR: Morocco; KWT: Kuwait; JOR: Jordan; KSA: Kingdom of Saudi Arabia; BHR: Bahrain; QAT: Qatar; UAE: United Arab Emirates.

**Table 2:** Number of children receiving WFP school feeding programs and estimated coverage of these programs in various countries of the EMR in 2022, 2020 and 2013 *.

	Number of Children Receiving School Feeding	Estimated Coverage (%)	Number of Children Receiving School Feeding	Estimated Coverage (%)	Number of Children Receiving School Feeding	Estimated Coverage (%)
	2022		2020		2013	
Afghanistan	1,341,812	-	1,341,812	-	1,841,000	35
Bahrain	96,300	-	96,300	-	59,000	-
Djibouti	19,590	28	19,590	29	28,000	43
Egypt	11,200,000	73	11,200,000	77	7,002,000	64
Iran	2812	-	2812	-	3000	-
Iraq	350,000	-	633,351	-	555,000	11
Jordan	419,327	37	419,327	37	115,000	-
KSA	2,789,606	-	2,789,606	-	2,136,000	-
Kuwait	236,744	-	236,744	-	137,000	-
Lebanon	31,929	6	31,929	6	297,000	-
Libya	18,000	-	20,754	-	-	-
Morocco	1,267,109	28	1,267,109	29	1,423,000	31
Palestine	65,000	13	65,000	13	389,000	97
Pakistan	10,400,000	-	10,400,000	-	2,078,000	11
Oman	-	-	-	-	-	-
Qatar	130,152	-	130,152	-	57,000	-
Somalia	170,796	-	164,708	-	139,000	30
Sudan	1,890,277	39	1,361,789	27	1,630,000	34
Syria	651,728	42	1,308,648	63	46,000	2
Tunisia	350,000	20	360,000	22	240,000	-
UAE	288,795	18	821,236	85	-	-
Yemen	680,000	17	680,000	17	65,000	2

* Data based on WFP 2022 [121]. Abbreviations: EMR: Eastern Mediterranean Region; KSA: Kingdom of Saudi Arabia; UAE: United Arab Emirates; WFP: World Food Programme.

## Data Availability

All data in this review are found in the Supplementary Materials.

## Supplementary Materials

The following supporting information can be downloaded at: https://www.mdpi.com/article/10.3390/ijerph20227047/s1, Table S1: Example of a Database Search; Table S2: School-feeding and Micronutrient Supplementation Programs in Countries of the EMR; Table S3: Nutrition Education Programs in School Curricula in Countries of the EMR; Table S4: Extracurricular Nutrition Education Initiatives in Countries of the EMR; Table S5: Programs on School Staff Training in Countries of the EMR; Table S6: Programs on Marketing and Bans or Standards for Vending Machines in the School Setting in Countries of the EMR; Table S7: Fruit, Vegetable and Milk Schemes in Countries of the EMR; Table S8: Programs on School Hygienic Cooking Facilities, Clean Environments and School Gardens in Countries of the EMR; Table S9: Monitoring and Process Evaluation of School-based Programs in Countries of the EMR. File S1: Summary by source; Supplementary file: Prisma checklist.

## Abbreviations

AWRS: Arab World Research Source; EMR: Eastern Mediterranean Region; EMRO: Regional Office for the Eastern Mediterranean; ERIC: Education Resources Information Center; GCC: Gulf Cooperation Council; GDP: gross domestic products; GEI: Government Effectiveness Indicator; GINA: Global Database on the Implementation of Nutrition Action; GNI: Gross National Income; HIC: high-income oil-producing countries; HPS: Health Promoting School; IASJ: Iraqi Academic Scientific Journals; IHPS: Iranian health promoting schools; LMIC: low and medium-income countries; MNPs: micronutrient powders; MOE: Ministry of Education; NCD: non-communicable diseases; PRISMA: Preferred Reporting Items for Systematic Reviews and Meta-Analyses; SSS: School Snack Service; UAE: United Arab Emirates; UNICEF: United Nations Children’s Fund; WFP: World Food Programme; WHO: World Health Organization

## Appendix A

**Table A1:** List of search terms.

Concept 1: School	school * OR kindergarten * OR kindergarden * OR nurser * OR preschool * OR pre-school * OR “pre school *” OR pre-school * OR childcare OR “child care” OR daycare OR “day care” OR playschool * OR “senior high” OR “junior high” OR “k to 12” OR k-12
Concept 2: Nutrition	nutrition OR nutritional OR food * OR diet * OR eat OR eating OR “calor * intake” OR “energy intake” OR nutrient * OR feeding OR menu OR menus OR cafeteria * OR confection?r * OR canteen * OR vegetable * OR fruit * OR breakfast OR lunch OR meal OR meals OR snack * OR cooking
Concept 3: Intervention	interven * OR program * OR service * OR promot * OR policy OR policies OR strateg * OR initiative * OR project * OR monitor * OR assess * OR impact * OR evaluat * OR guideline * OR practice * OR legislat * OR action * OR plan OR plans OR law * OR campaign * OR marketing OR recommend * OR curriculum OR regulat *
Concept 4: EMR countries	Afghan * OR Bahrain * OR Iran * OR Persia * OR Iraq * OR Jordan * OR Kuwait * OR Lebanon * OR Lebanese OR Libya * OR Oman * OR Palestin * OR Gaza * OR “West Bank” OR Qatar * OR Saudi * OR Saudi-Arabia OR Syria * OR Tunis * OR “United Arab Emirate *” OR UAE OR Djibouti * OR Egypt * OR Morocc * OR Pakistan * OR Somal * OR Sudan * OR Yemen * OR Levant * OR “East * Mediterranean” OR “Gulf countr *” OR “Gulf Cooperation Council” OR GCC OR Arab OR Arabia OR Arabs OR EMR OR “Middle East *” OR MENA OR “North * Africa *” OR “East * Africa *” OR “Near East *” OR Dhabi OR Dabi OR Dubai OR Ajman OR Fujaira * OR Sharja * OR Khaima * OR Qaiwain * OR Quwain *

*: Truncation is a technique used in keyword searching that allows searching for various word endings simultaneously. It shortens a search term by removing the ending of a word and adding a symbol, usually an asterisk (*), to the end of the word, which helps reduce the number of variations you have to search on separately. Abbreviations: EMR: Eastern Mediterranean Region; GCC: Gulf Cooperation Council; MENA: Middle East and North Africa; UAE: United Arab Emirates.
