# Supplementary material for: School-Based Nutrition Programs in the Eastern Mediterranean Region: A Systematic Review
Source: Int J Environ Res Public Health. 2023 Nov 10;20(22):7047. doi: 10.3390/ijerph20227047 (PMC10671197; doi:10.3390/ijerph20227047)
Supplement: Supplementary file 1 [file ijerph-20-07047-s001.zip › File S1.pdf]

Search Strategies
Summary by source

Source	Date Searched	Number of Results (Including Duplicates)	Number of Results (after Removal of Duplicates)
Academic Search Ultimate	25-Oct-22	1162	341
Al Manhal	4-Nov-22	23	18
Arab World Research Source: Al Masdar	25-Oct-22	412	263
CAB Direct	25-Oct-22	1879	633
CINAHL Complete	25-Oct-22	1186	241
Cochrane	26-Oct-22	636	391
Directory of Open Access Journals	7-Nov-22	21	5
Education Research Complete	25-Oct-22	117	25
E-Marefa	7-Nov-22	23	13
Embase	28-Oct-22	2043	358
ERIC	25-Oct-22	131	99
Iraqi Academic Scientific Journals	4 and 7 November 2022	12	11
MEDLINE	25-Oct-22	1476	225
Scopus	25-Oct-22	4195	4173
Web of Science	25-Oct-22	1919	538
Google Scholar	8-Nov-22	79	41
E-mail alerts	25-Oct-22 to 16-May-23		55
Total		15,314	7430

55 articles were identified through e-mail alerts in most databases. A search of the grey literature was also conducted, using Google, the Global Database on the Implementation of Nutrition Action (GINA), the WHO EMRO (Regional Office for the Eastern Mediterranean) website and governmental websites (e.g., Ministries of Health).

A. Database searches:

Database name: Academic Search Ultimate<br>Database platform: EBSCO<br>Date searched: 25 October 2022<br>Limits applied: Time period (2000-current), Language (English, French, Arabic, multiple languages), Document type (excluded magazines, newspapers, trade publications)<br>Alert set up: Yes		
Search	Query	Number of Results
S1	TI (School* OR kindergarten* OR kindergarden* OR nurser* OR preschool* OR pre-school* OR “pre school*” OR childcare OR “child care” OR daycare OR “day care” OR playschool* OR “senior high” OR “junior high” OR “k to 12” OR k-12) OR AB (School* OR kindergarten* OR kindergarden* OR nurser* OR preschool* OR pre-school* OR “pre school*” OR childcare OR “child care” OR daycare OR “day care” OR playschool* OR “senior high” OR “junior high” OR “k to 12” OR k-12) OR KW (School* OR kindergarten* OR kindergarden* OR nurser* OR preschool* OR pre-school* OR “pre school*” OR childcare OR “child care” OR daycare OR “day care” OR playschool* OR “senior high” OR “junior high” OR “k to 12” OR k-12) OR SU (School* OR kindergarten* OR kindergarden* OR nurser* OR preschool* OR pre-school* OR “pre school*” OR childcare OR “child care” OR daycare OR “day care” OR playschool* OR “senior high” OR “junior high” OR “k to 12” OR k-12)	1,062,225
S2	TI (Nutrition OR nutritional OR food* OR diet* OR eat OR eating OR “energy intake*” OR “calor* intake*” OR nutrient* OR feeding OR menu OR menus OR cafeteria* OR confection#r* OR canteen* OR vegetable* OR fruit* OR breakfast OR lunch* OR meal OR meals OR snack* OR cooking) OR AB (Nutrition OR nutritional OR food* OR diet* OR eat OR eating OR “energy intake*” OR “calor* intake*” OR nutrient* OR feeding OR menu OR menus OR cafeteria* OR confection#r* OR canteen* OR vegetable* OR fruit* OR breakfast OR lunch* OR meal OR meals OR snack* OR cooking) OR KW (Nutrition OR nutritional OR food* OR diet* OR eat OR eating OR “energy intake*” OR “calor* intake*” OR nutrient* OR feeding OR menu OR menus OR cafeteria* OR confection#r* OR canteen* OR vegetable* OR fruit* OR breakfast OR lunch* OR meal OR meals OR snack* OR cooking) OR SU (Nutrition OR nu-tritional OR food* OR diet* OR eat OR eating OR “energy intake*” OR “calor* intake*” OR nutrient* OR feeding OR menu OR menus OR cafeteria* OR confection#r* OR canteen* OR vegetable* OR fruit* OR breakfast OR lunch* OR meal OR meals OR snack* OR cooking)	1,914,600

S3	TI (Interven* OR program* OR education* OR service* OR promot* OR policy OR policies OR strateg* OR initiative* OR project* OR monitor* OR assess* OR impact* OR evaluat* OR guideline* OR practice* OR legislat* OR action* OR plan OR plans OR law* OR campaign* OR marketing OR recommend* OR curriculum OR curricula OR regulat*) OR AB (Interven* OR program* OR education* OR service* OR promot* OR policy OR policies OR strateg* OR initiative* OR project* OR monitor* OR assess* OR impact* OR evaluat* OR guideline* OR practice* OR legislat* OR action* OR plan OR plans OR law* OR campaign* OR marketing OR recommend* OR curriculum OR curricula OR regulat*) OR KW (Interven* OR program* OR education* OR service* OR promot* OR policy OR policies OR strateg* OR initiative* OR project* OR monitor* OR assess* OR impact* OR evaluat* OR guideline* OR practice* OR legislat* OR action* OR plan OR plans OR law* OR campaign* OR marketing OR recommend* OR curriculum OR curricula OR regulat*) OR SU (Interven* OR program* OR education* OR service* OR promot* OR policy OR policies OR strateg* OR initiative* OR project* OR monitor* OR assess* OR impact* OR evaluat* OR guideline* OR practice* OR legislat* OR action* OR plan OR plans OR law* OR campaign* OR marketing OR recommend* OR curriculum OR curricula OR regulat*)	16,440,513
S4	TI (Afghan* OR Bahrain* OR Iran* OR Persia* OR Iraq* OR Jordan* OR Kuwait* OR Lebanon* OR Lebanese OR Libya* OR Oman* OR Palestin* OR Gaza* OR “West Bank” OR Qatar* OR Saudi* OR KSA OR Syria* OR Tunis* OR “United Arab Emirate*” OR UAE OR Djibouti* OR Egypt* OR Morocc* OR Pakistan* OR Somal* OR Sudan* OR Yemen* OR Levant* OR “East* Mediterranean” OR “Gulf countr*” OR “Gulf Cooperation Council” OR GCC OR Arab OR Arabia OR Arabs OR EMR OR “Middle East*” OR MENA OR “North* Africa*” OR “East* Africa*” OR “Near East*” OR Dhabi OR Dabi OR Dubai OR Ajman OR Fujaira* OR Sharja* OR Khaima* OR Qaiwain* OR Quwain*) OR AB (Afghan* OR Bahrain* OR Iran* OR Persia* OR Iraq* OR Jordan* OR Kuwait* OR Lebanon* OR Lebanese OR Libya* OR Oman* OR Palestin* OR Gaza* OR “West Bank” OR Qatar* OR Saudi* OR KSA OR Syria* OR Tunis* OR “United Arab Emirate*” OR UAE OR Djibouti* OR Egypt* OR Morocc* OR Pakistan* OR Somal* OR Sudan* OR Yemen* OR Levant* OR “East* Mediterranean” OR “Gulf countr*” OR “Gulf Cooperation Council” OR GCC OR Arab OR Arabia OR Arabs OR EMR OR “Middle East*” OR MENA OR “North* Africa*” OR “East* Africa*” OR “Near East*” OR Dhabi OR Dabi OR Dubai OR Ajman OR Fujaira* OR Sharja* OR Khaima* OR Qaiwain* OR Quwain*) OR KW (Afghan* OR Bahrain* OR Iran* OR Persia* OR Iraq* OR Jordan* OR Kuwait* OR Lebanon* OR Lebanese OR Libya* OR Oman* OR Palestin* OR Gaza* OR “West Bank” OR Qatar* OR Saudi* OR KSA OR Syria* OR Tunis* OR “United Arab Emirate*” OR UAE OR Djibouti* OR Egypt* OR Morocc* OR Pakistan* OR Somal* OR Sudan* OR Yemen* OR Levant* OR “East* Mediterranean” OR “Gulf countr*” OR “Gulf Cooperation Council” OR GCC OR Arab OR Arabia OR Arabs OR EMR OR “Middle East*” OR MENA OR “North* Africa*” OR “East* Africa*” OR “Near East*” OR Dhabi OR Dabi OR Dubai OR Ajman OR Fujaira* OR Sharja* OR Khaima* OR Qaiwain* OR Quwain*) OR SU (Afghan* OR Bahrain* OR Iran* OR Persia* OR Iraq* OR Jordan* OR Kuwait* OR Lebanon* OR Lebanese OR Libya* OR Oman* OR Palestin* OR Gaza* OR “West Bank” OR Qatar* OR Saudi* OR KSA OR Syria* OR Tunis* OR “United Arab Emirate*” OR UAE OR Djibouti* OR Egypt* OR Morocc* OR Pakistan* OR Somal* OR	820,973

	Sudan* OR Yemen* OR Levant* OR "East* Mediterranean" OR "Gulf countr*" OR "Gulf Cooperation Council" OR GCC OR Arab OR Arabia OR Arabs OR EMR OR "Middle East*" OR MENA OR "North* Africa*" OR "East* Africa*" OR "Near East*" OR Dhabi OR Dabi OR Dubai OR Ajman OR Fujaira* OR Sharja* OR Khaima* OR Qaiwain* OR Quwain*)	
S5	S1 AND S2 AND S3 AND S4	1374
S6	S5 AND Limiters—Published Date: 20000101-20221231	1351
S7	S6 AND Narrow by Language: multiple languages, Arabic, French, English<br>AND Source Types: Academic journals, Dissertations	1162
	Total (with duplicates)	1162
	Total (after removal of duplicates)	341

Database name: Al Manhal<br>Database provider: Al Manhal<br>Date searched: 4 November 2022<br>Limits applied: Time period (2000 to current)<br>Alert set up: No such feature in Al Manhal<br>Note: Did not combine with intervention concept and geographic location, screened through the results to export any relevant documents to EndNote
Search Strategy
Using Advanced search, looked for the below English and Arabic terms in the Title, Abstract, and Keyword fields (separately combining each word from concept 1 with concept 2 in each of the fields):<br>English terms:<br>*Concept 1*: school OR schools OR kindergarten OR kindergartens OR kindergarden OR kindergardens OR nursery OR nurseries OR preschool OR preschools OR preschooler OR preschoolers OR pre-school OR pre-schools OR pre-schooler OR pre-schoolers OR childcare OR child care OR daycare OR day care OR playschool OR play school OR playschools OR play schools OR senior high OR junior high OR k to 12 OR k-12

Concept 2: nutrition OR nutritional OR food OR foods OR diet OR diets OR dietary OR eat OR eating OR energy intake OR nutrient OR nutrients OR feeding OR menu OR menus OR caloric intake OR calories intake OR cafeteria OR cafeterias OR confectionary OR confectionaries OR confectionery OR confectioneries OR canteen OR canteens OR vegetable OR vegetables OR fruit OR fruits OR breakfast OR lunch OR lunches OR meal OR meals OR snack OR snacks OR snacking OR cooking

Arabic terms:

Concept 1: التدخل الغذائي OR التثقيف الغذائي

Concept 2: المدرسة OR الروضة

Total (with duplicates)	23
Total (after removal of duplicates)	18

Database name: Arab World Research Source: Al Masdar

Database platform: EBSCO

Date searched: 25 October 2022

Limits applied: Language (English, French, Arabic), Document type (excluded magazines). All documents were published post 2001.

Alert set up: Yes

Note: Did not combine with geographic location.

Search	Query	Number of Results
S1	TI (School* OR kindergarten* OR kindergarden* OR nurser* OR preschool* OR pre-school* OR “pre school*” OR childcare OR “child care” OR daycare OR “day care” OR playschool* OR “senior high” OR “junior high” OR “k to 12” OR k-12) OR AB (School* OR kindergarten* OR kindergarden* OR nurser* OR preschool* OR pre-school* OR “pre school*” OR childcare OR “child care” OR daycare OR “day care” OR playschool* OR “senior high” OR “junior high” OR “k to 12” OR k-12) OR KW (School* OR kindergarten* OR kindergarden* OR nurser* OR preschool* OR pre-school* OR “pre school*” OR childcare OR “child care” OR daycare OR “day care” OR playschool* OR “senior high” OR “junior high” OR “k to 12” OR k-12) OR SU (School* OR kindergarten* OR kindergarden* OR nurser* OR preschool* OR pre-school* OR “pre school*” OR childcare OR “child care” OR daycare OR “day care” OR playschool* OR “senior high” OR “junior high” OR “k to 12” OR k-12)	13,500

S2	TI (Nutrition OR nutritional OR food* OR diet* OR eat OR eating OR “energy intake*” OR “calor* intake*” OR nutrient* OR feeding OR menu OR menus OR cafeteria* OR confection?r* OR canteen* OR vegetable* OR fruit* OR breakfast OR lunch* OR meal OR meals OR snack* OR cooking) OR AB (Nutrition OR nutritional OR food* OR diet* OR eat OR eating OR “energy intake*” OR “calor* intake*” OR nutrient* OR feeding OR menu OR menus OR cafeteria* OR confection?r* OR canteen* OR vegetable* OR fruit* OR breakfast OR lunch* OR meal OR meals OR snack* OR cooking) OR KW (Nutrition OR nutritional OR food* OR diet* OR eat OR eating OR “energy intake*” OR “calor* intake*” OR nutrient* OR feeding OR menu OR menus OR cafeteria* OR confection?r* OR canteen* OR vegetable* OR fruit* OR breakfast OR lunch* OR meal OR meals OR snack* OR cooking) OR SU (Nutrition OR nutritional OR food* OR diet* OR eat OR eating OR “energy intake*” OR “calor* intake*” OR nutrient* OR feeding OR menu OR menus OR cafeteria* OR confection?r* OR canteen* OR vegetable* OR fruit* OR breakfast OR lunch* OR meal OR meals OR snack* OR cooking)	14,548
S3	TI (Interven* OR program* OR education* OR service* OR promot* OR policy OR policies OR strateg* OR initiative* OR project* OR monitor* OR assess* OR impact* OR evaluat* OR guideline* OR practice* OR legislat* OR action* OR plan OR plans OR law* OR campaign* OR marketing OR recommend* OR curriculum OR curricula OR regulat*) OR AB (Interven* OR program* OR education* OR service* OR promot* OR policy OR policies OR strateg* OR initiative* OR project* OR monitor* OR assess* OR impact* OR evaluat* OR guideline* OR practice* OR legislat* OR action* OR plan OR plans OR law* OR campaign* OR marketing OR recommend* OR curriculum OR curricula OR regulat*) OR KW (Interven* OR program* OR education* OR service* OR promot* OR policy OR policies OR strateg* OR initiative* OR project* OR monitor* OR assess* OR impact* OR evaluat* OR guideline* OR practice* OR legislat* OR action* OR plan OR plans OR law* OR campaign* OR marketing OR recommend* OR curriculum OR curricula OR regulat*) OR SU (Interven* OR program* OR education* OR service* OR promot* OR policy OR policies OR strateg* OR initiative* OR project* OR monitor* OR assess* OR impact* OR evaluat* OR guideline* OR practice* OR legislat* OR action* OR plan OR plans OR law* OR campaign* OR marketing OR recommend* OR curriculum OR curricula OR regulat*)	157,538
S4	S1 and S2 AND S3	313
S5	S4 AND Narrow by Language: multiple languages, Arabic, French, English<br>AND Source Types: Academic Journals	282
S6	TI (التغذية OR الغذائي OR تَغذِيَة OR غذاء) OR AB (التغذية OR الغذائي OR تَغذِيَة OR غذاء) OR KW (التغذية OR الغذائي OR تَغذِيَة OR غذاء) OR SU (التغذية OR الغذائي OR تَغذِيَة OR غذاء)	4401
S7	TI ([illegible]ضانة OR الحضانة OR الروضة OR روضة OR المدارس OR مدارس OR مدرسة OR المدرسة OR مدرسية OR المدرسية) OR AB ([illegible]ضانة OR الحضانة OR الروضة OR روضة OR المدارس OR مدارس OR مدرسة OR المدرسة OR مدرسية OR المدرسية) OR KW ([illegible]ضانة OR الحضانة OR الروضة OR روضة OR المدارس	6685

	OR مدارس OR مدرسة OR المدرسة OR مدرسية OR المدرسية) OR SU ([illegible]ضانة OR الحضانة OR الروضة OR روضة OR المدارس OR مدارس OR مدرسة OR المدرسة OR مدرسية OR المدرسية)	
S8	S6 AND S7	136
S9	S8 AND Source Types: Academic journals	130
S10	S5 OR S9	412
	Total (with duplicates)	412
	Total (after removal of duplicates)	263

Database name: CAB Direct

Database provider: CAB

Date searched: 25 October 2022

Limits applied: Time period (2000-current), Language (English, French, Arabic). Did not apply document type limit (kept all to check manually during screening)

Alert set up: Yes

Note: Libraries' subscribed products include CAB Abstracts, CAB Abstracts Archive, Global Health, CABI Full Text, CABI Reviews, CABI Reviews Archive. CAB eBooks Front File (2017-present), CAB eBooks Archive (2000-2016)

Search	Query	Number of Results
1	title:(School* OR kindergarten* OR kindergarden* OR nurser* OR preschool* OR pre-school* OR "pre school*" OR childcare OR "child care" OR daycare OR "day care" OR playschool* OR "senior high" OR "junior high" OR "k to 12" OR k-12) OR ab:(School* OR kindergarten* OR kindergarden* OR nurser* OR preschool* OR pre-school* OR "pre school*" OR childcare OR "child care" OR daycare OR "day care" OR playschool* OR "senior high" OR "junior high" OR "k to 12" OR k-12) OR subject:(School* OR kindergarten* OR kindergarden* OR nurser* OR preschool* OR pre-school* OR "pre school*" OR childcare OR "child care" OR daycare OR "day care" OR playschool* OR "senior high" OR "junior high" OR "k to 12" OR k-12)	242,194
2	title:(Nutrition OR nutritional OR food* OR diet* OR eat OR eating OR "energy intake*" OR "calor* intake*" OR nutrient* OR feeding OR menu OR menus OR cafeteria* OR confection?r* OR canteen* OR vegetable* OR fruit* OR breakfast OR lunch* OR meal OR meals OR snack* OR cooking) OR ab:(Nutrition OR nutritional OR food* OR diet* OR eat OR eating OR "energy intake*" OR "calor* intake*" OR nutrient* OR feeding OR menu OR menus OR cafeteria* OR confection?r* OR canteen* OR vegetable* OR fruit* OR breakfast OR lunch* OR meal OR meals OR snack* OR cooking) OR subject:(Nutrition OR nutritional OR food* OR diet* OR eat OR eating OR "energy intake*" OR "calor* intake*" OR nutrient* OR feeding OR menu OR menus OR cafeteria* OR confection?r* OR canteen* OR vegetable* OR fruit* OR breakfast OR lunch* OR meal OR meals OR snack* OR cooking)	3,489,578
3	title:(Interven* OR program* OR education* OR service* OR promot* OR policy OR policies OR strateg* OR initiative* OR project* OR monitor* OR assess* OR impact* OR evaluat* OR guideline* OR practice* OR legislat* OR action* OR plan OR plans OR law* OR campaign* OR marketing OR recommend* OR curriculum OR curricula OR regulat*) OR ab:(Interven* OR program* OR education* OR service* OR	6,456,752

	promot* OR policy OR policies OR strateg* OR initiative* OR project* OR monitor* OR assess* OR impact* OR evaluat* OR guideline* OR practice* OR legislat* OR action* OR plan OR plans OR law* OR campaign* OR marketing OR recommend* OR curriculum OR curricula OR regulat*) OR subject:(Interven* OR program* OR education* OR service* OR promot* OR policy OR policies OR strateg* OR initiative* OR project* OR monitor* OR assess* OR impact* OR evaluat* OR guideline* OR practice* OR legislat* OR action* OR plan OR plans OR law* OR campaign* OR marketing OR recommend* OR curriculum OR curricula OR regulat*)	
4	(gl:(Afghan* OR Bahrain* OR Iran* OR Persia* OR Iraq* OR Jordan* OR Kuwait* OR Lebanon* OR Lebanese OR Libya* OR Oman* OR Palestin* OR Gaza* OR "West Bank" OR Qatar* OR Saudi* OR KSA OR Syria* OR Tunis* OR "United Arab Emirate*" OR UAE OR Djibouti* OR Egypt* OR Morocc* OR Pakistan* OR Somal* OR Sudan* OR Yemen* OR Levant* OR "East* Mediterranean" OR "Gulf countr*" OR "Gulf Cooperation Council" OR GCC OR Arab OR Arabia OR Arabs OR EMR OR "Middle East*" OR MENA OR "North* Africa*" OR "East* Africa*" OR "Near East*" OR Dhabi OR Dabi OR Dubai OR Ajman OR Fujaira* OR Sharja* OR Khaima* OR Qaiwain* OR Quwain*)) OR (title:(Afghan* OR Bahrain* OR Iran* OR Persia* OR Iraq* OR Jordan* OR Kuwait* OR Lebanon* OR Lebanese OR Libya* OR Oman* OR Palestin* OR Gaza* OR "West Bank" OR Qatar* OR Saudi* OR KSA OR Syria* OR Tunis* OR "United Arab Emirate*" OR UAE OR Djibouti* OR Egypt* OR Morocc* OR Pakistan* OR Somal* OR Sudan* OR Yemen* OR Levant* OR "East* Mediterranean" OR "Gulf countr*" OR "Gulf Cooperation Council" OR GCC OR Arab OR Arabia OR Arabs OR EMR OR "Middle East*" OR MENA OR "North* Africa*" OR "East* Africa*" OR "Near East*" OR Dhabi OR Dabi OR Dubai OR Ajman OR Fujaira* OR Sharja* OR Khaima* OR Qaiwain* OR Quwain*) OR ab:(Afghan* OR Bahrain* OR Iran* OR Persia* OR Iraq* OR Jordan* OR Kuwait* OR Lebanon* OR Lebanese OR Libya* OR Oman* OR Palestin* OR Gaza* OR "West Bank" OR Qatar* OR Saudi* OR KSA OR Syria* OR Tunis* OR "United Arab Emirate*" OR UAE OR Djibouti* OR Egypt* OR Morocc* OR Pakistan* OR Somal* OR Sudan* OR Yemen* OR Levant* OR "East* Mediterranean" OR "Gulf countr*" OR "Gulf Cooperation Council" OR GCC OR Arab OR Arabia OR Arabs OR EMR OR "Middle East*" OR MENA OR "North* Africa*" OR "East* Africa*" OR "Near East*" OR Dhabi OR Dabi OR Dubai OR Ajman OR Fujaira* OR Sharja* OR Khaima* OR Qaiwain* OR Quwain*) OR subject:(Afghan* OR Bahrain* OR Iran* OR Persia* OR Iraq* OR Jordan* OR Kuwait* OR Lebanon* OR Lebanese OR Libya* OR Oman* OR Palestin* OR Gaza* OR "West Bank" OR Qatar* OR Saudi* OR KSA OR Syria* OR Tunis* OR "United Arab Emirate*" OR UAE OR Djibouti* OR Egypt* OR Morocc* OR Pakistan* OR Somal* OR Sudan* OR Yemen* OR Levant* OR "East* Mediterranean" OR "Gulf countr*" OR "Gulf Cooperation Council" OR GCC OR Arab OR Arabia OR Arabs OR EMR OR "Middle East*" OR MENA OR "North* Africa*" OR "East* Africa*" OR "Near East*" OR Dhabi OR Dabi OR Dubai OR Ajman OR Fujaira* OR Sharja* OR Khaima* OR Qaiwain* OR Quwain*))	481,721

5	1 AND 2 AND 3 AND 4	2587
6	5 AND Refinements: Year = 2022 OR 2021 OR 2020 OR 2019 OR 2017 OR 2018 OR 2016 OR 2015 OR 2013 OR 2014 OR 2012 OR 2011 OR 2009 OR 2010 OR 2008 OR 2007 OR 2001 OR 2005 OR 2003 OR 2006 OR 2004 OR 2002 OR 2000	2124
7	6 AND Refinements: Language = English OR Arabic OR French	1879
	Total (with duplicates)	1879
	Total (after removal of duplicates)	633

Database name: CINAHL Complete

Database platform: EBSCO

Date searched: 25 October 2022

Limits applied: Time period (2000-current), Language (English, French, Arabic, multiple languages), Document type (excluded magazines)

Alert set up: Yes

Search	Query	Number of Results
S1	TI (School* OR kindergarten* OR kindergarden* OR nurser* OR preschool* OR pre-school* OR "pre school*" OR childcare OR "child care" OR daycare OR "day care" OR playschool* OR "senior high" OR "junior high" OR "k to 12" OR k-12) OR AB (School* OR kindergarten* OR kindergarden* OR nurser* OR preschool* OR pre-school* OR "pre school*" OR childcare OR "child care" OR daycare OR "day care" OR playschool* OR "senior high" OR "junior high" OR "k to 12" OR k-12) OR SU (School* OR kindergarten* OR kindergarden* OR nurser* OR preschool* OR pre-school* OR "pre school*" OR childcare OR "child care" OR daycare OR "day care" OR playschool* OR "senior high" OR "junior high" OR "k to 12" OR k-12)	445,977
S2	TI (Nutrition OR nutritional OR food* OR diet* OR eat OR eating OR "energy intake*" OR "calor* intake*" OR nutrient* OR feeding OR menu OR menus OR cafeteria* OR confection?r* OR canteen* OR vegetable* OR fruit* OR breakfast OR lunch* OR meal OR meals OR snack* OR cooking) OR AB (Nutrition OR nutritional OR food* OR diet* OR eat OR eating OR "energy intake*" OR "calor* intake*" OR	579,192

	nutrient* OR feeding OR menu OR menus OR cafeteria* OR confection?r* OR canteen* OR vegetable* OR fruit* OR breakfast OR lunch* OR meal OR meals OR snack* OR cooking) OR SU (Nutrition OR nutritional OR food* OR diet* OR eat OR eating OR "energy intake*" OR "calor* intake*" OR nutrient* OR feeding OR menu OR menus OR cafeteria* OR confection?r* OR canteen* OR vegetable* OR fruit* OR breakfast OR lunch* OR meal OR meals OR snack* OR cooking)	
S3	TI (Interven* OR program* OR education* OR service* OR promot* OR policy OR policies OR strateg* OR initiative* OR project* OR monitor* OR assess* OR impact* OR evaluat* OR guideline* OR practice* OR legislat* OR action* OR plan OR plans OR law* OR campaign* OR marketing OR recommend* OR curriculum OR curricula OR regulat*) OR AB (Interven* OR program* OR education* OR service* OR promot* OR policy OR policies OR strateg* OR initiative* OR project* OR monitor* OR assess* OR impact* OR evaluat* OR guideline* OR practice* OR legislat* OR action* OR plan OR plans OR law* OR campaign* OR marketing OR recommend* OR curriculum OR curricula OR regulat*) OR SU (Interven* OR program* OR education* OR service* OR promot* OR policy OR policies OR strateg* OR initiative* OR project* OR monitor* OR assess* OR impact* OR evaluat* OR guideline* OR practice* OR legislat* OR action* OR plan OR plans OR law* OR campaign* OR marketing OR recommend* OR curriculum OR curricula OR regulat*)	4,635,503
S4	TI (Afghan* OR Bahrain* OR Iran* OR Persia* OR Iraq* OR Jordan* OR Kuwait* OR Lebanon* OR Lebanese OR Libya* OR Oman* OR Palestin* OR Gaza* OR "West Bank" OR Qatar* OR Saudi* OR KSA OR Syria* OR Tunis* OR "United Arab Emirate*" OR UAE OR Djibouti* OR Egypt* OR Morocc* OR Pakistan* OR Somal* OR Sudan* OR Yemen* OR Levant* OR "East* Mediterranean" OR "Gulf countr*" OR "Gulf Cooperation Council" OR GCC OR Arab OR Arabia OR Arabs OR EMR OR "Middle East*" OR MENA OR "North* Africa*" OR "East* Africa*" OR "Near East*" OR Dhabi OR Dabi OR Dubai OR Ajman OR Fujaira* OR Sharja* OR Khaima* OR Qaiwain* OR Quwain*) OR AB (Afghan* OR Bahrain* OR Iran* OR Persia* OR Iraq* OR Jordan* OR Kuwait* OR Lebanon* OR Lebanese OR Libya* OR Oman* OR Palestin* OR Gaza* OR "West Bank" OR Qatar* OR Saudi* OR KSA OR Syria* OR Tunis* OR "United Arab Emirate*" OR UAE OR Djibouti* OR Egypt* OR Morocc* OR Pakistan* OR Somal* OR Sudan* OR Yemen* OR Levant* OR "East* Mediterranean" OR "Gulf countr*" OR "Gulf Cooperation Council" OR GCC OR Arab OR Arabia OR Arabs OR EMR OR "Middle East*" OR MENA OR "North* Africa*" OR "East* Africa*" OR "Near East*" OR Dhabi OR Dabi OR Dubai OR Ajman OR Fujaira* OR Sharja* OR Khaima* OR Qaiwain* OR Quwain*) OR SU (Afghan* OR Bahrain* OR Iran* OR Persia* OR Iraq* OR Jordan* OR Kuwait* OR Lebanon* OR Lebanese OR Libya* OR Oman* OR Palestin* OR Gaza* OR "West Bank" OR Qatar* OR Saudi* OR KSA OR Syria* OR Tunis* OR "United Arab Emirate*" OR UAE OR Djibouti* OR Egypt* OR Morocc* OR Pakistan* OR Somal* OR Sudan* OR Yemen* OR Levant* OR "East* Mediterranean" OR "Gulf countr*" OR "Gulf Cooperation Council" OR GCC OR Arab OR Arabia OR Arabs OR EMR OR "Middle	109,097

	East*" OR MENA OR "North* Africa*" OR "East* Africa*" OR "Near East*" OR Dhabi OR Dabi OR Dubai OR Ajman OR Fujaira* OR Sharja* OR Khaima* OR Qaiwain* OR Quwain*)	
S5	S1 AND S2 AND S3 AND S4	1299
S6	S5 AND Narrow by Language: multiple languages, Arabic, French, English<br>AND Limiters: Published Date:20000101-20221231<br>AND Source Types: Academic journals, Dissertations	1186
	Total (with duplicates)	1186
	Total (after removal of duplicates)	241

Database name: Cochrane Library

Database provider: Cochrane Central Register of Controlled Trials (Central)

Date searched: 26 October 2022

Limits applied: Time period (2000-current), Document type (excluded 1 editorial)

Alert set up: Yes

Link to search strategy:

https://www.cochranelibrary.com/advanced-search/search-manager?search=7058116 (accessed on 26 October 2022)

Search	Query	Number of Results
#1	MeSH descriptor: [Schools] this term only	2547
#2	MeSH descriptor: [Schools, Nursery] this term only	40
#3	MeSH descriptor: [Child Day Care Centers] explode all trees	279
#4	(School* or kindergarten* or kindergarden* or nurser* or preschool* or pre-school* or "pre school*" or childcare or "child care" or daycare or "day care" or playschool* or "senior high" or "junior high" or "k to 12" or k-12):ti,ab,kw	80,660

#5	#1 OR #2 OR #3 OR #4	80,660
#6	MeSH descriptor: [Food] explode all trees	38,424
#7	MeSH descriptor: [Eating] explode all trees	3946
#8	MeSH descriptor: [Diet] explode all trees	20,453
#9	MeSH descriptor: [Food Services] this term only	275
#10	MeSH descriptor: [Menu Planning] this term only	45
#11	MeSH descriptor: [Nutritional Status] this term only	2743
#12	MeSH descriptor: [Energy Intake] explode all trees	5860
#13	(Nutrition or nutritional or food* or diet* or eat or eating or "energy intake*" or "calor* intake*" or nutrient* or feeding or menu or menus or cafeteria* or confection?r* or canteen* or vegetable* or fruit* or breakfast or lunch* or meal or meals or snack* or cooking):ti,ab,kw	190,496
#14	#6 OR #7 OR #8 OR #9 OR #10 OR #11 OR #12 OR #13	197,633
#15	MeSH descriptor: [Policy] explode all trees	895
#16	MeSH descriptor: [Health Promotion] explode all trees	7178
#17	MeSH descriptor: [Marketing] explode all trees	563
#18	MeSH descriptor: [Practice Guidelines as Topic] explode all trees	1679
#19	MeSH descriptor: [Legislation as Topic] explode all trees	664
#20	(Interven* or program* or education* or service* or promot* or policy or policies or strateg* or initiative* or project* or monitor* or assess* or impact* or evaluat* or guideline* or practice* or legislat* or action* or plan or plans or law* or campaign* or marketing or recommend* or curriculum or curricula or regulat*):ti,ab,kw	1,337,313
#21	#15 OR #16 OR #17 OR #18 OR #19 OR #20	1,337,355
#22	MeSH descriptor: [Africa, Northern] explode all trees	602
#23	MeSH descriptor: [Africa, Eastern] explode all trees	2823
#24	MeSH descriptor: [Middle East] explode all trees	3959
#25	MeSH descriptor: [Pakistan] explode all trees	538
#26	(Afghan* OR Bahrain* OR Iran* OR Persia* OR Iraq* OR Jordan* OR Kuwait* OR Lebanon* OR Lebanese OR Libya* OR Oman* OR Palestin* OR Gaza* OR "West Bank" OR Qatar* OR Saudi* OR KSA OR Syria* OR Tunis* OR "United Arab Emirate*" OR UAE OR	24,656

	Djibouti* OR Egypt* OR Morocc* OR Pakistan* OR Somal* OR Sudan* OR Yemen* OR Levant* OR “East* Mediterranean” OR “Gulf countr*” OR “Gulf Cooperation Council” OR GCC OR Arab OR Arabia OR Arabs OR EMR OR “Middle East*” OR MENA OR “North* Africa*” OR “East* Africa*” OR “Near East*” OR Dhabi OR Dabi OR Dubai OR Ajman OR Fujaira* OR Sharja* OR Khaima* OR Qaiwain* OR Quwain*):ti,ab,kw	
#27	#22 OR #23 OR #24 OR #25 OR #26	28,874
#28	#5 AND #14 AND #21 AND #27	662
#29	#5 AND #14 AND #21 AND #27 in Cochrane Reviews, Cochrane Protocols, Trials, Clinical Answers, Special Collections	661
#30	#29 with Cochrane Library publication date Between Jan 2000 and Dec 2022	636
	Total (with duplicates)	636
	Total (after removal of duplicates)	391

Database name: Directory of Open Access Journals (DOAJ)

Database manager: Infrastructure Services for Open Access C.I.C.

Date searched: 7 November 2022

Limits applied: Time period (2000 to current)

Alert set up: No such feature in DOAJ

Note: Did not combine with geographic location, screened through the results to export any relevant documents to EndNote

Search Strategy

	Title	
	Number of results	Exported
nutrition* school* intervention*	59	9
nutrition* school* program*	68	3
nutrition* school* strateg*	7	1
nutrition* school* polic*	19	0
nutrition* school* initiativ*	1	0
nutrition* school* legislat*	0	0
nutrition* school* campaign*	0	0
nutrition* school* guideline*	3	0
nutrition* school* monitor*	4	0
nutrition* school* evaluat*	17	1
nutrition* school* assess*	50	0
nutrition* school* project*	5	0
nutrition* school* service*	6	0
nutrition* school* promot*	9	1
nutrition* school* impact*	44	0
nutrition* school* practice*	21	2
nutrition* school* action*	5	0
nutrition* school* plan*	8	0
nutrition* school* law*	0	0
nutrition* school* recommend*	3	0
nutrition* school* market*	0	0
nutrition* school* curricul*	7	1
nutrition* school* regulat*	0	0
		18

	Title	
	Number of results	Exported
Nutrition* kindergarten*	12	0
School* feeding program*	45	1
Nutrition* nurser*	28	0
School* cafeteria*	9	1
kindergarten* cafeteria*	0	0
school* canteen*	58	0
school* meal*	125	0
kindergarten* meal*	3	0
preschool* meal*	13	0
nurser* meal*	6	0
school* menu*	28	0
kindergarten* menu*	2	0
preschool* menu*	2	0
nurser* menu*	0	0
school* snack*	42	1
kindergarten* snack*	2	0
preschool* snack*	8	0
nurser* snack*	0	0
		3

	Title	
	Number of results	Exported
Nutrition* preschool* intervent*	8	0
nutrition* preschool* program*	6	0
nutrition* preschool* strateg*	3	0
nutrition* preschool* polic*	0	0
nutrition* preschool* initiativ*	0	0
nutrition* preschool* legislat*	0	0
nutrition* preschool* campaign*	0	0
nutrition* preschool* guideline*	1	0
nutrition* preschool* monitor*	0	0
nutrition* preschool* evaluat*	4	0
nutrition* preschool* assess*	0	0
nutrition* preschool* project*	2	0
nutrition* preschool* service*	0	0
nutrition* preschool* promot*	1	0
nutrition* preschool* impact*	6	0
nutrition* preschool* practice*	5	0
nutrition* preschool* action*	0	0
nutrition* preschool* plan*	1	0
nutrition* preschool* law*	0	0
nutrition* preschool* recommend*	1	0
nutrition* preschool* market*	1	0
nutrition* preschool* curricul*	1	0
nutrition* preschool* regulat*	0	0
		0

Looked for the below terms in Title field only and exported to EndNote any relevant results.

Total (with duplicates)	21
Total (after removal of duplicates)	5

Database name: Education Research Complete

Database platform: EBSCO

Date searched: 25 October 2022

Limits applied: Time period (2000-current), Language (English, French, Arabic), Document type (excluded magazines, trade publications, newspapers)

Alert set up: Yes		
Search	Query	Number of Results
S1	TI (School* OR kindergarten* OR kindergarden* OR nurser* OR preschool* OR pre-school* OR "pre school*" OR childcare OR "child care" OR daycare OR "day care" OR playschool* OR "senior high" OR "junior high" OR "k to 12" OR k-12) OR AB (School* OR kindergarten* OR kindergarden* OR nurser* OR preschool* OR pre-school* OR "pre school*" OR childcare OR "child care" OR daycare OR "day care" OR playschool* OR "senior high" OR "junior high" OR "k to 12" OR k-12) OR KW (School* OR kindergarten* OR kindergarden* OR nurser* OR preschool* OR pre-school* OR "pre school*" OR childcare OR "child care" OR daycare OR "day care" OR playschool* OR "senior high" OR "junior high" OR "k to 12" OR k-12) OR SU (School* OR kindergarten* OR kindergarden* OR nurser* OR preschool* OR pre-school* OR "pre school*" OR childcare OR "child care" OR daycare OR "day care" OR playschool* OR "senior high" OR "junior high" OR "k to 12" OR k-12)	959,783
S2	TI (Nutrition OR nutritional OR food* OR diet* OR eat OR eating OR "energy intake*" OR "calor* intake*" OR nutrient* OR feeding OR menu OR menus OR cafeteria* OR confection#r* OR canteen* OR vegetable* OR fruit* OR breakfast OR lunch* OR meal OR meals OR snack* OR cooking) OR AB (Nutrition OR nutritional OR food* OR diet* OR eat OR eating OR "energy intake*" OR "calor* intake*" OR nutrient* OR feeding OR menu OR menus OR cafeteria* OR confection#r* OR canteen* OR vegetable* OR fruit* OR breakfast OR lunch* OR meal OR meals OR snack* OR cooking) OR KW (Nutrition OR nutritional OR food* OR diet* OR eat OR eating OR "energy intake*" OR "calor* intake*" OR nutrient* OR feeding OR menu OR menus OR cafeteria* OR confection#r* OR canteen* OR vegetable* OR fruit* OR breakfast OR lunch* OR meal OR meals OR snack* OR cooking) OR SU (Nutrition OR nutritional OR food* OR diet* OR eat OR eating OR "energy intake*" OR "calor* intake*" OR nutrient* OR feeding OR menu OR menus OR cafeteria* OR confection#r* OR canteen* OR vegetable* OR fruit* OR breakfast OR lunch* OR meal OR meals OR snack* OR cooking)	125,057
S3	TI (Interven* OR program* OR education* OR service* OR promot* OR policy OR policies OR strateg* OR initiative* OR project* OR monitor* OR assess* OR impact* OR evaluat* OR guideline* OR practice* OR legislat* OR action* OR plan OR plans OR law* OR campaign* OR marketing OR recommend* OR curriculum OR curricula OR regulat*) OR AB (Interven* OR program* OR education* OR service* OR promot* OR policy OR policies OR strateg* OR initiative* OR project* OR monitor* OR assess* OR impact* OR evaluat* OR guideline* OR practice* OR legislat* OR action* OR plan OR plans OR law* OR campaign* OR marketing OR recommend* OR curriculum OR curricula OR regulat*) OR KW (Interven* OR program* OR education* OR service* OR promot* OR policy OR policies OR strateg* OR initiative* OR project* OR monitor* OR assess* OR impact* OR evaluat* OR guideline* OR practice* OR legislat* OR action* OR plan OR plans OR law* OR	2,757,732

	campaign* OR marketing OR recommend* OR curriculum OR curricula OR regulat*) OR SU (Interven* OR program* OR education* OR service* OR promot* OR policy OR policies OR strateg* OR initiative* OR project* OR monitor* OR assess* OR impact* OR evaluat* OR guideline* OR practice* OR legislat* OR action* OR plan OR plans OR law* OR campaign* OR marketing OR recommend* OR curriculum OR curricula OR regulat*)	
S4	TI (Afghan* OR Bahrain* OR Iran* OR Persia* OR Iraq* OR Jordan* OR Kuwait* OR Lebanon* OR Lebanese OR Libya* OR Oman* OR Palestin* OR Gaza* OR “West Bank” OR Qatar* OR Saudi* OR KSA OR Syria* OR Tunis* OR “United Arab Emirate*” OR UAE OR Djibouti* OR Egypt* OR Morocc* OR Pakistan* OR Somal* OR Sudan* OR Yemen* OR Levant* OR “East* Mediterranean” OR “Gulf countr*” OR “Gulf Cooperation Council” OR GCC OR Arab OR Arabia OR Arabs OR EMR OR “Middle East*” OR MENA OR “North* Africa*” OR “East* Africa*” OR “Near East*” OR Dhabi OR Dabi OR Dubai OR Ajman OR Fujaira* OR Sharja* OR Khaima* OR Qaiwain* OR Quwain*) OR AB (Afghan* OR Bahrain* OR Iran* OR Persia* OR Iraq* OR Jordan* OR Kuwait* OR Lebanon* OR Lebanese OR Libya* OR Oman* OR Palestin* OR Gaza* OR “West Bank” OR Qatar* OR Saudi* OR KSA OR Syria* OR Tunis* OR “United Arab Emirate*” OR UAE OR Djibouti* OR Egypt* OR Morocc* OR Pakistan* OR Somal* OR Sudan* OR Yemen* OR Levant* OR “East* Mediterranean” OR “Gulf countr*” OR “Gulf Cooperation Council” OR GCC OR Arab OR Arabia OR Arabs OR EMR OR “Middle East*” OR MENA OR “North* Africa*” OR “East* Africa*” OR “Near East*” OR Dhabi OR Dabi OR Dubai OR Ajman OR Fujaira* OR Sharja* OR Khaima* OR Qaiwain* OR Quwain*) OR KW (Afghan* OR Bahrain* OR Iran* OR Persia* OR Iraq* OR Jordan* OR Kuwait* OR Lebanon* OR Lebanese OR Libya* OR Oman* OR Palestin* OR Gaza* OR “West Bank” OR Qatar* OR Saudi* OR KSA OR Syria* OR Tunis* OR “United Arab Emirate*” OR UAE OR Djibouti* OR Egypt* OR Morocc* OR Pakistan* OR Somal* OR Sudan* OR Yemen* OR Levant* OR “East* Mediterranean” OR “Gulf countr*” OR “Gulf Cooperation Council” OR GCC OR Arab OR Arabia OR Arabs OR EMR OR “Middle East*” OR MENA OR “North* Africa*” OR “East* Africa*” OR “Near East*” OR Dhabi OR Dabi OR Dubai OR Ajman OR Fujaira* OR Sharja* OR Khaima* OR Qaiwain* OR Quwain*) OR SU (Afghan* OR Bahrain* OR Iran* OR Persia* OR Iraq* OR Jordan* OR Kuwait* OR Lebanon* OR Lebanese OR Libya* OR Oman* OR Palestin* OR Gaza* OR “West Bank” OR Qatar* OR Saudi* OR KSA OR Syria* OR Tunis* OR “United Arab Emirate*” OR UAE OR Djibouti* OR Egypt* OR Morocc* OR Pakistan* OR Somal* OR Sudan* OR Yemen* OR Levant* OR “East* Mediterranean” OR “Gulf countr*” OR “Gulf Cooperation Council” OR GCC OR Arab OR Arabia OR Arabs OR EMR OR “Middle East*” OR MENA OR “North* Africa*” OR “East* Africa*” OR “Near East*” OR Dhabi OR Dabi OR Dubai OR Ajman OR Fujaira* OR Sharja* OR Khaima* OR Qaiwain* OR Quwain*) OR GE (Afghan* OR Bahrain* OR Iran* OR Persia* OR Iraq* OR Jordan* OR Kuwait* OR Lebanon* OR Lebanese OR Libya* OR Oman* OR Palestin* OR Gaza* OR “West Bank” OR Qatar* OR Saudi* OR KSA OR Syria* OR Tunis* OR “United Arab Emirate*” OR UAE OR Djibouti* OR Egypt* OR Morocc* OR Pakistan* OR Somal* OR Sudan* OR Yemen* OR Levant* OR “East* Mediterranean” OR “Gulf countr*” OR “Gulf Cooperation Council” OR GCC OR Arab OR Arabia OR Arabs	80,662

	OR EMR OR “Middle East*” OR MENA OR “North* Africa*” OR “East* Africa*” OR “Near East*” OR Dhabi OR Dabi OR Dubai OR Ajman OR Fujaira* OR Sharja* OR Khaima* OR Qaiwain* OR Quwain*)	
S5	S1 AND S2 AND S3 AND S4	184
S6	S5 AND Limiters: Published Date:20000101-20221231	162
S7	S6 AND Narrow by Language: Arabic, French, English	158
S8	S7 AND Narrow by Source Types: Academic journals, Conference papers	117
	Total (with duplicates)	117
	Total (after removal of duplicates)	25

Database name: e-Marefa<br>Database provider: Marefa<br>Date searched: 7 November 2022<br>Limits applied: Time period (2000 to current)<br>Alert set up: No such feature in e-Marefa<br>Note: Did not combine with geographic location, screened through the results to export any relevant documents to EndNote
Search Strategy
Looked for the below English and Arabic terms in All fields (the individual fields weren’t showing at the time of the search):

	Number of results	Exported
nutrition school intervention	19	5
nutritional school intervention	13	2
nutrition* school* program*	59	2
nutrition* school* strateg*	20	2
nutrition* school* polic*	13	0
nutrition* school* initiativ*	4	0
nutrition* school* legislat*	0	0
nutrition* school* campaign*	4	1
nutrition* school* guideline*	3	1
nutrition* school* monitor*	7	0
nutrition* school* evaluat*	48	0
nutrition* school* assess*	80	0
nutrition* school* project*	6	0
nutrition* school* service*	13	0
nutrition* school* promot*	26	2
nutrition* school* impact*	32	1
nutrition* school* practice*	19	0
nutrition* school* action*	9	0
nutrition* school* plan*	14	0
nutrition* school* law*	4	0
nutrition* school* recommend*	57	0
nutrition* school* market*	6	0
nutrition* school* curricul*	14	0
nutrition* school* regulat*	5	0
		16

	Number of results	Exported
Nutrition* kindergarten* intervent*	1	0
nutrition* kindergarten* program*	4	3
nutrition* kindergarten* strateg*	0	0
nutrition* kindergarten* polic*	0	0
nutrition* kindergarten* initiativ*	0	0
nutrition* kindergarten* legislat*	0	0
nutrition* kindergarten* campaign*	0	0
nutrition* kindergarten* guideline*	0	0
nutrition* kindergarten* monitor*	1	0
nutrition* kindergarten* evaluat*	1	0
nutrition* kindergarten* assess*	7	0
nutrition* kindergarten* project*	0	0
nutrition* kindergarten* service*	0	0
nutrition* kindergarten* promot*	1	0
nutrition* kindergarten* impact*	6	0
nutrition* kindergarten* practice*	2	1
nutrition* kindergarten* action*	0	0
nutrition* kindergarten* plan*	2	0
nutrition* kindergarten* law*	1	0
nutrition* kindergarten* recommend*	7	0
nutrition* kindergarten* market*	0	0
nutrition* kindergarten* curricul*	2	0
nutrition* kindergarten* regulat*	0	0
		4

	Number of results	Exported
Nutrition* preschool* intervent*	6	2
nutrition* preschool* program*	6	0
nutrition* preschool* strateg*	3	0
nutrition* preschool* polic*	1	0
nutrition* preschool* initiativ*	1	0
nutrition* preschool* legislat*	0	0
nutrition* preschool* campaign*	1	0
nutrition* preschool* guideline*	0	0
nutrition* preschool* monitor*	1	0
nutrition* preschool* evaluat*	8	0
nutrition* preschool* assess*	11	1
nutrition* preschool* project*	0	0
nutrition* preschool* service*	0	0
nutrition* preschool* promot*	4	0
nutrition* preschool* impact*	8	0
nutrition* preschool* practice*	4	0
nutrition* preschool* action*	1	0
nutrition* preschool* plan*	4	0
nutrition* preschool* law*	0	0
nutrition* preschool* recommend*	7	0
nutrition* preschool* market*	1	0
nutrition* preschool* curricul*	1	0
nutrition* preschool* regulat*	0	0
		3

Nutrition* nurser*	10	0
School* cafeteria*	8	0
kindergarten* cafeteria*	2	0
canteen	8	0
canteens	5	0
school* meal*	48	0
kindergarten* meal*	5	0
preschool* meal*	5	0
nurser* meal*	4	0
school* menu*	10	0
kindergarten* menu*	1	0
preschool* menu*	0	0
nurser* menu*	0	0
school* snack*	26	0
kindergarten* snack*	0	0
preschool* snack*	2	0
nurser* snack*	0	0
التدخل الغذائي	40	0
التثقيف الغذائي	43	0
		0

Total (with duplicates)	23
Total (after removal of duplicates)	13

Database name: EMBASE<br>Database platform: Elsevier<br>Date searched: 28 October 2022<br>Limits applied: Time period (2000-current), Language (English, French, Arabic), Document type (excluded Conference abstracts, conference reviews, errata, tombstone)<br>Alert set up: Yes<br>Note: Kept editorials, letters, and notes to check manually during screening		
Search	Query	Number of Results
#1	'school'/de OR 'high school'/de OR 'kindergarten'/de OR 'middle school'/de OR 'nursery school'/de OR 'primary school'/de	112,004
#2	'child day care'/exp	59
#3	school*:ti,ab,kw OR kindergarten*:ti,ab,kw OR kindergarden*:ti,ab,kw OR nurser*:ti,ab,kw OR preschool*:ti,ab,kw OR 'pre school*':ti,ab,kw OR childcare:ti,ab,kw OR 'child care':ti,ab,kw OR daycare:ti,ab,kw OR 'day care':ti,ab,kw OR playschool*:ti,ab,kw OR 'senior high':ti,ab,kw OR 'junior high':ti,ab,kw OR 'k to 12':ti,ab,kw OR 'k 12':ti,ab,kw	494,375
#4	#1 OR #2 OR #3	508,824
#5	'food'/exp	1,242,295
#6	'food intake'/exp	391,629
#7	'dietary intake'/exp	624,402
#8	'diet'/exp	404,838
#9	'dietary service'/de OR 'nutrition service'/de	813
#10	'nutritional status'/exp	79,322
#11	nutrition:ti,ab,kw OR nutritional:ti,ab,kw OR food*:ti,ab,kw OR diet*:ti,ab,kw OR eat:ti,ab,kw OR eating:ti,ab,kw OR 'energy intake*':ti,ab,kw OR 'calor* intake*':ti,ab,kw OR nutrient*:ti,ab,kw OR feeding:ti,ab,kw OR menu:ti,ab,kw OR menus:ti,ab,kw OR cafeteria*:ti,ab,kw OR confection?r*:ti,ab,kw OR canteen*:ti,ab,kw OR vegetable*:ti,ab,kw OR fruit*:ti,ab,kw OR breakfast:ti,ab,kw OR lunch*:ti,ab,kw OR meal:ti,ab,kw OR meals:ti,ab,kw OR snack*:ti,ab,kw OR cooking:ti,ab,kw	2,167,763

#12	#5 OR #6 OR #7 OR #8 OR #9 OR #10 OR #11	3,287,301
#13	‘policy’/exp	319,563
#14	‘health promotion’/exp	111,649
#15	‘practice guideline’/exp	667,488
#16	‘food legislation’/exp OR ‘law’/de OR ‘health legislation’/exp	100,647
#17	interven*:ti,ab,kw OR program*:ti,ab,kw OR education*:ti,ab,kw OR service*:ti,ab,kw OR promot*:ti,ab,kw OR policy:ti,ab,kw OR policies:ti,ab,kw OR strateg*:ti,ab,kw OR initiative*:ti,ab,kw OR project*:ti,ab,kw OR monitor*:ti,ab,kw OR assess*:ti,ab,kw OR impact*:ti,ab,kw OR evaluat*:ti,ab,kw OR guideline*:ti,ab,kw OR practice*:ti,ab,kw OR legislat*:ti,ab,kw OR action*:ti,ab,kw OR plan:ti,ab,kw OR plans:ti,ab,kw OR law*:ti,ab,kw OR campaign*:ti,ab,kw OR marketing:ti,ab,kw OR recommend*:ti,ab,kw OR curriculum:ti,ab,kw OR curricula:ti,ab,kw OR regulat*:ti,ab,kw	18,275,948
#18	#13 OR #14 OR #15 OR #16 OR #17	18,607,179
#19	‘djibouti’/de OR ‘somalia’/exp OR ‘south sudan’/de OR ‘sudan’/de OR ‘north africa’/de OR ‘egypt’/de OR ‘libyan arab jamahiriya’/de OR ‘morocco’/de OR ‘tunisia’/de OR ‘middle east’/de OR ‘bahrain’/de OR ‘iran’/de OR ‘iraq’/exp OR ‘jordan’/de OR ‘kuwait’/de OR ‘lebanon’/de OR ‘oman’/de OR ‘palestine’/de OR ‘qatar’/de OR ‘saudi arabia’/de OR ‘syrian arab republic’/de OR ‘united arab emirates’/exp OR ‘yemen’/de OR ‘pakistan’/exp OR ‘afghanistan’/de OR ‘persian gulf’/de OR ‘arab’/exp	241,450
#20	afghan*:ti,ab,kw OR bahrain*:ti,ab,kw OR iran*:ti,ab,kw OR persia*:ti,ab,kw OR iraq*:ti,ab,kw OR jordan*:ti,ab,kw OR kuwait*:ti,ab,kw OR lebanon*:ti,ab,kw OR lebanese:ti,ab,kw OR libya*:ti,ab,kw OR oman*:ti,ab,kw OR palestin*:ti,ab,kw OR gaza*:ti,ab,kw OR ‘west bank’:ti,ab,kw OR qatar*:ti,ab,kw OR saudi*:ti,ab,kw OR ksa:ti,ab,kw OR syria*:ti,ab,kw OR tunis*:ti,ab,kw OR ‘united arab emirate*’:ti,ab,kw OR uae:ti,ab,kw OR djibouti*:ti,ab,kw OR egypt*:ti,ab,kw OR morocc*:ti,ab,kw OR pakistan*:ti,ab,kw OR somal*:ti,ab,kw OR sudan*:ti,ab,kw OR yemen*:ti,ab,kw OR levant*:ti,ab,kw OR ‘east* mediterranean’:ti,ab,kw OR ‘gulf countr*’:ti,ab,kw OR ‘gulf cooperation council’:ti,ab,kw OR gcc:ti,ab,kw OR arab:ti,ab,kw OR arabia:ti,ab,kw OR arabs:ti,ab,kw OR emr:ti,ab,kw OR ‘middle east*’:ti,ab,kw OR mena:ti,ab,kw OR ‘north* africa*’:ti,ab,kw OR ‘east* africa*’:ti,ab,kw OR ‘near east*’:ti,ab,kw OR dhabi:ti,ab,kw OR dabi:ti,ab,kw OR dubai:ti,ab,kw OR ajman:ti,ab,kw OR fujaira*:ti,ab,kw OR sharja*:ti,ab,kw OR khaima*:ti,ab,kw OR qaiwain*:ti,ab,kw OR quwain*:ti,ab,kw	397,482
#21	#19 OR #20	431,887
#22	#4 AND #12 AND #18 AND #21	2547

#23	'nutrition education'/de	6564
#24	#18 AND #21 AND #23	240
#25	#22 OR #24	2737
#26	#25 AND (2000:py OR 2001:py OR 2002:py OR 2003:py OR 2004:py OR 2005:py OR 2006:py OR 2007:py OR 2008:py OR 2009:py OR 2010:py OR 2011:py OR 2012:py OR 2013:py OR 2014:py OR 2015:py OR 2016:py OR 2017:py OR 2018:py OR 2019:py OR 2020:py OR 2021:py OR 2022:py)	2628
#27	#26 AND ('Article'/it OR 'Article in Press'/it OR 'Chapter'/it OR 'Conference Paper'/it OR 'Editorial'/it OR 'Letter'/it OR 'Note'/it OR 'Review'/it OR 'Short Survey'/it)	2102
#28	#27 AND ([arabic]/lim OR [english]/lim OR [french]/lim)	2043
	Total (with duplicates)	2043
	Total (after removal of duplicates)	358

Database name: ERIC

Database platform: EBSCO

Date searched: 25 October 2022

Limits applied: Time period (2000-current). All documents were in English. Did not apply document type limit (kept all to check manually during screening).

Alert set up: Yes

Search	Query	Number of Results
S1	TI (School* OR kindergarten* OR kindergarden* OR nurser* OR preschool* OR pre-school* OR "pre school*" OR childcare OR "child care" OR daycare OR "day care" OR playschool* OR "senior high" OR "junior high" OR "k to 12" OR k-12) OR AB (School* OR kindergarten* OR kindergarden* OR nurser* OR preschool* OR pre-school* OR "pre school*" OR childcare OR "child care" OR daycare OR "day care" OR playschool* OR "senior high" OR "junior high" OR "k to 12" OR k-12) OR KW (School* OR kindergarten* OR kindergarden* OR nurser* OR preschool* OR pre-school* OR "pre school*" OR childcare OR "child care" OR daycare OR "day care" OR playschool* OR "senior high" OR	773,511

	"junior high" OR "k to 12" OR k-12) OR SU (School* OR kindergarten* OR kindergarden* OR nurser* OR preschool* OR pre-school* OR "pre school*" OR childcare OR "child care" OR daycare OR "day care" OR playschool* OR "senior high" OR "junior high" OR "k to 12" OR k-12)	
S2	TI (Nutrition OR nutritional OR food* OR diet* OR eat OR eating OR "energy intake*" OR "calor* intake*" OR nutrient* OR feeding OR menu OR menus OR cafeteria* OR confection#r* OR canteen* OR vegetable* OR fruit* OR breakfast OR lunch* OR meal OR meals OR snack* OR cooking) OR AB (Nutrition OR nutritional OR food* OR diet* OR eat OR eating OR "energy intake*" OR "calor* intake*" OR nutrient* OR feeding OR menu OR menus OR cafeteria* OR confection#r* OR canteen* OR vegetable* OR fruit* OR breakfast OR lunch* OR meal OR meals OR snack* OR cooking) OR KW (Nutrition OR nutritional OR food* OR diet* OR eat OR eating OR "energy intake*" OR "calor* intake*" OR nutrient* OR feeding OR menu OR menus OR cafeteria* OR confection#r* OR canteen* OR vegetable* OR fruit* OR breakfast OR lunch* OR meal OR meals OR snack* OR cooking) OR SU (Nutrition OR nutritional OR food* OR diet* OR eat OR eating OR "energy intake*" OR "calor* intake*" OR nutrient* OR feeding OR menu OR menus OR cafeteria* OR confection#r* OR canteen* OR vegetable* OR fruit* OR breakfast OR lunch* OR meal OR meals OR snack* OR cooking)	39,836
S3	TI (Interven* OR program* OR education* OR service* OR promot* OR policy OR policies OR strateg* OR initiative* OR project* OR monitor* OR assess* OR impact* OR evaluat* OR guideline* OR practice* OR legislat* OR action* OR plan OR plans OR law* OR campaign* OR marketing OR recommend* OR curriculum OR curricula OR regulat*) OR AB (Interven* OR program* OR education* OR service* OR promot* OR policy OR policies OR strateg* OR initiative* OR project* OR monitor* OR assess* OR impact* OR evaluat* OR guideline* OR practice* OR legislat* OR action* OR plan OR plans OR law* OR campaign* OR marketing OR recommend* OR curriculum OR curricula OR regulat*) OR KW (Interven* OR program* OR education* OR service* OR promot* OR policy OR policies OR strateg* OR initiative* OR project* OR monitor* OR assess* OR impact* OR evaluat* OR guideline* OR practice* OR legislat* OR action* OR plan OR plans OR law* OR campaign* OR marketing OR recommend* OR curriculum OR curricula OR regulat*) OR SU (Interven* OR program* OR education* OR service* OR promot* OR policy OR policies OR strateg* OR initiative* OR project* OR monitor* OR assess* OR impact* OR evaluat* OR guideline* OR practice* OR legislat* OR action* OR plan OR plans OR law* OR campaign* OR marketing OR recommend* OR curriculum OR curricula OR regulat*)	1,695,257
S4	TI (Afghan* OR Bahrain* OR Iran* OR Persia* OR Iraq* OR Jordan* OR Kuwait* OR Lebanon* OR Lebanese OR Libya* OR Oman* OR Palestin* OR Gaza* OR "West Bank" OR Qatar* OR Saudi* OR KSA OR Syria* OR Tunis* OR "United Arab Emirate*" OR UAE OR Djibouti* OR Egypt* OR Morocc* OR Pakistan* OR Somal* OR Sudan* OR Yemen* OR Levant* OR "East* Mediterranean" OR "Gulf countr*" OR "Gulf	23,991

	Cooperation Council" OR GCC OR Arab OR Arabia OR Arabs OR EMR OR "Middle East*" OR MENA OR "North* Africa*" OR "East* Africa*" OR "Near East*" OR Dhabi OR Dabi OR Dubai OR Ajman OR Fujaira* OR Sharja* OR Khaima* OR Qaiwain* OR Quwain*) OR AB (Afghan* OR Bahrain* OR Iran* OR Persia* OR Iraq* OR Jordan* OR Kuwait* OR Lebanon* OR Lebanese OR Libya* OR Oman* OR Palestin* OR Gaza* OR "West Bank" OR Qatar* OR Saudi* OR KSA OR Syria* OR Tunis* OR "United Arab Emirate*" OR UAE OR Djibouti* OR Egypt* OR Morocc* OR Pakistan* OR Somal* OR Sudan* OR Yemen* OR Levant* OR "East* Mediterranean" OR "Gulf countr*" OR "Gulf Cooperation Council" OR GCC OR Arab OR Arabia OR Arabs OR EMR OR "Middle East*" OR MENA OR "North* Africa*" OR "East* Africa*" OR "Near East*" OR Dhabi OR Dabi OR Dubai OR Ajman OR Fujaira* OR Sharja* OR Khaima* OR Qaiwain* OR Quwain*) OR KW (Afghan* OR Bahrain* OR Iran* OR Persia* OR Iraq* OR Jordan* OR Kuwait* OR Lebanon* OR Lebanese OR Libya* OR Oman* OR Palestin* OR Gaza* OR "West Bank" OR Qatar* OR Saudi* OR KSA OR Syria* OR Tunis* OR "United Arab Emirate*" OR UAE OR Djibouti* OR Egypt* OR Morocc* OR Pakistan* OR Somal* OR Sudan* OR Yemen* OR Levant* OR "East* Mediterranean" OR "Gulf countr*" OR "Gulf Cooperation Council" OR GCC OR Arab OR Arabia OR Arabs OR EMR OR "Middle East*" OR MENA OR "North* Africa*" OR "East* Africa*" OR "Near East*" OR Dhabi OR Dabi OR Dubai OR Ajman OR Fujaira* OR Sharja* OR Khaima* OR Qaiwain* OR Quwain*) OR SU (Afghan* OR Bahrain* OR Iran* OR Persia* OR Iraq* OR Jordan* OR Kuwait* OR Lebanon* OR Lebanese OR Libya* OR Oman* OR Palestin* OR Gaza* OR "West Bank" OR Qatar* OR Saudi* OR KSA OR Syria* OR Tunis* OR "United Arab Emirate*" OR UAE OR Djibouti* OR Egypt* OR Morocc* OR Pakistan* OR Somal* OR Sudan* OR Yemen* OR Levant* OR "East* Mediterranean" OR "Gulf countr*" OR "Gulf Cooperation Council" OR GCC OR Arab OR Arabia OR Arabs OR EMR OR "Middle East*" OR MENA OR "North* Africa*" OR "East* Africa*" OR "Near East*" OR Dhabi OR Dabi OR Dubai OR Ajman OR Fujaira* OR Sharja* OR Khaima* OR Qaiwain* OR Quwain*)	
S5	S1 AND S2 AND S3 AND S4	223
S6	S5 AND Limiters: Published Date:20000101-20211231 (most recent document was published in 2021)	131
	Total (with duplicates)	131
	Total (after removal of duplicates)	99

Database name: Iraqi Academic Scientific Journals (IASJ)

Database provider: Ministry of Higher Education & Scientific Research of Iraq

Date searched: 4 and 7 November 2022

Limits applied: Time period (2000 to current)

Alert set up: No such feature in IASJ

Note: Did not combine with intervention concept and geographic location, screened through the results to export any relevant documents to EndNote

Search Strategy

Using Advanced search, looked for the below English and Arabic terms in the Title, Abstract, and Keyword fields (separately combining each word from concept 1 with concept 2 in each of the fields):

English terms:

Concept 1: school OR schools OR kindergarten OR kindergartens OR kindergarden OR kindergardens OR nursery OR nurseries OR preschool OR preschools OR preschooler OR preschoolers OR pre-school OR pre-schools OR pre-schooler OR pre-schoolers OR childcare OR child care OR daycare OR day care OR playschool OR play school OR playschools OR play schools OR senior high OR junior high OR k to 12 OR k-12

Concept 2: nutrition OR nutritional OR food OR foods OR diet OR diets OR dietary OR eat OR eating OR energy intake OR nutrient OR nutrients OR feeding OR menu OR menus OR caloric intake OR calories intake OR cafeteria OR cafeterias OR confectionary OR confectionaries OR confectionery OR confectioneries OR canteen OR canteens OR vegetable OR vegetables OR fruit OR fruits OR breakfast OR lunch OR lunches OR meal OR meals OR snack OR snacks OR snacking OR cooking

Arabic terms:

Concept 1: التدخل الغذائي OR التثقيف الغذائي

Concept 2: المدرسة OR الروضة

Total (with duplicates)	12
Total (after removal of duplicates)	11

Database name: MEDLINE

Database platform: Ovid

Date searched: 25 October 2022

Limits applied: Time period (2000-current), Language (English, French, Arabic). Did not apply document type limit (kept all to check manually during screening)

Alert set up: Yes

Link to search strategy: https://ovidsp.ovid.com/ovidweb.cgi?T=JS&NEWS=N&PAGE=main&SHAREDSEARCHID=5vKN4LM4Zh448lGOuVguxSW9Q8ELWBKuYQV5rYnxRFETeFGAMY4hQTxyS2fcPK41a (accessed on 25 October 2022) Note: OVID MEDLINE® and Epub Ahead of Print, In-Process, In-Data-Review & Other Non-Indexed Citations and Daily; 1946 to October 24, 2022		
Search	Query	Number of Results
1	schools/ or schools, nursery/	50,429
2	exp Child Day Care Centers/	6245
3	(School* or kindergarten* or kindergarden* or nurser* or preschool* or pre-school* or “pre school*” or childcare or “child care” or daycare or “day care” or playschool* or “senior high” or “junior high” or “k to 12” or k-12).ti,ab.	391,599
4	1 or 2 or 3	398,506
5	exp Food/	1,432,568
6	exp Eating/	79,239
7	exp Diet/	319,942
8	food services/ or menu planning/	7130
9	Nutritional Status/	52,215
10	exp Energy Intake/	50,569
11	(Nutrition or nutritional or food* or diet* or eat or eating or “energy intake*” or “calor* intake*” or nutrient* or feeding or menu or menus or cafeteria* or confection?r* or canteen* or vegetable* or fruit* or breakfast or lunch* or meal or meals or snack* or cooking).ti,ab.	1,718,886
12	5 or 6 or 7 or 8 or 9 or 10 or 11	2,785,114
13	exp policy/	172,676
14	exp Health Promotion/	83,993
15	exp guideline/	37,298
16	legislation/	1672
17	Legislation, Food/	2485

18	(Interven* or program* or education* or service* or promot* or policy or policies or strateg* or initiative* or project* or monitor* or assess* or impact* or evaluat* or guideline* or practice* or legislat* or action* or plan or plans or law* or campaign* or marketing or recommend* or curriculum or curricula or regulat*).ti,ab.	13,925,126
19	13 or 14 or 15 or 16 or 17 or 18	14,008,356
20	africa, northern/ or egypt/ or libya/ or morocco/ or tunisia/ or djibouti/ or somalia/ or south sudan/ or sudan/ or middle east/ or afghanistan/ or bahrain/ or iran/ or iraq/ or jordan/ or kuwait/ or lebanon/ or oman/ or qatar/ or saudi arabia/ or syria/ or united arab emirates/ or yemen/ or pakistan/ or africa, eastern/	156,607
21	(Afghan* OR Bahrain* OR Iran* OR Persia* OR Iraq* OR Jordan* OR Kuwait* OR Lebanon* OR Lebanese OR Libya* OR Oman* OR Palestin* OR Gaza* OR "West Bank" OR Qatar* OR Saudi* OR KSA OR Syria* OR Tunis* OR "United Arab Emirate*" OR UAE OR Djibouti* OR Egypt* OR Morocc* OR Pakistan* OR Somal* OR Sudan* OR Yemen* OR Levant* OR "East* Mediterranean" OR "Gulf countr*" OR "Gulf Cooperation Council" OR GCC OR Arab OR Arabia OR Arabs OR EMR OR "Middle East*" OR MENA OR "North* Africa*" OR "East* Africa*" OR "Near East*" OR Dhabi OR Dabi OR Dubai OR Ajman OR Fujaira* OR Sharja* OR Khaima* OR Qaiwain* OR Quwain*).ti,ab	288,527
22	20 or 21	319,200
23	4 and 12 and 19 and 22	1716
24	limit 23 to yr="2000 -Current"	1482
25	limit 24 to (arabic or english or french)	1476
	Total (with duplicates)	1476
	Total (after removal of duplicates)	225

Database name: Scopus

Database provider: Elsevier

Date searched: 25 October 2022

Limits applied: Time period (2000-current), Language (English, French, Arabic, Undefined), Document type (excluded errata, conference reviews, editorials, retracted)

Alert set up: Yes

Search	Query	Number of Results

1	School* OR kindergarten* OR kindergarden* OR nurser* OR preschool* OR pre-school* OR “pre school*” OR pre-school* OR childcare OR “child care” OR daycare OR “day care” OR playschool* OR “senior high” OR “junior high” OR “k to 12” OR k-12	2,350,457
2	Nutrition OR nutritional OR food* OR diet* OR eat OR eating OR “calor* intake” OR “energy intake” OR nutrient* OR feeding OR menu OR menus OR cafeteria* OR confection?r* OR canteen* OR vegetable* OR fruit* OR breakfast OR lunch OR meal OR meals OR snack* OR cooking	4,345,802
3	Interven* OR program* OR service* OR promot* OR policy OR policies OR strateg* OR initiative* OR project* OR monitor* OR assess* OR impact* OR evaluat* OR guideline* OR practice* OR legislat* OR action* OR plan OR plans OR law* OR campaign* OR marketing OR recommend* OR curriculum OR regulat*	35,342,565
4	Afghan* OR Bahrain* OR Iran* OR Persia* OR Iraq* OR Jordan* OR Kuwait* OR Lebanon* OR Lebanese OR Libya* OR Oman* OR Palestin* OR Gaza* OR “West Bank” OR Qatar* OR Saudi* OR KSA OR Syria* OR Tunis* OR “United Arab Emirate*” OR UAE OR Djibouti* OR Egypt* OR Morocc* OR Pakistan* OR Somal* OR Sudan* OR Yemen* OR Levant* OR “East* Mediterranean” OR “Gulf countr*” OR “Gulf Cooperation Council” OR GCC OR Arab OR Arabia OR Arabs OR EMR OR “Middle East*” OR MENA OR “North* Africa*” OR “East* Africa*” OR “Near East*” OR Dhabi OR Dabi OR Dubai OR Ajman OR Fujaira* OR Sharja* OR *Khaima* OR *Qaiwain* OR *Quwain*	996,339
5	1 AND 2 AND 3 AND 4	4953
6	#5 AND (LIMIT-TO (PUBYEAR , 2023) OR LIMIT-TO (PUBYEAR , 2022) OR LIMIT-TO (PUBYEAR , 2021) OR LIMIT-TO (PUBYEAR , 2020) OR LIMIT-TO (PUBYEAR , 2019) OR LIMIT-TO (PUBYEAR , 2018) OR LIMIT-TO (PUBYEAR , 2017) OR LIMIT-TO (PUBYEAR , 2016) OR LIMIT-TO (PUBYEAR , 2015) OR LIMIT-TO (PUBYEAR , 2014) OR LIMIT-TO (PUBYEAR , 2013) OR LIMIT-TO (PUBYEAR , 2012) OR LIMIT-TO (PUBYEAR , 2011) OR LIMIT-TO (PUBYEAR , 2010) OR LIMIT-TO (PUBYEAR , 2009) OR LIMIT-TO (PUBYEAR , 2008) OR LIMIT-TO (PUBYEAR , 2007) OR LIMIT-TO (PUBYEAR , 2006) OR LIMIT-TO (PUBYEAR , 2005) OR LIMIT-TO (PUBYEAR , 2004) OR LIMIT-TO (PUBYEAR , 2003) OR LIMIT-TO (PUBYEAR , 2002) OR LIMIT-TO (PUBYEAR , 2001) OR LIMIT-TO (PUBYEAR , 2000)) AND (EXCLUDE (DOCTYPE , “ed”) OR EXCLUDE (DOCTYPE , “cr”) OR EXCLUDE (DOCTYPE , “er”) OR EXCLUDE (DOCTYPE , “tb”)) AND (LIMIT-TO (LANGUAGE , “English”) OR LIMIT-TO (LANGUAGE , “French”) OR LIMIT-TO (LANGUAGE , “Arabic”) OR LIMIT-TO (LANGUAGE , “Undefined”))	4195
	Total (with duplicates)	4195
	Total (after removal of duplicates)	4173

Database name: Web of Science Core Collection<br>Database provider: Clarivate Analytics<br>Date searched: 25 October 2022<br>Limits applied: Time period (2000-current), Language (English, French, Arabic), Document type (excluded meeting abstracts, editorial materials, retracted)<br>Alert set up: Yes<br>Link to search strategy:<br>https://www.webofscience.com/wos/woscc/summary/f593e6ef-424a-44cf-a030-ec07a44adbbb-5b59e3b5/relevance/1 (accessed on 25 october 2022)<br>Note: Libraries' subscribed products include Social Sciences Citation Index (SSCI) 1900 to 2022, Arts & Humanities Citation Index (AHCI) 1975 to 2022, Conference Proceedings Citation Index—Science (ISTP) 1990 to 2022, Emerging Sources Citation Index (ESCI) 2017 to 2022, Science Citation Index Expanded (SCI) 1900 to 2022, Conference Proceedings Citation Index—Social Sciences (ISSHP) 1990 to 2022		
Search	Query	Number of Results
1	TS=(School* OR kindergarten* OR kindergarden* OR nurser* OR preschool* OR pre-school* OR "pre school*" OR pre-school* OR childcare OR "child care" OR daycare OR "day care" OR playschool* OR "senior high" OR "junior high" OR "k to 12" OR k-12)	870,818
2	TS=(Nutrition OR nutritional OR food* OR diet* OR eat OR eating OR "energy intake" OR "calor* intake" OR nutrient* OR feeding OR menu OR menus OR cafeteria* OR confection?r* OR canteen* OR vegetable* OR fruit* OR breakfast OR lunch OR meal OR meals OR snack* OR cooking)	3,486,484
3	TS=(Interven* OR program* OR education* OR service* OR promot* OR policy OR policies OR strateg* OR initiative* OR project* OR monitor* OR assess* OR impact* OR evaluat* OR guideline* OR practice* OR legislat* OR action* OR plan OR plans OR law* OR campaign* OR marketing OR recommend* OR curriculum OR regulat*)	25,714,123
4	TS=(Afghan* OR Bahrain* OR Iran* OR Persia* OR Iraq* OR Jordan* OR Kuwait* OR Lebanon* OR Lebanese OR Libya* OR Oman* OR Palestin* OR Gaza* OR "West Bank" OR Qatar* OR Saudi* OR KSA OR Syria* OR Tunis* OR "United Arab Emirate*" OR UAE OR Djibouti* OR Egypt* OR Morocc* OR Pakistan* OR Somal* OR Sudan* OR Yemen* OR Levant* OR "East* Mediterranean" OR "Gulf countr*" OR	740,334

	"Gulf Cooperation Council" OR GCC OR Arab OR Arabia OR Arabs OR EMR OR "Middle East*" OR MENA OR "North* Africa*" OR "East* Africa*" OR "Near East*" OR Dhabi OR Dabi OR Dubai OR Ajman OR Fujaira* OR Sharja* OR *Khaima* OR *Qaiwain* OR *Quwain*)	
5	1 AND 2 AND 3 AND 4	2023
6	5 AND 2022 or 2021 or 2020 or 2019 or 2018 or 2017 or 2016 or 2015 or 2014 or 2013 or 2011 or 2012 or 2010 or 2009 or 2008 or 2007 or 2006 or 2005 or 2004 or 2003 or 2002 or 2001 or 2000 (Publication Years) and Meeting Abstract or Editorial Material or Retracted Publication (Exclude—Document Types) and English or French or Arabic (Languages)	1919
	Total (with duplicates)	1919
	Total (after removal of duplicates)	538

*: Truncation is a technique used in keyword searching that allows searching for various word endings simultaneously. It shortens a search term by removing the ending of a word and adding a symbol, usually an asterisk (*), to the end of the word, which helps reduce the number of variations you have to search on separately.

B. Search engines:

Search engine name: Google Scholar (https://scholar.google.com/) (accessed on 8 November 2022)<br>Search engine provider: Google<br>Date searched: 8 November 2022<br>Limits applied: Time period (2000-current)<br>Note: Did not combine with geographic location, screened through the results to export any relevant documents to EndNote<br>Alert set up: No
Search Strategy
Using Advanced search, looked for the below phrases in the title field and checked if related to EMR countries.<br>– allintitle: school nutrition intervention<br>– allintitle: school nutrition interventions<br>– allintitle: school nutrition program<br>– allintitle: school nutrition programs

– allintitle: kindergarten nutrition intervention<br>– allintitle: kindergarten nutrition interventions<br>– allintitle: kindergarten nutrition program<br>– allintitle: kindergarten nutrition programs<br>– allintitle: school healthy eating<br>– allintitle: canteen nutrition<br>– allintitle: preschool nutrition intervention<br>– allintitle: preschool nutrition interventions<br>– allintitle: school nutrition policy<br>– allintitle: school nutrition policies<br>– allintitle: nursery intervention<br>– allintitle: nursery interventions	
Total (with duplicates)	79
Total (after removal of duplicates)	41

Additional Notes

**1.**	**Indicate process for removing duplicates.**	Results were de-duplicated using EndNote followed by manual double checking
**2.**	**List filters or limits applied.**	Language = English, French, Arabic (kept undefined or undetermined or multiple languages to check during screening)<br>Publication date: 2000 to present<br>Document types: Magazines, newspapers, trade publications, conference or meeting abstracts, conference reviews, retractions, tombstones, errata, editorials, letters, notes (after checking)
**3.**	**Was this search based on a previous search such as a search used in a prior review?**<br>**(If yes, provide citation or source.)**	No
**4.**	**Was this search peer-reviewed? If so, by whom and what are their qualifications and area of expertise?**	No
**5.**	**Other Notes**	N/A
