# Supplementary material for: School-Based Nutrition Programs in the Eastern Mediterranean Region: A Systematic Review
Source: Int J Environ Res Public Health. 2023 Nov 10;20(22):7047. doi: 10.3390/ijerph20227047 (PMC10671197; doi:10.3390/ijerph20227047)
Supplement: Supplementary file 1 [file ijerph-20-07047-s001.zip › Table S1.pdf]

Database name: *MEDLINE*

Database platform: *Ovid*

Date searched: *October 25, 2022*

Limits applied: *Time period (2000-current), Language (English, French, Arabic). Did not apply document type limit (kept all to check manually during screening)*

Alert set up: *Yes*

Link to search strategy:
https://ovidsp.ovid.com/ovidweb.cgi?T=JS&NEWS=N&PAGE=main&SHAREDSEARCHID=5vKN4LM4Zh448lGOuVguxSW9Q8ELWBKuYQV5rYnxRFETeFGAMY4hQTxyS2fcPK41a

Note: *OVID MEDLINE® and Epub Ahead of Print, In-Process, In-Data-Review & Other Non-Indexed Citations and Daily; 1946 to October 24, 2022*

Search	Query	Number of Results
1	schools/ or schools, nursery/	50,429
2	exp Child Day Care Centers/	6,245
3	(School* or kindergarten* or kindergarden* or nurser* or preschool* or pre-school* or "pre school*" or childcare or "child care" or daycare or "day care" or playschool* or "senior high" or "junior high" or "k to 12" or k-12).ti,ab.	391,599
4	1 or 2 or 3	398,506
5	exp Food/	1,432,568
6	exp Eating/	79,239
7	exp Diet/	319,942
8	food services/ or menu planning/	7,130
9	Nutritional Status/	52,215
10	exp Energy Intake/	50,569
11	(Nutrition or nutritional or food* or diet* or eat or eating or "energy intake*" or "calor* intake*" or nutrient* or feeding or menu or menus or cafeteria* or confection?r* or canteen* or vegetable* or fruit* or breakfast or lunch* or meal or meals or snack* or cooking).ti,ab.	1,718,886
12	5 or 6 or 7 or 8 or 9 or 10 or 11	2,785,114
13	exp policy/	172,676
14	exp Health Promotion/	83,993
15	exp guideline/	37,298
16	legislation/	1,672
17	Legislation, Food/	2,485
18	(Interven* or program* or education* or service* or promot* or policy or policies or strateg* or initiative* or project* or monitor* or assess* or impact* or evaluat* or guideline* or practice* or legislat* or action* or plan or plans or law* or campaign* or marketing or recommend* or curriculum or curricula or regulat*).ti,ab.	13,925,126
19	13 or 14 or 15 or 16 or 17 or 18	14,008,356
20	africa, northern/ or egypt/ or libya/ or morocco/ or tunisia/ or djibouti/ or somalia/ or south sudan/ or sudan/ or middle east/ or afghanistan/ or bahrain/ or iran/ or iraq/ or	156,607

	jordan/ or kuwait/ or lebanon/ or oman/ or qatar/ or saudi arabia/ or syria/ or united arab emirates/ or yemen/ or pakistan/ or africa, eastern/	
21	(Afghan* OR Bahrain* OR Iran* OR Persia* OR Iraq* OR Jordan* OR Kuwait* OR Lebanon* OR Lebanese OR Libya* OR Oman* OR Palestin* OR Gaza* OR "West Bank" OR Qatar* OR Saudi* OR KSA OR Syria* OR Tunis* OR "United Arab Emirate*" OR UAE OR Djibouti* OR Egypt* OR Morocc* OR Pakistan* OR Somal* OR Sudan* OR Yemen* OR Levant* OR "East* Mediterranean" OR "Gulf countr*" OR "Gulf Cooperation Council" OR GCC OR Arab OR Arabia OR Arabs OR EMR OR "Middle East*" OR MENA OR "North* Africa*" OR "East* Africa*" OR "Near East*" OR Dhabi OR Dabi OR Dubai OR Ajman OR Fujaira* OR Sharja* OR Khaima* OR Qaiwain* OR Quwain*).ti,ab	288,527
22	20 or 21	319,200
23	4 and 12 and 19 and 22	1,716
24	limit 23 to yr="2000 -Current"	1,482
25	limit 24 to (arabic or english or french)	1,476
	**Total (with duplicates)**	**1,476**
	**Total (after removal of duplicates)**	**225**
