# Supplementary material for: School-Based Nutrition Programs in the Eastern Mediterranean Region: A Systematic Review
Source: Int J Environ Res Public Health. 2023 Nov 10;20(22):7047. doi: 10.3390/ijerph20227047 (PMC10671197; doi:10.3390/ijerph20227047)
Supplement: Supplementary file 1 [file ijerph-20-07047-s001.zip › Table S2.pdf]

Table S2. School-feeding and Micronutrient Supplementation Programs in Countries of the EMR

Country	Reference	Year and Status	National or Regional	Leadership	Target Population	Objective	Brief Description of the Policy/Intervention
**Provision of school meals, school feeding programme**							
**EMR**	Al-Jawaldeh et al 2020 [1]	-	-	-	Schools	-Prevent undernutrition and overweight or obesity.	9 countries were providing school meals or implemented school feeding programmes
**Afghanistan**	Hees and Sankei 2013 [2]	2009-2010	Regional (8 provinces)	WFP's Afghanistan Protracted Relief Operation (under Food and Education) – in coordination with concerned governmental entities	Students in grades one through nine. 54 schools in 15 food-insecure districts across eight provinces, targeting 46,000 children	- Increase access to education for girls and boys in WFP-assisted schools.<br>- Address micronutrient deficiencies through the provision of food fortified with micronutrient powder (MNP) among 46,000 students in grades one through nine.	Each child takes home the wheat flour, which is used by their parents at home to bake bread that is then taken to school by the child every day.
**Bahrain**	Garemo et al 2019 [3]; Musaiger et al 2011 [4]; WHO 2013 [5]	2014	National	MOH	Kindergartens, primary and secondary schools	Improve the nutrition status of children in Bahrain.	School feeding programme; provision of school meals based on national dietary guidelines
**Egypt**	Al-Jawaldeh et al 2020 [1]; WHO 2018 [6]	-	National	MOH and MOE	Kindergartens and schools	- Reduce or prevent child undernutrition (stunting, wasting, micronutrient deficiencies).<br>- Reduce or prevent childhood overweight or obesity.<br>- Foster healthy diet and lifestyle habits.	Provide free school meals for all children.<br>- Menus are decided according to minimum levels of specific nutrients, following national dietary guidelines.<br>- At the regional or national level, menus are decided by a

						- Educate children and improve knowledge about healthy diet and lifestyle habits.<br>-Improve children's skills (e.g. cooking, food hygiene).<br>- Improve school enrolment.<br>- Improve school attendance.<br>- Improve academic performance.<br>- Tackle health inequalities.<br>- Reduce food insecurity and hunger.	nutritionist or other health worker.
	Information provided by NFP; Ministry of Education and Technical Education [7]	2019	National	MOH and Ministry of Education and Technical Education	School students, with a focus on those in kindergarten and primary school	- Improve the healthy nutrition of school students to treat and prevent diseases resulting from malnutrition.<br>- Intensify awareness about healthy, useful eating, and healthy meals appropriate for the appropriate age group.<br>- Provide school meals characterized by security, safety, diversity, the availability of health conditions, and nutritional needs, with an emphasis on quality.	**100 Million Health Initiative:**<br>- Provide healthy meals for school students.<br>- Implement a school feeding program for all kindergarten and primary school students for the age group from 4 to 12 years in schools.
	Garemo et al 2019 [3]	-	-	MOH, with various stakeholders and NGOs	Kindergarten children	- Promote healthy dietary practices and life styles among school aged children.	A school-based meal program provides daily meals to all kindergarten children who require and qualify for it.
	WFP 2022 [8]	2014-2030	National; 11 governorates	WFP – in coordination with concerned	Schools	- Increase the nutritional value of school meals.	WFP provided daily, in-school snacks (fortified date-bars) to 119,000 community school students across the 11

				governmental entities			governorates. Additionally, WFP provided cash assistance to about 21,000 families of community school children to support their food security.
	Metwally et al 2020 [9]; Salah et al 2012[10]	1998<br><br>Total duration (> 5 years)	National	Ministry of Agriculture and Land Reclamation	Schools	- Reduce hunger for millions of children and contribute to better student behavior and attentiveness.	**School Feeding Program in Egypt**<br>- Covers 13.5 million children of all ages.<br>- Attracts children in rural areas to attend primary schools by providing them with nutritious fortified snacks as a contribution to breakfast consumption.<br>- Currently, 30% of the total student population, or 5 million students, participate in Egypt's school feeding program.
**Iran**	WHO 2018 [6]	2007	National	MOE and MOH	Kindergartens and schools	- Reduce or prevent child undernutrition (stunting, wasting, micronutrient deficiencies).<br>- Reduce or prevent childhood overweight or obesity.<br>- Foster healthy diet and lifestyle habits.<br>- Educate children and improve knowledge about healthy diet and lifestyle habits.<br>- Tackle health inequalities.<br>- Reduce food insecurity and hunger.	School feeding programmes.

	Joulaei et al 2013 [11]; Evans et al 2015 [12]	-	National	MOE and MOH; Provincial governors and Parent-Teacher Association	Elementary schools of deprived areas to cover all poor students	- Cover all poor students.	**National Free Food Program (NFFP):**<br>- NFFP supplies 140 kcal and 2.5 g of protein/day. Hence, nutritional value of the NFFP is lower than the scientific recommended snacks for this age group.
	Omidvar et al 2021 [13]	2007	Regional; rural	Ministry of Welfare and Social Security, MOHME and Welfare Organization	Children in rural kindergartens (2–5);<br>Children aged 3–6 years of age in low-income families in rural areas	- Improve the nutritional status of children aged 3–6 years of age in low-income families in rural areas.<br>- Provide part of the children's daily nutritional needs, increase their nutrition awareness as well as that of teachers and kindergarten managers.<br>- Improve the healthy eating habits and behaviors.	Feeding program—providing one warm meal in rural kindergartens. The meal's dietary composition is adapted from the national desirable food basket (395 ± 50 kcal and 19.4 ± 4 g of protein).
		Late 1990s	Regional	Welfare Organization; MOHME; MOE	Primary schools in deprived areas only		**National School Free Snack Program:**<br>Provide packages of food to the targeted schools.
**Iraq**	WHO 2018 [6]	2010	National	MOH and MOE	Kindergartens and schools	- Reduce or prevent child undernutrition (stunting, wasting, micronutrient deficiencies).<br>- Reduce or prevent childhood overweight or obesity.<br>- Foster healthy diet and lifestyle habits.<br>- Educate children and improve knowledge about healthy diet and lifestyle habits.<br>-Improve children's skills (e.g. cooking, food hygiene).<br>- Improve school enrolment.	- Menus are decided according to minimum levels of specific nutrients, following national dietary guidelines.<br>- At the regional or national level, menus are decided by a nutritionist. At the local or municipal level, menus are decided by a health worker.

						- Improve school attendance.<br>- Improve academic performance.<br>- Tackle health inequalities.<br>- Reduce food insecurity and hunger.<br>- Support the agriculture sector by creating farm to school linkages (e.g. cereals, milk, fruit and vegetables supply).	
	WHO 2013 [5]	-	National	-	Kindergartens and schools (primary schools)	-	School feeding programme; provision of school meals based on national dietary guidelines.
**Jordan**	Al-Jawaldeh et al 2020 [1]	-	-	-	Schools	-Prevent undernutrition and overweight or obesity.	Provide free school meals for all children.
	WHO 2018 [6]	1999	Regional	MOE and MOH	Kindergartens and schools	- Reduce or prevent child undernutrition (stunting, wasting, micronutrient deficiencies).<br>- Improve academic performance.<br>- Reduce food insecurity and hunger.	School meals are free for all children.<br>- Menus are decided according to maximum levels of specific nutrients (e.g. total sugars, total fat, saturated fat, trans-fat, salt/sodium), following national food-based dietary guidelines.<br>- At the regional or national level, menus are decided by a nutritionist or other health worker.
**KSA**	WHO 2018 [6]	-	National	MOE and MOH	Kindergartens and schools	- Reduce or prevent child undernutrition (stunting, wasting, micronutrient deficiencies).<br>- Reduce or prevent childhood overweight or obesity.	- Menus are decided following national food-based dietary guidelines.<br>- At the regional or national level, menus are decided by a nutritionist or other health

						- Foster healthy diet and lifestyle habits.<br>- Educate children and improve knowledge about healthy diet and lifestyle habits.<br>- Improve children's skills (e.g. cooking, food hygiene).<br>- Improve school enrolment.<br>- Improve school attendance.<br>- Improve academic performance.	worker. At the local or municipal level, menus are decided by a nutritionist.
	Musaiger et al 2011 [4]	-	-	-	Schools	- Preventative measures for obesity.<br>- Promote healthy eating among school children.	School feeding programme.
**Kuwait**	Musaiger et al 2011 [4]	-	-	-	Schools	- Preventative measures for obesity.<br>- Promote healthy eating among school children.	School feeding programme. Provide free school meals for all children.
	WHO 2018 [6]	1960	National	MOE and MOH	Kindergartens and schools	- Reduce or prevent child undernutrition (stunting, wasting, micronutrient deficiencies).<br>- Reduce or prevent childhood overweight or obesity.<br>- Foster healthy diet and lifestyle habits.<br>- Educate children and improve knowledge about healthy diet and lifestyle habits.<br>- Improve academic performance.	School meals are free for all children.<br>- Menus are decided according to maximum levels of specific nutrients (e.g. total sugars, total fat, saturated fat, trans-fat, salt/sodium), according to minimum levels of specific nutrients (e.g. certain vitamins and minerals).<br>- At the regional or national level, menus are decided by a nutritionist.
**Lebanon**	Jamaluddine et al 2022 [14]	2016 implemented	National	WFP – in coordination	Lebanese and Syrian refugee	- Address food security.	**An emergency school feeding programme (SFP):**

				with concerned governmental entities	children attending primary public schools (Grades 1–6) in the most vulnerable communities across Lebanon		Provides a daily healthy snack pack (fruit, nuts or dairy product).
**Morocco**	Al-Jawaldeh et al 2020 [1]; WHO 2018 [6]	-	National	MOE and MOH in addition to WHO, UNICEF and WFP	Kindergartens and schools	-Prevent undernutrition and overweight or obesity.<br>- Foster healthy diet and lifestyle habits.<br>- Educate children and improve knowledge about healthy diet and lifestyle habits.<br>- Improve children's skills (e.g. cooking, food hygiene).<br>- Improve school enrolment.<br>- Improve school attendance.<br>- Improve academic performance.<br>- Tackle health inequalities.	School meals are free for all children.<br>- Menus are decided following national food-based dietary guidelines.<br>- At the local or municipal level, menus are decided by a health worker. At the school level, menus are decided by a health worker.
	Hanel et al 2021 [15]; WFP 2016 [16]	Implemented (2013)<br><br>The Plan of Action for the enhancement of the National School Meals Programme, which was developed	National (70% in rural areas)	WFP in collaboration with the government (Ministry of National Education and the Ministry of Agriculture); National School Meals Steering Committee	Primary schools	- Improve access to education, nutrition and health through nutritious school meals, in line with a home-grown school meals approach.	The programme provides meals for 1.2 million schoolchildren in 11,500 primary schools, of which 70 percent are located in rural areas.<br><br>WFP and the Moroccan Government jointly developed a Plan of Action for the enhancement of the National School Meals Programme (2016-2018) in alignment with the first pillar of the Strategic Vision 2015-

		with WFP´s assistance and validated by the Moroccan Government in 2016					2030, which aims to ensure equality of opportunity and combat school dropout and repetition rates.
**Oman**	WHO 2013 [17]; Aldinger and Whitman 2009 [18]	2004 – 2009 (for four academic years)	Regional	WHO global initiative, with representation from MOH and MOE	All grades; implemented in 19 schools	- Create a healthier environment and lifestyle in schools and in society.<br>- Address many challenges, especially the unhealthy lifestyle emerging among school students.<br>**-** Raise health awareness of the students and their families, by provision of adequate knowledge of good healthy habits.<br>- Provide comprehensive health services that deal with the physical, mental, and social health needs and problems of this population.<br>- Ensure healthy school environments.	**HPS Initiative:**<br>- Free healthy food to underprivileged children with the support of private sector.
	WHO 2018 [6]; WHO 2013 [5]; Musaiger et al 2011 [4]	1996	National	MOE and MOH, in collaboration with schools and regional muncipalities, schools' adminstrators,	Kindergartens and schools (primary and secondary schools)	- Reduce or prevent child undernutrition (stunting, wasting, micronutrient deficiencies).<br>- Reduce or prevent childhood overweight or obesity.	School feeding programme; provision of school meals based on national dietary guidelines.

				school health teams		- Foster healthy diet and lifestyle habits.<br>- Educate children and improve knowledge about healthy diet and lifestyle habits.<br>- Improve school attendance.<br>- Improve academic performance.	
**Pakistan**	Khan and Ajmal 2011 [19]	2005-2008	National (in 29 districts)	Initiated by the Federal Ministry of Women and Development<br><br>Supported by multilevel collaboration between the Aga Khan University, 11 local NGOs, district governments, Pakistan Baitul Maal and the Federal Ministry	4035 government primary girls' schools	- Reduce malnutrition and increase school enrolment among primary school girls.	The project Tawana provided freshly prepared meals in 4035 government primary girls' schools over a two-year period.<br><br>After three years of operation, the project was replaced by one that contracted with the commercial sector to provide milk and biscuits to the schools instead of the food freshly prepared by the village women.
	Badruddin et al 2008 [20]	Implemented (Pilot)<br><br>2002-2005	National	Funded by the Government of Pakistan<br><br>Aga Khan University partnered the government, 11 NGO's	4035 rural government primary girls' schools	- Combat malnutrition and increase school enrolment among primary school girls.	**Tawana Pakistan Project (TPP):** The project provided freshly prepared balanced noon meals from locally available foods, to over 418 thousand girls.

				facilitated implementation			
**Qatar**	Musaiger et al 2011 [4]	-	-	-	Schools	- Preventative measures for obesity.<br>- Promote healthy eating among school children.	School feeding programme.
**Somalia**	WHO GINA [21]	2014-2016	National	Government of Somalia	Schools	Improve micronutrient status among children in Somalia.	**Somali National Micronutrient Deficiency Control Strategy 2014-2016:**<br>- Advocate for extended distribution of fortified foods in schools, as part of feeding programs.
	WHO GINA [22]	2011-2013	National	Health authorities of Somalia	Schools	Contribute to improved survival and development of Somali people through enhanced nutritional status.	**Somali Nutrition Strategy 2011 – 2013:**<br>- Provide school children with fortified foods in meals at schools.
**Sudan**	WHO GINA [23, 24]	2008-2012	National	MOH in collaboration with the Child and Adolescent Health Directorate, Ministry of Agriculture and Forestry, MOE, School Gardening and Nutrition Education Department and WFP	Schools	- Ensure the prevention and treatment of nutrition related disorders in emergency and non-emergency situations.<br>- Reduce nutritional risk for individuals throughout their life-cycle.<br>- Reduce nutrition risk and improve malnutrition prevention and treatment programming.<br>- Increased knowledge and awareness and improved nutrition practice at community level.<br>- Multi-sectoral coordination and collaboration to address malnutrition comprehensively	**National Nutrition Policy and Key Strategies 2009 and 2008-2012:**<br>- Define the approach and guidelines for school feeding to ensure that where school feeding takes place, this process supports local capacity and does not undermine educational goals.

						and effectively, to bring about sustained change in population nutrition status.	
**Tunisia**	Al-Jawaldeh et al 2020 [1]	-	-	-	Schools	-Prevent undernutrition and overweight or obesity.	Provide free school meals for all disadvantaged children.
	WHO 2018 [6]; WHO 2013 [5]	-	National	-	Kindergartens, primary and secondary schools	-	School feeding programme;<br>- Provision of school meals based on national dietary guidelines.<br>- School meals are free for some children.<br>- Menus are decided selecting menus based on lists of foods and beverages.
**UAE**	Abdullatif et al 2022 [25]; Ahmed 2011 [26]; Khaleej Times 2017 [27]	2011 and in 2017 an update of this policy was released	Regional; Dubai	Government (DHA)	School canteens	Improve health to prevent overweight and obesity among students.	School meals will be inspected for nutritional value and schools will be expected to make pupils and parents aware of the ingredients.
	Musaiger et al 2011 [4]	-	-	-	Schools	- Preventative measures for obesity.<br>- Promote healthy eating among school children.	School feeding programme.
**Take-home rations distributed through schools**							
**Egypt**	WHO 2018 [6]	-	National	MOH and MOE	Kindergartens and schools	Prevent acute malnutrition.	Take-home rations distributed (rice, dates).
**Iraq**	WHO 2013 [5]	-	National	-	Kindergartens, primary and secondary schools	Prevent acute malnutrition.	Take-home rations distributed.
**Yemen**	WHO 2018 [6]	-	National	-	Kindergartens and schools	Prevent acute malnutrition.	Take-home rations distributed.
	**Micronutrient supplementation (e.g. iron, vitamin A)**						

**Afghanistan**	Hees and Sankei 2013 [2]	2009-2010	Regional (8 provinces)	WFP's Afghanistan Protracted Relief Operation (under Food and Education) – in coordination with concerned governmental entities	Students in grades one through nine. 54 schools in 15 food-insecure districts across eight provinces, targeting 46,000 children	- Increase access to education for girls and boys in WFP-assisted schools.<br>- Address micronutrient deficiencies through the provision of food fortified with MNP among 46,000 students in grades one through nine.	Provision of food fortified with MNPs. Micronutrients are provided through school meals using various strategies including processed fortified commodities such as biscuits or fortified blended foods, or by having school cooks add multi-serving packages of MNPs to school meals.<br><br>At school, each student consumes a food basket including fortified wheat flour, pulses, vegetable oil, iodized salt, and MNP.
**Bahrain**	WHO 2013 [5]	2009-2010	National	MOH	Kindergartens, primary and secondary schools	-	Iron and folic acid supplements distributed.
**Iran**	Zandieh et al 2022 [28]; Zandieh et al 2021 [29]	2014<br><br>Total duration (9 months)	National	MOH	High school students	- Reduce the CVD developments in adulthood through correcting adolescents' serum concentration of vitamin D.	In the national vitamin D supplementation program, one pearl of 50,000 IU vitamin D per month was considered for each student for nine months (during autumn, winter, and spring).<br>- Overall, 78.5% of the total population, 1,185,211 students, received vitamin D supplements.
	Banayejeddi et al 2019 [30]	2001<br><br>Total duration (16 weeks)	National	MOH and MOHME	Female students at senior high schools	- Increase the Fe intake among students by delivering free weekly Fe supplements.	**National health promotion program through iron supplementation:**<br>- Prepare educational materials and deliver Fe supplements at the beginning of each school year.

	WHO 2018 [6]	2007	National	MOE and MOH	Kindergartens and schools	- Reduce or prevent child undernutrition (stunting, wasting, micronutrient deficiencies).<br>- Foster healthy diet and lifestyle habits.<br>- Educate children and improve knowledge about healthy diet and lifestyle habits.<br>- Tackle health inequalities.<br>- Reduce food insecurity and hunger.	Micronutrient supplementation in schools.
	WHO 2013 [5]	-	National	MOH	Kindergartens and schools	-	Iron and folic acid supplements distributed.
**Iraq**	WHO 2013 [5]	-	National	-	Kindergartens and schools (primary schools)	-	Vitamin A supplements distributed.
**Jordan**	WHO GINA [31]	2006	National	National Nutrition taskforce, in collaboration with WHO and UNICEF	Planned to cover 550,000 school children and kindergarten to grade 6 (Infants, toddlers and adolescents)	- Reduce the prevalence and burden of diet-related diseases.<br>- Control of nutritional disorders including micronutrient deficiencies.<br>- Control of communicable diseases and NCDs.<br>- Balance of food intake and physical exercises.	Vitamins A, B1, B2, B3, B6, B12 were added to biscuits provided in the school lunch meals served to children.
**Pakistan**	WHO EMRO [32]	2020	National	Government of Pakistan	Schools	- Aim to address micronutrient malnutrition among Pakistani adolescents.	**Adolescent Nutrition Supplementation Guidelines:** Recommends daily iron and folic acid supplementation, provision of multiple micronutrient tablets to underweight non-pregnant women and girls and antenatal counselling on healthy diet.

	WHO GINA [33]	2014	Regional; Balochistan	Government - Directorate of Health Education Department	Schools	Improve human development through enhanced nutritional status of children in Balochistan.	- Iron/folate supplement provision for adolescent girls.
	WHO GINA [34]	2014	Regional; Khyber Pakhtunk	Government - Department of Elementary and Secondary School Education	Schools	- Improve population nutrition wellbeing.<br>- Focus on remedial measures for addressing nutritional issues that have not only been adversely affecting the behavioral, cognitive, scholastic, physical performances but have also been increasing morbidity and mortality and impairing socioeconomic development.	- School milk/food/fortified food and vitamin supplement programme for malnourished children especially middle/high school girls (adolescents).
**Palestine**	Bajraktarevic et al 2021 [35]; WHO 2021[36]	2018	National	UNICEF-supported intervention; supporting MOE and MOH	Schools	- Establish healthy dietary and physical activity habits and improve the nutritional status of school-age children.<br>- Strengthen the involvement of parents, families and communities, complementing formal ongoing school interventions and creating an enabling environment for sustainable positive change around nutrition and healthy lifestyles.<br>- Prevent anaemia and iron deficiency in areas where anaemia is a public health problem.	**Nutrition Friendly Schools Initiative:**<br>Provide micronutrient supplements through the MOH for anaemic children (referred from<br>schools to the primary health care clinics).

**Somalia**	WHO GINA [21]	2014-2016	National	Government of Somalia	Schools	Improve micronutrient status among children in Somalia.	**Somali National Micronutrient Deficiency Control Strategy 2014-2016:**<br>- Advocate for the completion of a school health policy in all zones which will highlight the need for integration of school programs with micronutrient strategies.
**Sudan**	WHO GINA [23, 24]	2009-2012	National	MOH in collaboration with the Child and Adolescent Health Directorate, Ministry of Agriculture and Forestry, MOE, School Gardening and Nutrition Education Department and WFP	School-aged girls	- Ensure the prevention and treatment of nutrition related disorders in emergency and non-emergency situations.<br>- Reduce nutritional risk for individuals throughout their life-cycle.<br>- Reduce nutrition risk and improve malnutrition prevention and treatment programming.<br>- Increased knowledge and awareness and improved nutrition practice at community level.<br>- Multi-sectoral coordination and collaboration to address malnutrition comprehensively and effectively, to bring about sustained change in population nutrition status.	**National Nutrition Policy and Key Strategies 2009 and 2008-2012:**<br>- Explore efficacy and effectiveness of iron/folate supplementation for school age girls to address maternal mortality and improve birth outcomes.

Abbreviations: CVD: cardiovascular diseases; DHA: Dubai Health Authority; EMR: Eastern Mediterranean Region; EMRO: Regional Office for the Eastern Mediterranean; GINA: Global Database on the Implementation of Nutrition Action; HPS: health promoting schools; IU: international units; KSA: Kingdom of Saudi Arabia; MNPs: micronutrient powders; MOE: Ministry of Education; MOH: Ministry of Health; MOHME: Ministry of Health and Medical Education; NCD: non-communicable diseases; NFFP: National Free Food Program; NFP: nutrition focal points; NGO: non-governmental organizations; SFP: school feeding programme; TPP: Tawana Pakistan Project; UAE: United Arab Emirates; UNICEF: United Nations International Children's Emergency Fund; WFP: World Food Programme; WHO: World Health Organization.

References

1. Al-Jawaldeh, A., et al., *Implementation of who recommended policies and interventions on healthy diet in the countries of the eastern mediterranean region: From policy to action.* Nutrients, 2020. **12**(12): p. 1-19.
2. Hees, J.v. and K. Sankei, *Home fortification in school feeding*. 2013, Sight and Life: Basel. p. 39-41.
3. Garemo, M., A. Elamin, and A. Van De Venter, *A review of the nutritional guidelines for children at nurseries and schools in Middle Eastern countries.* Med J Nutrition Metab, 2019. **12**(3): p. 255-270.
4. Musaiger, A.O., A.S. Hassan, and O. Obeid, *The Paradox of Nutrition-Related Diseases in the Arab Countries: The Need for Action.* INTERNATIONAL JOURNAL OF ENVIRONMENTAL RESEARCH AND PUBLIC HEALTH, 2011. **8**(9): p. 3637-3671.
5. World Health Organization, *Global nutrition policy review: what does it take to scale up nutrition action?* 2013, World Health Organization: Geneva, Switzerland.
6. World Health Organization, *Global nutrition policy review 2016–2017: Country progress in creating enabling policy environments for promoting healthy diets and nutrition*. 2018, World Health Organization: Geneva, Switzerland.
7. Ministry of Education and Technical Education-Egypt. *The Ministers of "Health" and "Education" are discussing ways of cooperation to provide healthy meals for school students*. 2021 16 February 2023]; Available from: https://moe.gov.eg/en/what-s-on/news/the-ministers-of-health-and-education/.
8. World Food Programme, *WFP Egypt Country Brief, November 2022*. 2022.
9. Metwally, A.M., et al., *Impact of National Egyptian school feeding program on growth, development, and school achievement of school children.* World J Pediatr, 2020. **16**(4): p. 393-400.
10. Salah, E.M., et al., *The impact of school snacks on cognitive function of primary school children in Egypt.* J Appl Sci Res, 2012. **8**(12): p. 5639-5650.
11. Joulaei, H., et al., *To assess the effects of nutritional intervention based on advocacy approach on malnutrition status among school-aged children in Shiraz.* Journal of Research in Medical Sciences, 2013. **18**(9): p. 739-745.
12. Evans, C.E.L., et al., *School-Based Interventions to Reduce Obesity Risk in Children in High- and Middle-Income Countries.* Advances in Food and Nutrition Research, 2015. **76**: p. 29-77.
13. Omidvar, N., et al., *Enabling food environment in kindergartens and schools in iran for promoting healthy diet: Is it on the right track?* International Journal of Environmental Research and Public Health, 2021. **18**(8).
14. Jamaluddine, Z., et al., *Does a school snack make a difference? An evaluation of the World Food Programme emergency school feeding programme in Lebanon among Lebanese and Syrian refugee children.* Public Health Nutrition, 2022. **25**(6): p. 1678-1690.
15. Hanel, T., et al., *Farm-to-school nutrition programs with special reference to Egypt and Morocco.* The North African Journal of Food and Nutrition Research, 2021. **5**(12): p. 100-104.
16. World Food Programme, *Capacity Development and Support for the National School Feeding Programme. Standard Project Report*. 2016.
17. World Health Organization, *Health-promoting schools initiative in Oman. A WHO case study in intersectoral action*. 2013, World Health Organization: Cairo, Egypt.
18. Aldinger, C. and C.V. Whitman, *Case studies in global school health promotion: from research to practice*. 2009, New York, US: Springer.

19. Khan, K.S. and A. Ajmal, *Women's empowerment and its challenges: review of a multi-partner national project to reduce malnutrition in rural girls in Pakistan*. 2011, World Health Organization: Geneva. p. 117-128.
20. Badruddin, S.H., et al., *Tawana project-school nutrition program in Pakistan - Its success, bottlenecks and lessons learned.* Asia Pacific Journal of Clinical Nutrition, 2008. **17**(SUPPL. 1): p. 357-360.
21. Government of Somalia, *Somali National Micronutrient Deficiency Control Strategy 2014-2016*. 2014.
22. World Health Organization; UNICEF; WFP; FAO; FSNAU, *Somali Nutrition Strategy 2011 – 2013*. 2010.
23. Federal Ministry of Health-Republic of Sudan, *National Nutrition Policy and Key Strategies*. 2009, Maternal and Child Health Directorate.
24. Federal Ministry of Health-Republic of Sudan, *National Nutrition Strategy & Key Strategies (2008 - 2012)*. 2008, Maternal and Child Health Directorate.
25. Abdullatif, M., et al., *Prevalence of Overweight, Obesity, and Dietary Behaviors among Adolescents in Dubai Schools: A Complex Design Survey 2019.* Dubai Med J, 2022.
26. Ahmed, A. *Junk food banned from every Dubai school canteen, The National [Internet]*. 2011 1 June 2023]; Available from: https://www.thenationalnews.com/uae/junk-food-banned-from-every-dubai-school-canteen-1.365770.
27. Khaleej Times. *Food items banned in Dubai school canteens [Internet]*. 2017 1 June 2023]; Available from: https://www.khaleejtimes.com/news/uae-health/11-food-items-banned-in-dubai-school-canteens?_refresh=true.
28. Zandieh, N., et al., *Economic evaluation of a national vitamin D supplementation program among Iranian adolescents for the prevention of adulthood type 2 diabetes mellitus.* BMC Complementary Medicine and Therapies, 2022. **22**(1).
29. Zandieh, N., et al., *The cost-effectiveness analysis of a nationwide vitamin D supplementation program among Iranian adolescents for adulthood cardiovascular diseases prevention.* Public Health, 2021. **198**: p. 340-347.
30. Banayejeddi, M., et al., *Implementation evaluation of an iron supplementation programme in high-school students: The crosswise model.* Public Health Nutrition, 2019. **22**(14): p. 2635-2642.
31. Ministry of Health-Jordan; WHO, *Nutrition in Jordan Update and plan of Action*. 2006.
32. World Health Organization Regional Office for the Eastern Mediterranean. *Nutrition: Success stories*. 27 January 2023]; Available from: http://www.emro.who.int/nutrition/resources/success-stories.html.
33. Government of Balochistan, *Balochistan –An Inter-Sectoral Nutrition Strategy*. 2014, Planning and Development Department.
34. Government of Khyber Pakhtunkhwa, *Khyber Pakhtunkhwa Multi-sectoral Integrated Nutrition Strategy*. 2014, Planning and Development Department.
35. Bajraktarevic, S., et al., *Improving the nutritional well-being of school-age children through the nutrition-friendly schools initiative (NFSI) in the State of Palestine.* Field Exchange - Emergency Nutrition Network ENN, 2021(66): p. 47-50.
36. World Health Organization, *Nutrition action in schools: a review of evidence related to the nutrition-friendly schools initiative*. 2021, World Health Organization: Geneva, Switzerland.
