# Supplementary material for: School-Based Nutrition Programs in the Eastern Mediterranean Region: A Systematic Review
Source: Int J Environ Res Public Health. 2023 Nov 10;20(22):7047. doi: 10.3390/ijerph20227047 (PMC10671197; doi:10.3390/ijerph20227047)
Supplement: Supplementary file 1 [file ijerph-20-07047-s001.zip › Table S3.pdf]

Table S3. Nutrition Education Programs in School Curricula in Countries of the EMR

Country	Reference	Year and Status	National or Regional	Leadership	Target Population	Objective	Brief Description of the Policy/Intervention
**Nutrition education included in school curriculum**							
**Afghanistan**	WHO GINA [1]	2015-2020	National	MOPH	Elementary and secondary school students	To reduce nutrition related mortality and morbidity and contribute to economic development of the nation through reduction in all forms of malnutrition particularly stunting, micronutrients deficiency and acute malnutrition.	Develop nutrition topics for elementary and secondary school teachers to incorporate into subject-specific curricula.
	WHO GINA [2]	2009-2013	National	MOPH	Schools	Increase the awareness about nutrition among the general population, and provide caregivers with the knowledge, skills and support required to adopt healthy nutrition practices, using food-based approaches.	- Integrate nutrition education messages as part of school curricula.<br>- Work with the relevant MOE Departments and education programmes, review school curricula.<br>- Develop appropriate learning aids to be used by school students.
**Bahrain**	WHO 2018 [3]	-	National	MOE and MOH in collaboration with the WHO, UNICEF	Kindergartens, primary and secondary schools	Prevent overweight and obesity.	Nutrition education curriculum includes:<br>- Lessons on healthy diet.<br>- Lessons on the links between nutrition and health.
**Egypt**	WHO 2018 [3]	-	National	MOE and MOH	Kindergartens, primary and secondary schools	- Reduce or prevent child undernutrition (stunting, wasting, micronutrient deficiencies).<br>- Reduce or prevent childhood overweight or obesity.	Nutrition education curriculum includes:<br>- Lessons on healthy diet to prevent undernutrition.

						- Foster healthy diet and lifestyle habits.<br>- Educate children and improve knowledge about healthy diet and lifestyle habits.<br>- Improve children's skills (e.g. cooking, food hygiene).<br>- Improve school enrolment.<br>- Improve school attendance.<br>- Improve academic performance.<br>- Tackle health inequalities.<br>- Reduce food insecurity and hunger.	- Lessons on healthy diet to prevent overweight and obesity.<br>- Lessons on the links between nutrition and health; hands-on cooking skills.
**Iran**	WHO 2018 [3]	2007	National	MOE and MOH	Kindergartens and schools	- Reduce or prevent child undernutrition (stunting, wasting, micronutrient deficiencies).<br>- Reduce or prevent childhood overweight or obesity.<br>- Foster healthy diet and lifestyle habits.<br>- Educate children and improve knowledge about healthy diet and lifestyle habits.<br>- Tackle health inequalities.<br>- Reduce food insecurity and hunger.	Nutrition education included in school curricula.
	Sartipizadeh et al 2021 [4]; Yazdi-Feyzabadi et al 2018 [5]	2010 Piloted<br><br>Total duration (1 year)	5 provinces	MOE and MOHME	Schools	- Promote a healthy school food environment (compliance with healthy food canteen bylaw) combined with health education and improved eating behaviors in adolescents.<br>- Develop concepts of self-care and health promotion in both individual	**Iranian health promoting schools (IHPSs) program:**<br>**-** Teach the healthy nutrition at school.<br>- Comprehensive health education.

						and society or community aspects and as an integrated and coordinated system for school health programs.<br>**-** Improve high-risk behaviours of adolescents.	
	Omidvar et al 2021 [6]	2011	National	MOE and MOHME	Students aged 7-18 years	Foster the healthy development of the whole school community.	**HPS program:**<br>• Provide a framework for developing health promotion initiatives in a way that supports and enhances the implementation of the curriculum.<br>• Support the planning, implementation, and evaluation of health-related activities under the school development planning process.
**Jordan**	Al-Sikheid and Mistareehi 2022 [7]	2004	National	A national team specializing in early childhood, under the supervision of the National Committee for the Development of Pre-School Education, and approved by the	Governmental kindergartens	**-**	**National interactive curriculum**<br>Children-oriented modules for the transfer of food and nutrition knowledge and skills:<br>- Curriculum was adopted to teach kindergarten children, and it began to be applied experimentally as of the beginning of the second semester of the academic year (2003/2004).<br>- Curriculum for teaching healthy eating habits that are culturally accepted, the components of a healthy meal, and meals of various elements, such as: vegetables, fruits, poultry, fish, participating in

				Education Council.			preparing the food and drinks in cooperation with their friends and their teachers, knowing the harms of consuming colored juices and soft drinks.<br>- The curriculum presents activities presented in dialogue and discussion about the importance of integrated nutritional meals.
	WHO GINA [8]	2006<br><br>Total duration (9 months)	National	MOH and WHO	Infants, toddlers and adolescents	- Reduce the prevalence and burden of diet-related diseases.<br>- Control of nutritional disorders including micronutrient deficiencies.<br>- Control of communicable diseases and NCDs.<br>- Balance of food intake and physical exercises.	**Nutrition in Jordan Update and plan of Action:**<br>- Introduce nutrition education in school curricula starting at an early age (primary school).
	WHO 2018 [3]	1999	National	MOE and MOH	Kindergartens and schools	- Reduce or prevent child undernutrition (stunting, wasting, micronutrient deficiencies).<br>- Improve academic performance.<br>- Reduce food insecurity and hunger.	Nutrition education curriculum includes:<br>- Lessons on healthy diet to prevent undernutrition.<br>- Lessons on healthy diet to prevent overweight and obesity.<br>- Lessons on the links between nutrition and health.<br>- Hands-on cooking skills.<br>- Hands-on gardening skills.
**KSA**	WHO 2018 [3]	-	National	MOE and MOH	Kindergartens and schools	- Reduce or prevent child undernutrition (stunting, wasting, micronutrient deficiencies).<br>- Reduce or prevent childhood overweight or obesity.	Nutrition education curriculum includes:<br>- Lessons on healthy diet to prevent undernutrition.<br>- Lessons on healthy diet to prevent overweight and obesity.

						- Foster healthy diet and lifestyle habits.<br>- Educate children and improve knowledge about healthy diet and lifestyle habits.<br>- Improve children's skills (e.g. cooking, food hygiene).<br>- Improve school enrolment.<br>- Improve school attendance.<br>- Improve academic performance.	- Lessons on the links between nutrition and health.
**Kuwait**	WHO 2018 [3]	-	National	MOE and MOH	Kindergartens and schools	- Reduce or prevent child undernutrition (stunting, wasting, micronutrient deficiencies).<br>- Reduce or prevent childhood overweight or obesity.<br>- Foster healthy diet and lifestyle habits.<br>- Educate children and improve knowledge about healthy diet and lifestyle habits.<br>- Improve academic performance.	Nutrition education curriculum includes:<br>- Lessons on the links between nutrition and health.
	Behbehani 2014 [9]; Evans et al 2015 [10]	2013-2017	National	MOE; MOH; Private School Council; Ministry of Commerce; Kuwait Counsellor Network; Ministry of Labour and Social Affairs; Council of	Schools	- Increase the prevalence of sustained PA among the population by 20% and reduce.<br>- Reduce mean BMI significantly by 2% among overweight and obese children, youths and adults.<br>- Reduce mean waist-hip ratio significantly by 5% among overweight and obese children, youths and adults.<br>- Reduce the prevalence of overweight adults in the population by 10%.	**The Kuwait National Programme for Healthy Living:**<br>Introduce knowledge on the pathophysiology of obesity into the school curriculum.

				Religious Affairs		- Reduce the prevalence of overweight children and youths aged 6–18 years in the population by 15%.<br>- Reduce the mean energy intake among the population by 10%.	
**Lebanon**	WHO 2018 [3]	1980	National	MOEHE and MOPH, in addition to WHO, UNICEF, local NGOs	Kindergartens and schools	- Reduce or prevent childhood overweight or obesity.<br>- Foster healthy diet and lifestyle habits.<br>- Educate children and improve knowledge about healthy diet and lifestyle habits.<br>- Tackle health inequalities.	Nutrition education curriculum includes:<br>- Lessons on healthy diet to prevent overweight and obesity.
	MOEHE [11]	2009	National	MOEHE	Schools	- Raise the level of health and environmental awareness in the school community, while enabling students to make decisions.<br>- Improve the health status of children and staff through early detection and follow-up of diseases.	**School Health Strategy:**<br>- Nutrition reinforcement in school curricula.
	El Halabi Ezzeddine and Salameh 2016 [12]	Launched in 1995 by the WHO.<br>In 2010, WHO supported the Lebanese	National	WHO mainly; In 2010, WHO supported the Lebanese MOEHE		Move health education and promotion from being a single and detached activity delivered in classrooms to a setting-based approach that focuses on healthier and supportive environments.	**Health Promoting School Initiative (HPSI)**<br>Comprehensive HPS framework: curriculum, teaching, and learning; school organization, ethos, and environment; and partnerships and services. This framework is

		MOEHE and established a network of 10 HPS					intended to move health education and promotion from being a single and detached health activity delivered in classrooms to a setting-based model that focuses on healthier and supportive environments.<br><br>School health program (SHP) was launched in November 1998 and included health and environmental messages integrated within curricular activities.
**Morocco**	WHO 2018 [3]	-	National	MOE and MOH in addition to WHO, UNICEF and WFP	Kindergartens and schools	- Foster healthy diet and lifestyle habits.<br>- Educate children and improve knowledge about healthy diet and lifestyle habits.<br>- Improve children's skills (e.g. cooking, food hygiene).<br>- Improve school enrolment.<br>- Improve school attendance.<br>- Improve academic performance.<br>- Tackle health inequalities.	Nutrition education curriculum includes:<br>- Lessons on healthy diet to prevent undernutrition.<br>- Lessons on healthy diet to prevent overweight and obesity.<br>- Lessons on the links between nutrition and health.
	Amahmid et al 2020 [13]; Akeef 2015 [14]	Implemented<br><br>The current study was focused on the curricula of the Middle School level,	National	Ministry of National Education	Schools (middle school level)	- Establish healthy eating attitudes and behaviours to improve current and future wellbeing and health of the adolescents.<br>- Spread messages beyond the school impacting families and wider community.	Nutrition lessons given 2 hours a week for 10 weeks throughout the first semester of the academic year.<br><br>Nutrition topics incorporated into the learning program of life and earth sciences.<br><br>Content designed to be delivered using competency-based approach

		established at the national level					and active learning techniques (problem based learning, case studies, practical experiments, questions and answers, visual based learning).
	WHO GINA [15] and Ministry of National Education [16]	2012 although published in 2011<br><br>Total duration 2011-2019	National	MOH	Primary and secondary schools	- Improve the health status of the population by acting on one of its major determinants, nutrition.<br>- Promote a healthy lifestyle to prevent nutritional disorders and nutrition-related chronic diseases.<br>- Strengthen institutional and professional skills in nutrition.	**National Nutrition Strategy 2011-2019:**<br>- Strengthen the nutrition component in the curriculum of primary, secondary schools.
**Oman**	WHO 2018 [3]	1996	National	MOE and MOH	Kindergartens and schools	- Reduce or prevent child undernutrition (stunting, wasting, micronutrient deficiencies).<br>- Reduce or prevent childhood overweight or obesity.<br>- Foster healthy diet and lifestyle habits.<br>- Educate children and improve knowledge about healthy diet and lifestyle habits.<br>- Improve school attendance.<br>- Improve academic performance.	Nutrition education curriculum includes:<br>- Lessons on healthy diet to prevent undernutrition.<br>- Lessons on healthy diet to prevent overweight and obesity.<br>- Lessons on the links between nutrition and health.<br>- Hands-on gardening skills.
**Pakistan**	WHO GINA [17]	2014	Regional; Balochistan	Government - Directorate of Health Education Department	Schools	Improve human development through enhanced nutritional status of children in Balochistan.	- Incorporate nutrition in school curricula.
	WHO GINA [18]	2014	Regional; Khyber Pakhtunk	Government - Department of Elementary and	Schools	- Improve population nutrition wellbeing.<br>- Focus on remedial measures for addressing nutritional issues that	- Changes in school curricula to make it nutrition sensitive.

				Secondary School Education		have not only been adversely affecting the behavioral, cognitive, scholastic, physical performances but have also been increasing morbidity and mortality and impairing socioeconomic development.	
**Palestine**	Bajraktarevic et al 2021 [19]; WHO 2021[20]	2018	National	UNICEF-supported intervention; supporting MOE and MOH	Schools	- Establish healthy dietary and physical activity habits and improve the nutritional status of school-age children.<br>- Strengthen the involvement of parents, families and communities, complementing formal ongoing school interventions and creating an enabling environment for sustainable positive change around nutrition and healthy lifestyles.	**Nutrition Friendly Schools Initiative:**<br>- Nutrition and health-promoting curricula.<br>- Develop and disseminate positive messages on healthy diets and hygiene promotion. This included the development of an interactive game for school-age children, based on topics within the school health curriculum, which included messages on nutritional information and optimal health practices.
**Somalia**	WHO GINA [21]	2014-2016	National	Government of Somalia	Schools	Improve micronutrient status among children in Somalia.	**Somali National Micronutrient Deficiency Control Strategy 2014-2016:**<br>- Integrate short courses into the nutrition education in schools on the causes and prevention of micronutrient malnutrition.
	WHO GINA [22]	2011-2013	National	Health authorities of Somalia	Schools	Contribute to improved survival and development of Somali people through enhanced nutritional status.	**Somali Nutrition Strategy 2011 – 2013:**<br>- Integrate promotion of optimal nutrition and good hygiene practices, education- integrating

							nutrition education into school curricula.<br>*(70% schools provide nutrition education as part of regular curriculum)*
**Sudan**	WHO GINA [23, 24]	2009	National	MOH in collaboration with the Child and Adolescent Health Directorate, Ministry of Agriculture and Forestry, MOE, School Gardening and Nutrition Education Department and WFP	Primary and secondary students	- Ensure the prevention and treatment of nutrition related disorders in emergency and non-emergency situations.<br>- Reduce nutritional risk for individuals throughout their life-cycle.<br>- Reduce nutrition risk and improve malnutrition prevention and treatment programming.<br>- Increased knowledge and awareness and improved nutrition practice at community level.<br>- Multi-sectoral coordination and collaboration to address malnutrition comprehensively and effectively, to bring about sustained change in population nutrition status.	**National Nutrition Policy and Key Strategies 2009 and 2008-2012:**<br>- Incorporate basic nutrition concepts into primary and secondary curricula.
**Tunisia**	Hrairi and Berger 2017 [25]	Implemented<br><br>(3- year intervention period)	Regional	The Chronic Disease Prevention Research Center (CDPRC) with the partnership of different national and	Schools	-	**"Together in Health"**<br>Health education is present throughout the Tunisian curriculum; it includes nutritional education.

				international partners			
	WHO 2018 [3]	-	National	MOE and WFP	Kindergartens and schools	- Reduce or prevent child undernutrition (stunting, wasting, micronutrient deficiencies).<br>- Reduce or prevent childhood overweight or obesity.<br>- Foster healthy diet and lifestyle habits<br>- Educate children and improve knowledge about healthy diet and lifestyle habits.<br>- Improve children's skills (e.g. cooking, food hygiene).<br>- Improve school enrolment.<br>- Improve school attendance.<br>- Improve academic performance.<br>- Tackle health inequalities.<br>- Reduce food insecurity and hunger.	Nutrition education curriculum includes: - Lessons on healthy diet to prevent undernutrition.<br>- Lessons on healthy diet to prevent overweight and obesity.<br>- Lessons on the links between nutrition and health.<br>- Hands-on cooking skills.<br>- Hands-on gardening skills.
**UAE**	MOE 2017 [26]	2017	National	MOE	All government schools; From kindergarten to grade 12	Empower young Emirati students to take ownership of their physical education, health and wellbeing to ensure a future generation of healthy, motivated, highly educated Emiratis.	**Physical and Health Education Curriculum**<br>- Introduce Physical and Health Education as a subject in the national curriculum.<br>- Some of these key areas include health and wellbeing, diet and nutrition.<br>- The curriculum focuses on practical and interactive activities.
**Yemen**	WHO 2018 [3]	-	National	-	Kindergartens and schools	-	Nutrition education included in school curricula.

**Several countries: Lebanon, Jordan, Palestine, Bahrain, KSA and UAE**	Habib-Mourad et al 2022 [27]; Habib et al 2014 [28]; Evans et al 2015 [10]	2010 Lebanon; 2015 Jordan<br><br>(3-4 months duration, each)	National	Public-private partnership: Nestlé Middle East FZE; AUB; MOE; MOH	School teachers of 9-11 year old students	- Tackle childhood obesity by addressing nutritional and physical activity habits of schoolchildren.<br><br>- Promote healthy eating and physical activity habits among 9–11-year-old student.<br><br>- Raise the degree of nutritional and health awareness among students, their families, school health and nutrition officials and teaching staff, and promote positive trends and modify incorrect trends.	Culturally appropriate educational lessons, included fun and attractive material, designed to promote healthy eating delivered by trained teachers to students in all classes.<br><br>Changes to the local educational authorities and health institutions roles and structure in the UAE and KSA prevented the program continuation and affected sustainability; the program continues to be implemented in four out of six countries: Lebanon, Jordan, Palestine, and Bahrain.

Abbreviations: AUB: American University of Beirut; CDPRC: Chronic Disease Prevention Research Center; FZE: free zone establishments; GINA: Global Database on the Implementation of Nutrition Action; HPS: health promoting schools; HPSI: health promoting school initiative; IHPS: Iranian health promoting schools; KSA: Kingdom Saudi Arabia; MOE: Ministry of Education; MOEHE: Ministry of Education and Higher Education; MOH: Ministry of Health; MOHME: Ministry of Health and Medical Education; MOPH: Ministry of Public Health; NCD: non-communicable diseases; NGO: non-governmental organizations; SHP: school health program; UAE: United Arab Emirates; UNICEF: United Nations International Children's Emergency Fund; WHO: World Health Organization; WFP: World Food Programme.

References

1. Ministry of Public Health-Afghanistan. *National Public Nutrition Policy and Strategy 2015-2020*; 2015; Available online: https://extranet.who.int/ncdccs/Data/AFG_B14_Public_Nutrition_Policy_Strategy_2015.pdf.
2. Ministry of Public Health-Afghanistan. *National Public Nutrition Policy and Strategy 2009-2013*; 2009; Available online: https://extranet.who.int/nutrition/gina/en/node/17851.
3. World Health Organization. *Global nutrition policy review 2016–2017: Country progress in creating enabling policy environments for promoting healthy diets and nutrition*; World Health Organization: Geneva, Switzerland, 2018; Available online: https://www.who.int/publications/i/item/9789241514873.
4. Sartipizadeh, M.; Yazdi-Feyzabadi, V.; Alipouri Sakha, M.; Zarrin, A.; Bazyar, M.; Zahirian Moghadam, T.; Zandian, H. Evaluating the Health Promoting Schools in Iran: Across-Sectional Study. *Health Education* **2021,** *121*, 125-139.
5. Yazdi-Feyzabadi, V.; Omidvar, N.; Mohammadi, N. K.; Nedjat, S.; Karimi-Shahanjarini, A.; Rashidian, A. Is an Iranian health promoting school status associated with improving school food environment and snacking behaviors in adolescents? *Health Promotion International* **2018,** *33*, 1010-1021.
6. Omidvar, N.; Babashahi, M.; Abdollahi, Z.; Al-Jawaldeh, A. Enabling food environment in kindergartens and schools in iran for promoting healthy diet: Is it on the right track? *International Journal of Environmental Research and Public Health* **2021,** *18*.
7. Al-Shikheid, W.; Mistareehi, H. The extent of the national interactive curriculum contributes to health awareness concepts development among Public kindergarten children from female teachers' perspective in of Zarqa governorate. *An-Najah National University* **2022,** *36*, 1703-1740.
8. Ministry of Health-Jordan; WHO. *Nutrition in Jordan Update and plan of Action*; 2006; Available online: https://extranet.who.int/nutrition/gina/sites/default/filesstore/aNutrition%20in%20Jordan-Policy.pdf.
9. Behbehani, K. Kuwait national programme for healthy living: First 5-year plan (2013-2017). *Medical Principles and Practice* **2014,** *23*, 32-42.
10. Evans, C. E. L.; Albar, S. A.; Vargas-Garcia, E. J.; Xu, F. School-Based Interventions to Reduce Obesity Risk in Children in High- and Middle-Income Countries. *Advances in Food and Nutrition Research* **2015,** *76*, 29-77.
11. Ministry of Education and Higher Education-Lebanon. *School Health Strategy*; 2009; Available online: https://www.mehe.gov.lb/ar/Projects/%D8%A7%D9%84%D8%AA%D8%B9%D9%84%D9%8A%D9%85%20%D8%A7%D9%84%D8%B9%D8%A7%D9%85/School%20Health%20Strategy.pdf.
12. El Halabi Ezzeddine, S.; Salameh, P. Incorporating an Innovative Health Promoting Model Into Lebanese Public Schools: Impact on Adolescents' Dietary and Physical Activity Practices--Comparison of HPS With Other Public and Private Schools in Lebanon. *Global Journal of Health Education & Promotion* **2016,** *17*, 53-87.
13. Amahmid, O.; El Guamri, Y.; Rakibi, Y.; Yazidi, M.; Razoki, B.; Kaid Rassou, K.; El Boukaoui, S.; Izerg, O.; Belghyti, D. Nutrition education in school curriculum: impact on adolescents' attitudes and dietary behaviours. *International Journal of Health Promotion and Education* **2020,** *58*, 242-258.

14. Akeed, F. Tackling Health Education in the Textbooks of Life and Earth Sciences at the Lower Secondary Education in the Kingdom of Morocco. *Journal of Al-Quds Open University for Educational & Psychological Research & Studies* **2015,** *3*, 271-298.
15. Ministry of Health-Morocco; UNICEF. *La Stratégie Nationale de la Nutrition*; 2011; Available online: https://extranet.who.int/nutrition/gina/en/node/17819.
16. Ministry of National education-Morocco. Nutrition education. Available online: https://www.men.gov.ma/Fr/Pages/Edunutri.aspx (accessed on 20 February 2023).
17. Government of Balochistan. *Balochistan –An Inter-Sectoral Nutrition Strategy*; Planning and Development Department: 2014; Available online: https://extranet.who.int/nutrition/gina/en/node/39754.
18. Government of Khyber Pakhtunkhwa. *Khyber Pakhtunkhwa Multi-sectoral Integrated Nutrition Strategy*; Planning and Development Department: 2014; Available online: https://extranet.who.int/nutrition/gina/sites/default/filesstore/PAK_2014_Khyber%20Pakhtunkhwa%20Integrated%20Nutrition%20Strategy.pdf.
19. Bajraktarevic, S.; Qadi, K.; Jouda, A.; Awadallah, Y.; Abueita, R. Improving the nutritional well-being of school-age children through the nutrition-friendly schools initiative (NFSI) in the State of Palestine. *Field Exchange - Emergency Nutrition Network ENN* **2021,** 47-50.
20. World Health Organization. *Nutrition action in schools: a review of evidence related to the nutrition-friendly schools initiative*; World Health Organization: Geneva, Switzerland, 2021; Available online: https://www.who.int/publications/i/item/9789241516969.
21. Government of Somalia. *Somali National Micronutrient Deficiency Control Strategy 2014-2016*; 2014; Available online: https://extranet.who.int/nutrition/gina/sites/default/filesstore/SOM_MN%20Strategy%20-%202014-2016.pdf.
22. World Health Organization; UNICEF; WFP; FAO; FSNAU. *Somali Nutrition Strategy 2011 – 2013*; 2010; Available online: https://extranet.who.int/nutrition/gina/sites/default/filesstore/SOM%202010%20Nutrition%20Strategy.pdf.
23. Federal Ministry of Health-Republic of Sudan. *National Nutrition Policy and Key Strategies*; Maternal and Child Health Directorate: 2009; Available online: http://www.fmoh.gov.sd/Health-policy/nationalnutritionpolicy.pdf.
24. Federal Ministry of Health-Republic of Sudan. *National Nutrition Strategy & Key Strategies (2008 - 2012)*; Maternal and Child Health Directorate: 2008; Available online: https://extranet.who.int/nutrition/gina/sites/default/filesstore/SDN%202008_National%20Nutrition%20Policy.pdf.
25. Hrairi, S.; Berger, D. Nutrition Education in Tunisian schools: analysis of practices reported by primary teachers. *QUESTIONS VIVES-RECHERCHES EN EDUCATION* **2017**.
26. Ministry of Education-UAE. Ministry of Education announces a brand new Physical and Health Education reform in the UAE. Available online: https://www.moe.gov.ae/En/MediaCenter/News/Pages/sport.aspx (accessed on 16 March 2023).
27. Habib-Mourad, C.; Hwalla, N.; Maliha, C.; Zahr, S.; Antoniades, K. Ajyal Salima a novel public–private partnership model for childhood obesity prevention in the Arab countries. *Front Public Health* **2022,** *10*.
28. Habib-Mourad, C.; Moore, H.; Zeidan, M. N.; Hwalla, N.; Summerbell, C. Health-E-PALS: Promoting healthy eating and physical activity in Lebanese school children - Intervention development. *Educ Health* **2014,** *32*, 3-8.
