# Supplementary material for: School-Based Nutrition Programs in the Eastern Mediterranean Region: A Systematic Review
Source: Int J Environ Res Public Health. 2023 Nov 10;20(22):7047. doi: 10.3390/ijerph20227047 (PMC10671197; doi:10.3390/ijerph20227047)
Supplement: Supplementary file 1 [file ijerph-20-07047-s001.zip › Table S4.pdf]

Table S4. Extracurricular Nutrition Education Initiatives in Countries of the EMR

Country	Reference	Year and Status	National or Regional	Leadership	Target Population	Objective	Brief Description of the Policy/Intervention
**Extracurricular nutrition education**							
**Bahrain**	Public Health Directorate Bahrain 2014 [1]	2014	National	The Public Health Directorate	Schools	Prevent disease and promote health of the population.	Nutritional and health awareness and publications through lectures at schools.
	WHO GINA [2]	2012	National	MOH	School students	- Sustain the population's health through health promotion and prevention:<br>Promote healthy lifestyles to reduce non-communicable diseases.	Develop National Action Plan to encourage healthy lifestyles.<br>- Continue strengthening the health promotion programs at schools.
	Aldinger and Whitman 2009 [3]; AlMulla AlHarmasAlHajeri et al 2009 [4]	2004	Regional; Muharraq governorate	MOH in collaboration with the MOE, GCC and WHO EMRO	Schools	- Provide instruction to develop the knowledge, skills, attitudes, and behaviors related to healthy living.<br>- Support the provision of support services for students and their families.<br>- Create a healthy social and physical environment within the school.<br>- Integrate the concepts of personal health management, health promotion, and education.<br>- Incorporate strategies that are comprehensive, interdisciplinary, and outcome based.<br>- Be taught by teachers who are competent and qualified in health education and promotion.	**Health-Promoting Schools (HPS) program:**<br>**-** All schools had components of health promotion, such as nutrition, and health education, but these components were not coordinated in an organized way.

						- Provide sufficient instruction time to elicit behavior change.	
**Egypt**	Information provided by NFP; Ministry of Education and Technical Education [5]	2019	National	MOH and Ministry of Education and Technical Education	School students	- Improve the healthy nutrition of school students to treat and prevent diseases resulting from malnutrition.<br>- Intensify awareness about healthy, useful eating, and healthy meals appropriate for the appropriate age group.	**100 Million Health Initiative:** Intensify awareness about healthy, useful eating, and healthy meals appropriate for the appropriate age group.
**Iran**	Omidvar et al 2021 [6]	2020	National	MOE and MOHME	Students (5-18 years), teachers and parents	- Change students' behavior and lifestyle to an active and healthy lifestyle.<br>- 3% reduction in the number of obese and overweight students.	**Weight and obesity control in students (Kouch):**<br>- Promote a healthy food environment in school and at home to control students' calorie intake through nutrition **education of students, parents, and teachers.**<br>- After the COVID-19 pandemic, these educational activities have been continued virtually through the Shad application designed by the MOE.
	Omidvar et al 2021 [6]; Sayyari et al 2017 [7]	2016	-	MOE and MOHME	Kindergartens and school canteens (2-18 years), and their environments	- Improve population health and health equity in childhood obesity prevention.<br>- Reduce the incidence of childhood obesity and identify and treat preexisting obesity in children and	**The IRAN-Ending Childhood Obesity program:**<br>- Provide educational materials on health and nutrition.<br>- Provide written educational materials, group learning, family lifestyle modification, and increasing the public

						adolescents.	awareness about childhood obesity.<br>- Hold nutrition education classes, development and distribution of educational materials for mothers in kindergartens and schools.
	Sartipizadeh et al 2021 [8]; Yazdi-Feyzabadi et al 2018 [9]	2010 Piloted	5 provinces	MOE and MOHME	Schools	- Promote a healthy school food environment (compliance with healthy food canteen bylaw) combined with health education and improved eating behaviors in adolescents.<br>- Develop concepts of self-care and health promotion in both individual and society or community aspects and as an integrated and coordinated system for school health programs.<br>- Improve high-risk behaviours of adolescents.	**Iranian health promoting schools (IHPSs) program:**<br>- Parents, students and community participation in health promotion programs.
	WHO 2018 [10]	2007	National	MOE and MOH	Kindergartens and schools	- Reduce or prevent child undernutrition (stunting, wasting, micronutrient deficiencies).<br>- Reduce or prevent childhood overweight or obesity.<br>- Foster healthy diet and lifestyle habits.<br>- Educate children and improve knowledge about healthy diet and lifestyle habits.<br>- Tackle health inequalities.	Extracurricular nutrition education.

						- Reduce food insecurity and hunger.	
	Gupta et al 2012 [11]; Kelishadi et al 2003 [12]; Sarraf-Zadegan et al 2003 [13]	2000-2001 till 2004-2005	-	Isfahan Cardiovascular Research Center and Isfahan Provincial Health Office	Children 2-18 year olds, their parents, teachers, related healthcare providers in schools	- Improve lifestyle habits, knowledge, and behavior toward healthy nutrition and reduce the prevalence of obesity and cardiovascular risk factors.	**Isfahan Healthy Heart Program-Heart Health Promotion from Childhood**
**Iraq**	WHO GINA [14]	2012-2021	National	General Secretariat for the Council of Ministers	School students	- Ensure the health and nutrition status.<br>- Improve the nutritional status of Iraqi people throughout the life cycle from 2012 to 2021.<br>- Reduce prevalence of wasting and stunting among children under five years of age.<br>- Reduce prevalence of overweight and obesity among all age groups.<br>- Provide nutritional health promotion and counseling to people at community level.<br>- Reduce prevalence of micronutrient deficiencies.	National Nutrition Strategy 2012-2021:<br>- Promote education in nutrition.
**Jordan**	WHO GINA [15]	2006	National	MOH and WHO	Infants, toddlers and adolescents	- Reduce the prevalence and burden of diet-related diseases.<br>- Control of nutritional disorders including micronutrient deficiencies.<br>- Control of communicable diseases and NCDs.<br>- Balance of food intake and physical exercises.	**Nutrition in Jordan Update and plan of Action:**<br>- Conduct education health and nutrition programmes in schools.

	MOE [16]	-	National	MOE, Department of School Nutrition and Health; in collaboration with MOH	Schools	-	- Work to raise the degree of nutritional and health awareness among students.<br>- Prepare nutritional and health educational leaflets and disseminating them to schools.<br>- Hold seminars, workshops and meetings related to nutritional and health issues.
	WHO 2018 [10]	1999	National	MOE and MOH	Kindergartens and schools	- Reduce or prevent child undernutrition (stunting, wasting, micronutrient deficiencies).<br>- Improve academic performance.<br>- Reduce food insecurity and hunger.	Extracurricular nutrition education.
**Kuwait**	MOH et al [17]; WHO EMRO [18]	-	National	Led by the FNA on behalf of the Kuwait MOH, in partnership with the WHO and the food industries	Schools	Improve the health among children in Kuwait.	- Raise nutrition and health awareness through continuous education program.
**Lebanon**	Habib-Mourad et al 2022 [19]; Habib et al 2014 [20]; Evans et al 2015 [21]	2010 implemented<br><br>2014: adoption of the program by the MOEHE into	National	Public-private partnership: Nestlé Middle East FZE; AUB; MOEHE	Parents	- Tackle childhood obesity by addressing nutritional and physical activity habits of schoolchildren.<br><br>- Promote healthy eating and physical activity habits among 9–11-year-old student.	**Ajyal Salima**<br>Parental involvement: The intervention program was introduced to families to assist them in creating a supportive environment at home for healthy lifestyle behaviors. Take-home packets including

		their education health unit<br><br>(3-4 months duration)				- Raise the degree of nutritional and health awareness among students, their families, school health and nutrition officials and teaching staff, and promote positive trends and modify incorrect trends.	nutrition tips, as well as recipes were sent with the students. Additionally, parents were invited to health fairs organized at the school. This component covered the obesogenic environment at home.
	WHO 2018 [10]	1980	National	MOEHE and MOPH, in addition to WHO, UNICEF, local NGOs	Kindergartens and schools	- Reduce or prevent childhood overweight or obesity.<br>- Foster healthy diet and lifestyle habits.<br>- Educate children and improve knowledge about healthy diet and lifestyle habits.<br>- Tackle health inequalities.	Extracurricular nutrition education.
	WHO GINA [22]	Policy since but adopted in 2015	National	MOH	Schools	- Reinforce the school health program.	- Strengthen health education activities in schools and promoting healthy lifestyles.<br>- Conduct the global school based student health survey (GSHS).
**Morocco**	WHO 2018 [10]	-	National	MOE and MOH in addition to WHO, UNICEF and WFP	Kindergartens and schools	- Foster healthy diet and lifestyle habits.<br>- Educate children and improve knowledge about healthy diet and lifestyle habits.<br>- Improve children's skills (e.g. cooking, food hygiene).<br>- Improve school enrolment.<br>- Improve school attendance.<br>- Improve academic performance.<br>- Tackle health inequalities.	A nutrition education program is implemented annually through a media campaign at the school level.

**Oman**	WHO 2013 [23]; Aldinger and Whitman 2009 [3]	2004 – 2009 (for four academic years)	Regional	WHO global initiative, with representation from MOH and MOE	All grades; implemented in 19 schools	- Create a healthier environment and lifestyle in schools and in society.<br>- Address many challenges, especially the unhealthy lifestyle emerging among school students.<br>**-** Raise health awareness of the students and their families, by provision of adequate knowledge of good healthy habits.<br>- Provide comprehensive health services that deal with the physical, mental, and social health needs and problems of this population.<br>- Ensure healthy school environments.	**HPS Initiative:**<br>- Health education activities for dietary and food safety measures such as peer education, healthy dish competition between students.<br>- Use interactive nutritional education programs.
**Pakistan**	WHO GINA [24]	2014	Regional; Khyber Pakhtunk	Government - Department of Elementary and Secondary School Education	Schools	- Improve population nutrition wellbeing.<br>- Focus on remedial measures for addressing nutritional issues that have not only been adversely affecting the behavioral, cognitive, scholastic, physical performances but have also been increasing morbidity and mortality and impairing socioeconomic development.	- Introduce school health and nutrition assessment programme.<br>- Establish and strengthen nutrition oriented programmes.<br>- Nutrition advocacy, behavioural modification and awareness.<br>- Organize debates/seminars and workshops on Nutrition and Health.

**Palestine**	Bajraktarevic et al 2021 [25]; WHO 2021[26]	2018	National	UNICEF-supported intervention; supporting MOE and MOH	Parents, families and communities of school-aged children	- Establish healthy dietary and physical activity habits and improve the nutritional status of school-age children.<br>- Strengthen the involvement of parents, families and communities, complementing formal ongoing school interventions and creating an enabling environment for sustainable positive change around nutrition and healthy lifestyles.	**Nutrition Friendly Schools Initiative (NFSI):**<br>- Strengthen the involvement of parents, families and communities, complement formal ongoing school interventions and create an enabling environment for sustainable positive change around nutrition and healthy lifestyles.<br>**-** Parents were also provided with educational and informational material regarding the NFSI programme and the importance of healthy nutrition.
					School-aged children		**Nutrition Friendly Schools Initiative (NFSI):**<br>- Conduct awareness-raising sessions on anaemia and lifestyle modifications.<br>- Develop educational and communication material for community awareness. This included messaging through radio and television channels on healthy lifestyle practices, nutritious foods and diet diversity.<br>- Conduct monthly or weekly group breakfast events in targeted schools.

							- Hold drawing competitions in schools at both the local and national level to identify the 'best' healthy/nutrition-related painting.<br>- Compose and produce theatre plays and songs by children at school open days to promote healthy lifestyles.<br>- Develop school plays for school-aged children in 10 schools that targeted healthy habits and nutrition messages.
	Habib-Mourad et al 2022 [19]	2016		Public-private partnership: Nestlé Middle East FZE; AUB; MOE; MOH	Schools	- Tackle childhood obesity by addressing nutritional and physical activity habits of schoolchildren.<br><br>- Promote healthy eating and physical activity habits among 9–11-year-old student.<br><br>- Raise the degree of nutritional and health awareness among students, their families, school health and nutrition officials and teaching staff, and promote positive trends and modify incorrect trends.	**Ajyal Salima:**<br>The program was integrated into Palestinian strategic education and school health action plans.

	MOEHE [27]	-	National	MOEHE	Schools and their environment	- Provide students with healthy eating habits and behaviours.<br>- Improve the nutritional status in schools.	- Improve students' health and nutritional status.<br>- Improve and develop the school environment.<br>- Provide students with healthy nutritional habits and behaviors.
**Sudan**	WHO GINA [28, 29]	2009-2012	National	MOH in collaboration with the Child and Adolescent Health Directorate, Ministry of Agriculture and Forestry, MOE, School Gardening and Nutrition Education Department and WFP	School-aged children and adolescents; environment and community	- Ensure the prevention and treatment of nutrition related disorders in emergency and non-emergency situations.<br>- Reduce nutritional risk for individuals throughout their life-cycle.<br>- Reduce nutrition risk and improve malnutrition prevention and treatment programming.<br>- Increased knowledge and awareness and improved nutrition practice at community level.<br>- Multi-sectoral coordination and collaboration to address malnutrition comprehensively and effectively, to bring about sustained change in population nutrition status.	**National Nutrition Policy and Key Strategies 2009 and 2008-2012:**<br>- Develop educational materials to promote nutrition of school-aged children and adolescents.<br>- Promote supportive environment and community awareness on adequate nutrition for school-aged and adolescents.
**Tunisia**	WHO GINA [30, 31]	2012 and 2015-2016	National	MOH	Kindergartens and primary schools	Reduce morbidity, disabilities and premature mortality related to NCDs and their risk factors:<br>- Strengthen the promotion of healthy lifestyles and the prevention of NCDs.<br>- Ensure quality management of NCDs	**Obesity Prevention and Control Strategy 2012; National Multisectoral Strategy for the Prevention and Control of Non-Communicable Diseases (NCD) 2018-2025:**

						- Improve governance at all levels of competence and responsibility.<br>- Develop a monitoring and evaluation system for NCDs and their risk factors.	- Practical courses in healthy cooking are organized in the kindergartens and primary schools.
**UAE**	Evans et al 2015 [21]; UAE Moments 2022 [32]; The National News [33]	2010			Government school students and their parents	- Find a solution for the increasing problem of childhood obesity.	**"Smart Canteen"**<br>**-** Educate children about the content of food so as to encourage them to make healthier choices.<br>- Provide the food content and state the energy intake limit for that pupil. For example, if the pupil has AED20 the system will not allow the pupil to buy a burger for AED20, unless the pupil has sufficient calories, carbohydrates, proteins, sugar, sodium, and fat allowance left in his account<br>- Enable parents of government school students to participate virtually with their children in choosing their meals from the school's canteen.

	Gupta et al 2012 [11]; Emirates News Agency 2009 [34]	2009	National	MOH, in partnership with UNICEF and in cooperation with different institutions and stakeholders such as the MOE. The campaign is also supported by UNICEF's strategic partner the General Women's Union, WHO, the MOE and Princess Haya initiative for the Development Health	Schools	- Combat childhood obesity.	**The Fat Truth Campaign, under the slogan "Get Involved So They Can Too"** In this campaign, several school-based activities were implemented, workshops were organized, and an intensive media campaign was launched. - Increase awareness among school students and encourage them to engage in healthy practices, and avoid harmful practices.
	Aldinger and Whitman 2009 [3]	2004	National	MOH	Students; More than 17,000 students have benefited from the project in 46 schools	Promote, protect, and improve the health of students in UAE, through a comprehensive and coordinated school health program.	**HPS project** School health staff provide nutrition education. Their activity complements school nutrition education included in the integrated health curriculum in relevant subjects.

	Algurg et al 2021 [35]; AlGurg et al 2020 [36]	-	Regional; Dubai	Ministry of Health and Prevention	Public schools and corresponding community	Reduce obesity prevalence to 12% by the year 2021.	**Health Awareness Campaigns**<br>Periodic campaigns to boost awareness about healthy eating among students.
				Food safety department, Dubai Municipality	Schools		**Guidelines and requirements for food and nutrition in Dubai**<br>Raise awareness of students about the nutrient quotient in foods.
	Bani-Issa et al 2020 [37]	-	Regional; Abu Dhabi	The regulatory Health Authority of Abu Dhabi	Students, peers, families and school communities	- Teach children the importance of diet and physical activity, and students will, in turn, educate their peers, families, and school communities about good nutrition.	**"Schools for Health":**<br>- Recognize the mutual benefits of education and health.

Abbreviations: AUB: American University of Beirut; EMRO: Regional Office for the Eastern Mediterranean; FNA: Food and Nutrition Authority; FZE: free zone establishments; GCC: Gulf Cooperation Council; GINA: Global Database on the Implementation of Nutrition Action; GSHS: global school based student health survey; HPS: health promoting schools; IHPS: Iranian health promoting schools; MOE: Ministry of Education; MOEHE: Ministry of Education and Higher Education; MOH: Ministry of Health; MOHME: Ministry of Health and Medical Education; MOPH: Ministry of Public Health; NCD: non-communicable diseases;

NFP: nutrition focal points; NFSI: nutrition friendly schools initiative; NGO: non-governmental organizations; UAE: United Arab Emirates; UNICEF: United Nations International Children's Emergency Fund; WFP: World Food Programme; WHO: World Health Organization.

References

1. Public Health Directorate Bahrain. *Annual report*; Public Health Directorate: Manama, Bahrain, 2014; Available online: https://www.moh.gov.bh/Content/Files/Publications/X_635906124344978750.pdf.
2. Ministry of Health-Bahrain. *Bahrain's Health Agenda. Health Improvement Strategy*; Directorate of Health Planning: 2012; Available online: https://extranet.who.int/nutrition/gina/sites/default/filesstore/BHR%202012%20%20Health%20Improvement%20Strategy.pdf.
3. Aldinger, C.; Whitman, C. V. *Case studies in global school health promotion: from research to practice*; Springer: New York, US, 2009.
4. AlMulla AlHarmasAlHajeri, M.; Al Thukair, L. A. A. A.; Sarhan, N. Bahrain: National Comprehensive School Health Program, Health-Promoting Schools. *Case Studies in Global School Health Promotion: From Research to Practice* **2009,** 239-249.
5. Ministry of Education and Technical Education-Egypt. The Ministers of "Health" and "Education" are discussing ways of cooperation to provide healthy meals for school students. Available online: https://moe.gov.eg/en/what-s-on/news/the-ministers-of-health-and-education/ (accessed on 16 February 2023).
6. Omidvar, N.; Babashahi, M.; Abdollahi, Z.; Al-Jawaldeh, A. Enabling food environment in kindergartens and schools in iran for promoting healthy diet: Is it on the right track? *International Journal of Environmental Research and Public Health* **2021,** *18*.
7. Sayyari, A. A.; Abdollahi, Z.; Ziaodini, H.; Olang, B.; Fallah, H.; Salehi, F.; Heidari-Beni, M.; Imanzadeh, F.; Abasalti, Z.; Fozouni, F. et al. Methodology of the comprehensive program on prevention and control of overweight and obesity in iranian children and adolescents: The Iran-ending childhood obesity (Iran-ECHO) program. *International Journal of Preventive Medicine* **2017,** *8*.
8. Sartipizadeh, M.; Yazdi-Feyzabadi, V.; Alipouri Sakha, M.; Zarrin, A.; Bazyar, M.; Zahirian Moghadam, T.; Zandian, H. Evaluating the Health Promoting Schools in Iran: Across-Sectional Study. *Health Education* **2021,** *121*, 125-139.
9. Yazdi-Feyzabadi, V.; Omidvar, N.; Mohammadi, N. K.; Nedjat, S.; Karimi-Shahanjarini, A.; Rashidian, A. Is an Iranian health promoting school status associated with improving school food environment and snacking behaviors in adolescents? *Health Promotion International* **2018,** *33*, 1010-1021.
10. World Health Organization. *Global nutrition policy review 2016–2017: Country progress in creating enabling policy environments for promoting healthy diets and nutrition*; World Health Organization: Geneva, Switzerland, 2018; Available online: https://www.who.int/publications/i/item/9789241514873.
11. Gupta, N.; Goel, K.; Shah, P.; Misra, A. Childhood obesity in developing countries: Epidemiology, determinants, and prevention. *Endocr Rev* **2012,** *33*, 48-70.
12. Kelishadi, R.; Hashemi Pour, M.; Sarraf-Zadegan, N.; Sadry, G. H.; Ansari, R.; Alikhassy, H.; Bashardoust, N. Obesity and associated modifiable environmental factors in Iranian adolescents: Isfahan healthy Heart program - Heart health promotion from childhood. *Pediatrics International* **2003,** *45*, 435-442.

13. Sarraf-Zadegan, N.; Sadri, G.; Malek Afzali, H.; Baghaei, M.; Mohammadi Fard, N.; Shahrokhi, S.; Tolooie, H.; Poormoghaddas, M.; Sadeghi, M.; Tavassoli, A. et al. Isfahan Healthy Heart Programme: A comprehensive integrated community-based programme for cardiovascular disease prevention and control. Design, methods and initial experience. *Acta Cardiol* **2003,** *58*, 309-320.
14. Ministry of Health-Iraq. *National Nutrition Strategy 2012-2021*; 2012; Available online: https://extranet.who.int/nutrition/gina/en/node/8387.
15. Ministry of Health-Jordan; WHO. *Nutrition in Jordan Update and plan of Action*; 2006; Available online: https://extranet.who.int/nutrition/gina/sites/default/filesstore/aNutrition%20in%20Jordan-Policy.pdf.
16. Ministry of Education-Jordan. Department of School Nutrition and Health. Available online: https://moe.gov.jo/node/21657 (accessed on 16 February 2023).
17. Ministry of Health-Kuwait; Gulf Health Council; United Nations Development Programme; World Health Organization; Secretariat of the UN Inter-Agency Task Force on NCDS. *The Case for Investment in Prevention and Control of Non-Communicable Diseases in Kuwait*; Ministry of Health: Kuwait, 2021; Available online:
18. World Health Organization Regional Office for the Eastern Mediterranean. *Kuwait health profile 2015*; World Health Organization Regional Office for the Eastern Mediterranean: 2016; Available online: https://apps.who.int/iris/bitstream/handle/10665/253770/EMROPUB_2016_EN_19271.pdf?sequence=1&isAllowed=y.
19. Habib-Mourad, C.; Hwalla, N.; Maliha, C.; Zahr, S.; Antoniades, K. Ajyal Salima a novel public–private partnership model for childhood obesity prevention in the Arab countries. *Front Public Health* **2022,** *10*.
20. Habib-Mourad, C.; Moore, H.; Zeidan, M. N.; Hwalla, N.; Summerbell, C. Health-E-PALS: Promoting healthy eating and physical activity in Lebanese school children - Intervention development. *Educ Health* **2014,** *32*, 3-8.
21. Evans, C. E. L.; Albar, S. A.; Vargas-Garcia, E. J.; Xu, F. School-Based Interventions to Reduce Obesity Risk in Children in High- and Middle-Income Countries. *Advances in Food and Nutrition Research* **2015,** *76*, 29-77.
22. Ministry of Public Health-Lebanon. *The MOH Strategic Plan*; 2007; Available online: https://extranet.who.int/nutrition/gina/sites/default/filesstore/LBN%202007%20The%20MOH%20Strategy%20Plan%20.pdf.
23. World Health Organization. *Health-promoting schools initiative in Oman. A WHO case study in intersectoral action*; World Health Organization: Cairo, Egypt, 2013; Available online: https://applications.emro.who.int/dsaf/EMROPUB_2013_EN_1587.pdf.
24. Government of Khyber Pakhtunkhwa. *Khyber Pakhtunkhwa Multi-sectoral Integrated Nutrition Strategy*; Planning and Development Department: 2014; Available online: https://extranet.who.int/nutrition/gina/sites/default/filesstore/PAK_2014_Khyber%20Pakhtunkhwa%20Integrated%20Nutrition%20Strategy.pdf.
25. Bajraktarevic, S.; Qadi, K.; Jouda, A.; Awadallah, Y.; Abueita, R. Improving the nutritional well-being of school-age children through the nutrition-friendly schools initiative (NFSI) in the State of Palestine. *Field Exchange - Emergency Nutrition Network ENN* **2021,** 47-50.
26. World Health Organization. *Nutrition action in schools: a review of evidence related to the nutrition-friendly schools initiative*; World Health Organization: Geneva, Switzerland, 2021; Available online: https://www.who.int/publications/i/item/9789241516969.
27. Ministry of Education and Higher Education-Palestine. General Directorate of School Health. Available online: https://www.mohe.ps/home/%D8%A7%D9%84%D8%A5%D8%AF%D8%A7%D8%B1%D8%A9-

%D8%A7%D9%84%D8%B9%D8%A7%D9%85%D8%A9-%D9%84%D9%84%D8%B5%D8%AD%D8%A9-%D8%A7%D9%84%D9%85%D8%AF%D8%B1%D8%B3%D9%8A%D8%A9/ (accessed on 23 February 2023).
28. Federal Ministry of Health-Republic of Sudan. *National Nutrition Policy and Key Strategies*; Maternal and Child Health Directorate: 2009; Available online: http://www.fmoh.gov.sd/Health-policy/nationalnutritionpolicy.pdf.
29. Federal Ministry of Health-Republic of Sudan. *National Nutrition Strategy & Key Strategies (2008 - 2012)*; Maternal and Child Health Directorate: 2008; Available online: https://extranet.who.int/nutrition/gina/sites/default/filesstore/SDN%202008_National%20Nutrition%20Policy.pdf.
30. Ministry of Health-Republic of Tunisia. *National Multisectoral Strategy for the Prevention and Control of Non-Communicable Diseases (NCD) 2018-2025*; 2018; Available online: https://extranet.who.int/nutrition/gina/sites/default/filesstore/TUN%202018%20Strat%C3%A9gie%20Nationale%20MNT.pdf.
31. Ministry of Health-Republic of Tunisia. *Obesity Prevention and Control Strategy*; 2012; Available online: https://extranet.who.int/nutrition/gina/sites/default/filesstore/TUN_2014_SPLO.pdf.
32. UAE Moments. The Emirates School Establishment to Launch a “Smart Canteen”. Available online: https://www.uaemoments.com/the-emirates-school-establishment-to-launch-a-%E2%80%9Csmart-canteen%E2%80%9D-513847.html (accessed on 2 June 2023).
33. The National News. Smart school canteen system to fight childhood obesity in UAE. Available online: https://www.thenationalnews.com/uae/health/smart-school-canteen-system-to-fight-childhood-obesity-in-uae-1.58622 (accessed on 2 June 2023).
34. Emirates News Agency-WAM. Launch of "The Fat Truth" Campaign to fight Child Obesity. Available online: https://wam.ae/en/details/1395228347008 (accessed on 2 June 2023).
35. Algurg, R.; Mahfouz, N. A.; Otaki, F.; Alameddine, M. Towards the Upscaling of School Nutrition Programs in Dubai: an Exploratory Study (pre-print). *Research Square* **2021**.
36. AlGurg, R. S.; Mahfouz, N. H. A.; Otaki, F. INTERVIEWS WITH KEY INFORMANTS TO EXPLORE SCHOOL NUTRITION PROGRAMS: A CASE-STUDY IN DUBAI. *IA13-COVER* **2020,** 106.
37. Bani-Issa, W.; Dennis, C. L.; Brown, H. K.; Ibrahim, A.; Almomani, F. M.; Walton, L. M.; Al-Shujairi, A. M. The Influence of Parents and Schools on Adolescents’ Perceived Diet and Exercise Self-Efficacy: A School-Based Sample From the United Arab Emirates. *Journal of Transcultural Nursing* **2020,** *31*, 479-491.
