# Supplementary material for: School-Based Nutrition Programs in the Eastern Mediterranean Region: A Systematic Review
Source: Int J Environ Res Public Health. 2023 Nov 10;20(22):7047. doi: 10.3390/ijerph20227047 (PMC10671197; doi:10.3390/ijerph20227047)
Supplement: Supplementary file 1 [file ijerph-20-07047-s001.zip › Table S5.pdf]

Table S5. Programs on School Staff Training in Countries of the EMR

Country	Reference	Year and Status	National or Regional	Leadership	Target Population	Objective	Brief Description of the Policy/Intervention
**Training of school staff (teachers, canteen staff, school health providers etc.)**							
**Afghanistan**	WHO GINA [1]	2009	National	MOPH	School teachers	Increase the awareness about nutrition among the general population, and provide caregivers with the knowledge, skills and support required to adopt healthy nutrition practices, using food-based approaches.	Developing a training strategy for the relevant staff i.e. school teachers.
**Bahrain**	Aldinger and Whitman 2009 [2]; AlMulla AlHarmas AlHajeri et al 2009 [3]	2004	Regional; Muharraq governorat e	MOH in collaboration with the MOE, GCC and WHO EMRO	School representatives (food canteen coordinator, school councilor, school nurse), teachers and staff	- Provide instruction to develop the knowledge, skills, attitudes, and behaviors related to healthy living.<br>- Support the provision of support services for students and their families.<br>- Create a healthy social and physical environment within the school.<br>- Integrate the concepts of personal health management, health promotion, and education.<br>- Incorporate strategies that are comprehensive, interdisciplinary, and outcome based.<br>- Be taught by teachers who are competent and qualified in health education and promotion.<br>- Provide sufficient instruction time to elicit behavior change.	**HPS program:**<br>Workshops about the projects were conducted for all the schools' representatives in order to implement school health promotion

**Egypt**	WFP 2022 [4]	2014	Regional; Assiut and Aswan	WFP, The National Nutrition Institute and MOHP	School health care providers	-	As part of **the National School Screening and Management Programme**, a training of trainers on early detection of and referral protocols of malnutrition, and optimal nutrition practices for school-aged children, was delivered for over 120 primary school health care providers.
**Iran**	Omidvar et al 2021 [5]	2007	Regional; rural	Ministry of Welfare and Social Security, MOHME and Welfare Organization	Kindergarten managers and coaches; kindergarten parents	Raise awareness about children's nutrition and development.	Raising the awareness of kindergarten managers and coaches about children's nutrition and development.<br>Enhancing parents' awareness about children's nutrition and development.
**Kuwait**	Behbehani 2014 [6]; Evans et al 2015 [7]	2013-2017	National	MOE; MOH; Private School Council; Ministry of Commerce; Kuwait Counsellor Network; Ministry of Labour and Social Affairs; Council of Religious Affairs	School staff	- Increase the prevalence of sustained PA among the population by 20%.<br>- Reduce mean BMI significantly by 2% among overweight and obese children, youths and adults.<br>- Reduce mean waist-hip ratio significantly by 5% among overweight and obese children, youths and adults.<br>- Reduce the prevalence of overweight adults in the population by 10%.<br>- Reduce the prevalence of overweight children and youths aged 6–18 years in the population by 15%.	**The Kuwait National Programme for Healthy Living:**<br>Training the school staff (i.e. teachers) regarding obesity, diabetes and other chronic conditions in order to ensure the delivery of correct content.

						- Reduce the mean energy intake among the population by 10%.	
	WHO 2018 [8]	2010	Regional	MOE and MOH	Kindergartens and schools	- Reduce or prevent child undernutrition (stunting, wasting, micronutrient deficiencies).<br>- Reduce or prevent childhood overweight or obesity.<br>- Foster healthy diet and lifestyle habits.<br>- Educate children and improve knowledge about healthy diet and lifestyle habits.<br>- Improve academic performance.	A nutrition Education program was designed and implemented to teach the home economics and science teachers all about relation of good nutrition with the healthy life style and how to transfer the knowledge to their students.
**Morocco**	WHO GINA [9] and Ministry of National Education [10]	2012 although published in 2011<br><br>Total duration 2011-2019	National	MOH	Teachers	- Improve the health status of the population by acting on one of its major determinants, nutrition.<br>- Promote a healthy lifestyle to prevent nutritional disorders and nutrition-related chronic diseases.<br>- Strengthen institutional and professional skills in nutrition.	**National Nutrition Strategy 2011-2019:**<br>- Reinforcement of basic and continuous training of teachers in nutrition.
**Pakistan**	Khan and Ajmal 2011 [11]	2005-2008	National (in 29 districts)	Initiated by the Federal Ministry of Women and Development<br><br>Supported by multilevel collaboration between the Aga Khan University, 11 local NGOs,	Teachers in 4035 government primary girls' schools; village women as well	- Reduce malnutrition.	4383 School Committees (STCs) were formed.<br>About 4400 community organizers and schoolteachers were trained; over 95 000 village women were also trained through a structured continuing education programme, and received information about malnutrition and its impact, what constituted a balanced diet, the importance of education for girls and its benefits.

				district governments, Pakistan Baitul Maal and the Federal Ministry			
**Palestine**	Bajraktarevic et al 2021 [12]; WHO 2021 [13]	2018-2021	National	UNICEF-supported intervention; supporting MOE and MOH	School teachers, administrators and school-aged children	- Establish healthy dietary and physical activity habits and improve the nutritional status of school-age children.<br>- Strengthen the involvement of parents, families and communities, complementing formal ongoing school interventions and creating an enabling environment for sustainable positive change around nutrition and healthy lifestyles.	**Nutrition Friendly Schools Initiative:**<br>Implementing a capacity-building programme for school teachers, administrators and school-aged children to develop their knowledge and raise awareness of optimal nutrition behaviours.<br>The capacity-building programme involved a two day training targeting 34 school principals followed by cascade training at school level for teachers and administrative staff.
**Tunisia**	WHO GINA [14, 15]	2012 and 2015-2016	National	MOH	Educators and teachers of Kindergartens and primary schools	Reduce morbidity, disabilities and premature mortality related to NCDs and their risk factors:<br>- Strengthen the promotion of healthy lifestyles and the prevention of NCDs.<br>- Ensure quality management of NCDs.<br>- Improve governance at all levels of competence and responsibility.<br>- Develop a monitoring and evaluation system for NCDs and their risk factors.	**Obesity Prevention and Control Strategy 2012; National Multisectoral Strategy for the Prevention and Control of Non-Communicable Diseases (NCD) 2018-2025:**<br>- Health-related basic training programmes for educators and teachers of preschools and schools are strengthened through nutrition and health education.

	Hrairi and Berger 2017 [16]	Implemented	Regional	The Chronic Disease Prevention Research Center (CDPRC) with the partnership of different national and international partners	School teachers and student leader groups	**-**	**"Together in Health"**<br>Information and training meetings for teachers were organized. Student leader groups were organized and trained in order to play the role of peers in the fight against unhealthy lifestyles.
**UAE**	MOE 2017 [17]	2017-2021	National	MOE	Teacher in all government schools; From kindergarten to grade 12	Empower young Emirati students to take ownership of their physical education, health and wellbeing to ensure a future generation of healthy, motivated, highly educated Emiratis.	**Physical and Health Education Curriculum**<br>**500 Trainees**<br>The MOE have exerted a lot of effort to establish and prepare teachers to teach the brand-new curriculum. More than 500 trainees of the Physical and Health Education have attended a training session about the launch of the new curriculum in different venues across the emirates, including the new Teacher Training Centre in Ajman.
	Emirates 24/7 [18]	2013	Regional; Abu Dhabi	Abu Dhabi Quality and Conformity Council (QCC); Abu Dhabi Education Council (ADEC)	Administrators, nutritionists, nurses and supervisors of school canteens and food establishments	- Protect the health and safety of students.<br>- Reduce the potential risks of chronic diseases among students such as diabetes and high blood pressure.	**New school canteen rules for Abu Dhabi**<br>- The manual serves as an important tool to train administrators, nutritionists, nurses and supervisors of school canteens and food establishments on appropriate handling of food items.

	The Health Authority Department of Health 2012 [19]	2011-2012 piloted; 2012-2013 expanded	Regional; Abu Dhabi	Government; The Health Authority – Abu Dhabi (HAAD); In Collaboration with Abu Dhabi Education Council (ADEC)	25 public schools	- Reduce illnesses caused by unhealthy life style among school children.	**“Eat Right Get Active”**<br>**-** Training workshop on Healthy Eating and Active Living for private schools representatives on how to administer the data and train students to approximate their food intake over the course of a typical week.
**Several countries: Lebanon, Jordan, Palestine, Bahrain, KSA and UAE**	Habib-Mourad et al 2022 [20]	2018	National	Public-private partnership: Nestlé Middle East FZE; AUB; MOE; MOH	School teachers of 9-11 year old students	- Tackle childhood obesity by addressing nutritional and physical activity habits of schoolchildren.<br><br>- Promote healthy eating and physical activity habits among 9–11-year-old student.<br><br>- Raise the degree of nutritional and health awareness among students, their families, school health and nutrition officials and teaching staff, and promote positive trends and modify incorrect trends.	Workshops were conducted in each country and consisted of a 2-day interactive face-to-face training of teachers, and hands-on coaching and role-plays.<br><br>Some changes to the local educational authorities and health institutions roles and structure in the UAE and KSA prevented the program continuation and affected sustainability; the program continues to be implemented in four out of six countries: Lebanon, Jordan, Palestine, and Bahrain.

Abbreviations: ADEC: Abu Dhabi Education Council; AUB: American University of Beirut; CDPRC: Chronic Disease Prevention Research Center; EMRO: Regional Office for the Eastern Mediterranean; FZE: free zone establishments; GCC: Gulf Cooperation Council; GINA: Global Database on the Implementation of Nutrition Action; HAAD: Health Authority – Abu Dhabi; HPS: health promoting schools; KSA: Kingdom of Saudi Arabia; MOE: Ministry of Education; MOH: Ministry of Health; MOHME: Ministry of Health and Medical Education; MOHP: Ministry of Health and Populations; MOPH: Ministry of Public Health; NCD: non-communicable diseases; NGOs: non-governmental organizations; QCC: Quality and Conformity Council; STCs: school committees; UAE: United Arab Emirates; UNICEF: United Nations International Children's Emergency Fund; WFP: World Food Programme; WHO: World Health Organization.

References

1. Ministry of Public Health-Afghanistan, *National Public Nutrition Policy and Strategy 2009-2013*. 2009.
2. Aldinger, C. and C.V. Whitman, *Case studies in global school health promotion: from research to practice*. 2009, New York, US: Springer.
3. AlMulla AlHarmasAlHajeri, M., L.A.A.A. Al Thukair, and N. Sarhan, *Bahrain: National Comprehensive School Health Program, Health-Promoting Schools.* Case Studies in Global School Health Promotion: From Research to Practice, 2009: p. 239-249.
4. World Food Programme, *Annual Report - World Food Programme, 2003*. 2004, World Food Programme: Rome, Italy. p. 51 pp.
5. Omidvar, N., et al., *Enabling food environment in kindergartens and schools in iran for promoting healthy diet: Is it on the right track?* International Journal of Environmental Research and Public Health, 2021. **18**(8).
6. Behbehani, K., *Kuwait national programme for healthy living: First 5-year plan (2013-2017).* Medical Principles and Practice, 2014. **23**(SUPPL. 1): p. 32-42.
7. Evans, C.E.L., et al., *School-Based Interventions to Reduce Obesity Risk in Children in High- and Middle-Income Countries.* Advances in Food and Nutrition Research, 2015. **76**: p. 29-77.
8. World Health Organization, *Global nutrition policy review 2016–2017: Country progress in creating enabling policy environments for promoting healthy diets and nutrition*. 2018, World Health Organization: Geneva, Switzerland.
9. Ministry of Health-Morocco; UNICEF, *La Stratégie Nationale de la Nutrition*. 2011.
10. Ministry of National education-Morocco. *Nutrition education*. 20 February 2023]; Available from: https://www.men.gov.ma/Fr/Pages/Edunutri.aspx.
11. Khan, K.S. and A. Ajmal, *Women's empowerment and its challenges: review of a multi-partner national project to reduce malnutrition in rural girls in Pakistan*. 2011, World Health Organization: Geneva. p. 117-128.
12. Bajraktarevic, S., et al., *Improving the nutritional well-being of school-age children through the nutrition-friendly schools initiative (NFSI) in the State of Palestine.* Field Exchange - Emergency Nutrition Network ENN, 2021(66): p. 47-50.
13. World Health Organization, *Nutrition action in schools: a review of evidence related to the nutrition-friendly schools initiative*. 2021, World Health Organization: Geneva, Switzerland.
14. Ministry of Health-Republic of Tunisia, *National Multisectoral Strategy for the Prevention and Control of Non-Communicable Diseases (NCD) 2018-2025*. 2018.
15. Ministry of Health-Republic of Tunisia, *Obesity Prevention and Control Strategy*. 2012.
16. Hrairi, S. and D. Berger, *Nutrition Education in Tunisian schools: analysis of practices reported by primary teachers.* QUESTIONS VIVES-RECHERCHES EN EDUCATION, 2017(27).
17. Ministry of Education-UAE. *Ministry of Education announces a brand new Physical and Health Education reform in the UAE*. 2017 16 March 2023]; Available from: https://www.moe.gov.ae/En/MediaCenter/News/Pages/sport.aspx.
18. Emirates 24/7. *New school canteen rules for Abu Dhabi*. 2015 2 June 2023]; Available from: https://www.emirates247.com/news/emirates/new-school-canteen-rules-for-abu-dhabi-2015-09-05-1.602379.
19. The Health Authority Department of Health-Abu Dhabi. *“Eat Right Get Active” Expands to include 50 Private Schools*. 2012 23 March 2023]; Available from: https://www.doh.gov.ae/en/news/eat-right-get-active-expands-to-include-50-private-schools.

20. Habib-Mourad, C., et al., *Ajyal Salima a novel public–private partnership model for childhood obesity prevention in the Arab countries.* Front Public Health, 2022. **10**.
