# Supplementary material for: School-Based Nutrition Programs in the Eastern Mediterranean Region: A Systematic Review
Source: Int J Environ Res Public Health. 2023 Nov 10;20(22):7047. doi: 10.3390/ijerph20227047 (PMC10671197; doi:10.3390/ijerph20227047)
Supplement: Supplementary file 1 [file ijerph-20-07047-s001.zip › Table S6.pdf]

Table S6. Programs on Marketing and Bans or Standards for Vending Machines in the School Setting in Countries of the EMR

Country	Reference	Year and Status	National or Regional	Leadership	Target Population	Objective	Brief Description of the Policy/Intervention
**Bans on vending machines in schools**							
**Bahrain**	WHO 2018 [1]	-	National	MOE and MOH in collaboration with the WHO, UNICEF and food suppliers	Kindergartens and schools	- Reduce or prevent child undernutrition (stunting, wasting, micronutrient deficiencies).<br>- Reduce or prevent childhood overweight or obesity.<br>- Educate children and improve knowledge about healthy diet and lifestyle habits.<br>- Improve school attendance.<br>- Improve academic performance.	Standards or rules apply to all foods and beverages being sold in vending machines.<br>- Criteria to determine which foods are prohibited, limited or encouraged are based on specific foods and beverages, nutrient content.
	WHO 2013 [2]	-	National	MOH	Kindergartens, primary and secondary schools	-	Vending machines not allowed on school premises.
**Egypt**	WHO 2018 [1]	-	National	MOE and MOH	Kindergartens and schools	- Reduce or prevent child undernutrition (stunting, wasting, micronutrient deficiencies).<br>- Reduce or prevent childhood overweight or obesity.<br>- Foster healthy diet and lifestyle habits.<br>- Educate children and improve knowledge about healthy diet and lifestyle habits.<br>-Improve children's skills (e.g. cooking, food hygiene).<br>- Improve school enrolment.<br>- Improve school attendance.<br>- Improve academic performance.	Vending machines not allowed in schools.

						- Tackle health inequalities.<br>- Reduce food insecurity and hunger.	
**Iran**	WHO 2018 [1]	2007	National	MOE and MOH	Kindergartens and schools	- Reduce or prevent child undernutrition (stunting, wasting, micronutrient deficiencies).<br>- Reduce or prevent childhood overweight or obesity.<br>- Foster healthy diet and lifestyle habits.<br>- Educate children and improve knowledge about healthy diet and lifestyle habits.<br>- Tackle health inequalities.<br>- Reduce food insecurity and hunger.	Standards or rules apply to foods and beverages being sold in vending machines.<br>- Criteria to determine which foods are prohibited, limited or encouraged are based on nutrient content.
**KSA**	WHO 2018 [1]	-	National	MOE and MOH	Kindergartens and schools	- Reduce or prevent child undernutrition (stunting, wasting, micronutrient deficiencies).<br>- Reduce or prevent childhood overweight or obesity.<br>- Foster healthy diet and lifestyle habits.<br>- Educate children and improve knowledge about healthy diet and lifestyle habits.<br>- Improve children's skills (e.g. cooking, food hygiene).<br>- Improve school enrolment.<br>- Improve school attendance.<br>- Improve academic performance.	Vending machines not allowed in schools.
**Kuwait**	WHO 2018 [1]	1987	National	MOE and MOH	Kindergartens and schools	- Reduce or prevent child undernutrition (stunting, wasting, micronutrient deficiencies).<br>- Reduce or prevent childhood overweight or obesity.	Standards or rules apply to foods and beverages being sold in vending machines.<br>- Criteria to determine which foods are prohibited, limited or

						- Foster healthy diet and lifestyle habits.<br>- Educate children and improve knowledge about healthy diet and lifestyle habits.<br>- Improve academic performance.	encouraged are based on specific foods and beverages, nutrient content.
	WHO 2018 [1]; WHO 2013 [2]	-	National	MOE and MOH	Kindergartens, primary and secondary schools	-	Vending machines not allowed on school premises.
**Lebanon**	WHO 2018 [1]	1980	National	MOEHE and MOPH, in addition to WHO, UNICEF, local NGOs	Kindergartens and schools	- Reduce or prevent childhood overweight or obesity.<br>- Foster healthy diet and lifestyle habits.<br>- Educate children and improve knowledge about healthy diet and lifestyle habits.<br>- Tackle health inequalities.	Vending machines not allowed on school premises.
**Oman**	WHO 2018 [1]	1996	National	MOE and MOH	Kindergartens and schools	- Reduce or prevent child undernutrition (stunting, wasting, micronutrient deficiencies).<br>- Reduce or prevent childhood overweight or obesity.<br>- Foster healthy diet and lifestyle habits.<br>- Educate children and improve knowledge about healthy diet and lifestyle habits.	Standards or rules apply to foods and beverages being sold in vending machines.
	WHO 2018 [1]; WHO 2013 [2]	1996	National	MOE and MOH, in collaboration with schools and regional muncipalities,	Kindergartens and schools (primary and secondary)	- Reduce or prevent child undernutrition (stunting, wasting, micronutrient deficiencies).<br>- Reduce or prevent childhood overweight or obesity.	Vending machines not allowed on school premises.

				schools' adminstrators, school health teams		- Foster healthy diet and lifestyle habits.<br>- Educate children and improve knowledge about healthy diet and lifestyle habits.<br>- Improve school attendance.<br>- Improve academic performance.	
**Qatar**	WHO 2018 [1]	2010	National	MOEHE, MOPH, Ministry of Municipality and Environment, as well as Qatar Diabetes Association	Kindergartens and schools	- Reduce or prevent childhood overweight or obesity.<br>- Foster healthy diet and lifestyle habits.	Standards or rules apply to all foods and beverages being sold in vending machines.<br>- Criteria to determine which foods are prohibited, limited or encouraged are based on specific foods and beverages, nutrient content, portion size.
**UAE**	WHO GINA [3]	2017-2021	National	MOHAP	Schools and their environment	Improve the nutritional status of all population residing in the UAE with a collective vision of a healthier and sustainable future; guided by the international, regional and national policies and strategies to promote health.<br><br>**Strategic objectives:**<br>- Reduce morbidity and mortality from NCDs by following healthy diet and physical activity, through achieving the following targets:<br>10% relative reduction in prevalence of insufficient physical activity.<br>30% relative reduction in mean population intake of salt.	**National Action Plan in Nutrition 2017-2021:**<br>- Ban the food products and beverages sold in school vending machines.

						Reduce the prevalence of obesity among children (5-17 years) by 2.4% (baseline 14.4% target 12%).	
**School canteens or standards or rules for foods and beverages available in schools**							
**Bahrain**	Garemo et al 2019 [4]; Public Health Directorate Bahrain 2014 [5]	-	National	-	School canteens	Cover major life cycle and nutrition related health problems.	A School Canteen List has been released, which includes foods and beverages that are and are not permitted on school premises. Examples of non-permitted beverages include sweetened fruit drinks, nectars, soft drinks and energy drinks. Banned food items include processed meat, mayonnaise, potato chips, puff pastries, sweets and candies.<br><br>The Bahraini government has endorsed the Hygiene Conditions for School Canteens and Handled Food (GSO 1971/2014). These standards, issued by GCC countries, include details on foods that are not permitted in the school premises.<br><br>Bahrain has also updated school meals menu for canteen suppliers in 2014.
	WHO 2018 [1]	-	National	MOE and MOH in collaboration with the	Kindergartens and schools	- Reduce or prevent child undernutrition (stunting, wasting, micronutrient deficiencies).	Standards or rules apply to all foods and beverages being sold in school shops/stores, foods

				WHO, UNICEF and food suppliers		- Reduce or prevent childhood overweight or obesity.<br>- Educate children and improve knowledge about healthy diet and lifestyle habits.<br>- Improve school attendance.<br>- Improve academic performance.	and beverages available at school events (e.g. sports days).<br>- Criteria to determine which foods are prohibited, limited or encouraged are based on specific foods and beverages, nutrient content.
	MOH [6]	2018		MOH in collaboration with MOE	School canteens	Enhance the food prepared in school canteens quantity and quality wise.	Workshop for school canteen operators:<br>- Enhance the food prepared in school canteens, quantity and quality wise, by assisting canteens' operators to prepare healthy food and introduce them to nutrition requirements and standards for making the food and beverages provided to student through the canteens.
	Information provided by NFP	2017	National	MOH and MOE	School canteens	-	Eliminate TFA from school canteens through the adoption of a prohibited food list and by modification of food preparation/cooking methods.
	Aldinger and Whitman 2009 [7]; AlMulla AlHarmasAlHajeri et al 2009 [8]	2004	Regional; Muharraq governorate	MOH in collaboration with the MOE, GCC and WHO EMRO	School cafeterias	- Provide instruction to develop the knowledge, skills, attitudes, and behaviors related to healthy living.<br>- Support the provision of support services for students and their families.<br>- Create a healthy social and physical environment within the school.<br>- Integrate the concepts of personal health management, health promotion, and education.	Improve school cafeteria food.

						- Incorporate strategies that are comprehensive, interdisciplinary, and outcome based.<br>- Be taught by teachers who are competent and qualified in health education and promotion. - Provide sufficient instruction time to elicit behavior change.	
**Egypt**	Information provided by NFP; Ministry of Education and Technical Education [9]	2019	National	MOH and Ministry of Education and Technical Education	School	- Improve the healthy nutrition of school students to treat and prevent diseases resulting from malnutrition.<br>- Intensify awareness about healthy, useful eating, and healthy meals appropriate for the appropriate age group.	**100 Million Health Initiative:** Provide healthy foods within the food outlets in schools, as well as providing outlets for selling food in the vicinity of schools and public places to provide healthy and safe foods.
	WHO 2018 [1]	-	National	MOE and MOH	Kindergartens and schools	- Reduce or prevent child undernutrition (stunting, wasting, micronutrient deficiencies).<br>- Reduce or prevent childhood overweight or obesity.<br>- Foster healthy diet and lifestyle habits.<br>- Educate children and improve knowledge about healthy diet and lifestyle habits.<br>-Improve children's skills (e.g. cooking, food hygiene).<br>- Improve school enrolment.<br>- Improve school attendance.<br>- Improve academic performance.<br>- Tackle health inequalities.<br>- Reduce food insecurity and hunger.	Standards or rules apply to foods and beverages served at other mealtimes (e.g. breakfast, after-school services).

**Iran**	Garemo et al 2019 [4]; Yazdi-Feyzabadi et al 2019 [10]	-	National	MOH and the Health Affairs Office affiliated with the MOE	School canteens	**-**	**National Guideline for Healthy Food Canteen in Schools:**<br>- Guideline includes a list of healthy and unhealthy foods.<br>- In this guideline, snacks that are low in nutrients and high in fat, sugar and salt are considered unhealthy.
	Omidvar et al 2021 [11]; Sayyari et al 2017 [12]	2016	-	MOE and MOHME	Kindergartens and school canteens (2-18 years), and their environments	- Improve population health and health equity in childhood obesity prevention.<br>- Reduce the incidence of childhood obesity and identify and treat preexisting obesity in children and adolescents.	**The IRAN-Ending Childhood Obesity program:**<br>- Increase the number of schools with healthy canteens and kindergartens with a healthy eating plan.<br>- Limit the supply of unhealthy snacks and fast foods by street food vendors around the schools.<br>- Ban the sale of unhealthy foods and drinks.<br>- Promote healthy school environments.
	Omidvar et al 2021 [11]; WHO EMRO 2018 [13]; WHO EMRO [14] Ministry of Health and Medical Education 2017 [15]; Babashi et al 2021 [16]; Feyzabadi et al 2017 [17]	2014	National	MOE and MOHME	School environment and canteens; students aged 7-18 years	- Increase access to healthy snacks.<br>- Prevent the supply of foods with low nutritional value.	**Healthy schools canteen bylaw:**<br>- Increase access to healthy snacks.<br>- Prevent the supply of foods with low nutritional value.<br>- Chips, cookies, crackers, ice cream, fried foods, sugary drinks, sweet biscuits, hamburgers, pizza, and

							confectionary are banned in schools.<br>- Healthier food and drink choices should be provided in school canteens.<br>- Set up a canteen to supply food items according to the permitted foods list recommended by the HSC bylaw. In this bylaw, healthy food items are appropriate to one's nutritional needs, are safe, varied, balanced, low in salt, low in fat, and have less than 5% of trans fatty acids.
	Al-Jawaldeh et al 2020 [18]; WHO EMRO 2018 [13]	-	-	MOH and MOE	School canteens	-	The sale of unhealthy food in school canteens and by vendors around schools is also prohibited.
	Sartipizadeh et al 2021 [19]; Yazdi-Feyzabadi et al 2019 [10]	2010 Piloted	5 provinces	MOE and MOHME	School environment and cafeteria	- Develop concepts of self-care and health promotion in both individual and society or community aspects and as an integrated and coordinated system for school health programs.<br>**-** Improve high-risk behaviours of adolescents.	**Iranian health promoting schools (IHPSs) program:**<br>- Present allowed and disallowed food stuff instructions exposed to the students' sight.<br>- In some Iranian schools, this program is implemented and one of the components covered is nutritional behaviours.
	WHO 2018 [1]	2007	National	MOE and MOH	Kindergartens and schools	- Reduce or prevent child undernutrition (stunting, wasting, micronutrient deficiencies).<br>- Reduce or prevent childhood overweight or obesity.	Standards or rules apply to foods and beverages served for lunch in school canteens/cafeterias, packed lunches and other foods or

						- Foster healthy diet and lifestyle habits.<br>- Educate children and improve knowledge about healthy diet and lifestyle habits.<br>- Tackle health inequalities.<br>- Reduce food insecurity and hunger.	beverages brought from home, all foods and beverages being sold in school shops/stores.<br>- Criteria to determine which foods are prohibited, limited or encouraged are based on nutrient content.
**Iraq**	WHO GINA [20, 21]	2018-2022	National	General Secretariat for the Council of Ministers (MOH; in collaboration with the MOE, MOF)	School students	Reduce morbidity and premature mortality due to avoidable chronic NCDs in order to achieve the highest attainable level of health and productivity for all ages to promote economic and social development.	National strategy for the prevention and control of non-communicable diseases 2018-2022:<br>- Check the nutritional characteristics of the foods provided in the schools, according to the National Food-Based Nutrition guidelines.
	WHO 2018 [1]	2010	National	MOE and MOH	Kindergartens and schools	- Reduce or prevent child undernutrition (stunting, wasting, micronutrient deficiencies).<br>- Reduce or prevent childhood overweight or obesity.<br>- Foster healthy diet and lifestyle habits.<br>- Educate children and improve knowledge about healthy diet and lifestyle habits.<br>- Improve children's skills (e.g. cooking, food hygiene).<br>- Improve school enrolment.<br>- Improve school attendance.<br>- Improve academic performance.<br>- Tackle health inequalities.<br>- Reduce food insecurity and hunger.	Standards or rules apply to foods and beverages served for lunch in school canteens/cafeterias.<br>- Criteria to determine which foods are prohibited, limited or encouraged are based on nutrient content.

						- Support the agriculture sector by creating farm to school linkages (e.g. cereals, milk, fruit and vegetables supply).	
	WHO GINA [22]	2012-2021	National	General Secretariat for the Council of Ministers	Schools and their canteens	- Ensure the health and nutrition status.<br>- Improve the nutritional status of Iraqi people throughout the life cycle from 2012 to 2021.<br>- Reduce prevalence of wasting and stunting among children under five years of age.<br>- Reduce prevalence of overweight and obesity among all age groups.<br>- Provide nutritional health promotion and counseling to people at community level.<br>- Reduce prevalence of micronutrient deficiencies.<br>- Assure safe food availability for all age groups at any time.<br>- Ensure adequate food available, accessible and utilized for all age groups at any time.	National Nutrition Strategy 2012-2021:<br>- Promote healthy options in canteens.<br>- Encourage schools to adopt healthy diets.

**Jordan**	MOE 2012 [23] and WHO EMRO 2018 [13]	2012	National	MOE	School canteens	-	**Health requirements for school canteens and foods allowed and prohibited to be sold for the year 2012**<br>*Food items that have been prohibited from being sold at school canteens:*<br>- Foods that are stale; instead they should be disposed of daily.<br>- Soft drinks of all kinds, hand-prepared juice and industrial juice; students are prevented from bringing them to school.<br>- Drinks containing only sugar and colourings.<br>- Drinks and juices with less than 30% fruit juice.<br>- Desserts, chocolate, gum, candies and lollipops with low nutritional value of all kinds; toffee and nouga that stick to teeth.<br>- Biscuits of all kinds and prepackaged cakes.<br>- Foods and beverages in glass packaging.<br>- Meat and liver of all kinds.<br>- Eggs.<br>- Chips not produced from potato chips and natural corn.<br>- Ice-cream.

	Evans et al 2015 [24]	2012	National	MOH	Schools	- Reduce the intake of fat and sugar consumed by students.	**National food standard regulations as a school health strategy:**<br>- A ban was introduced on fizzy drinks, crisps, and chocolates in schools.<br>- Offer fruit and vegetables for breakfast at the shops in schools.
	WHO GINA [25]	2006	National	MOH and WHO	Infants, toddlers and adolescents	- Reduce the prevalence and burden of diet-related diseases.<br>- Ensure that the food products are safe in terms of health.<br>- Control of nutritional disorders including micronutrient deficiencies.<br>- Control of communicable diseases and NCDs.<br>- Balance of food intake and physical exercises.<br>- Legislation on food security and safety.	**Nutrition in Jordan Update and plan of Action:**<br>- Develop good dietary practices in school canteens with active participation of students and teachers.<br>- Promote healthy nutrition in school canteens.
	WHO 2018 [1]	1999	National	MOE and MOH	Kindergartens and schools	- Reduce or prevent child undernutrition (stunting, wasting, micronutrient deficiencies).<br>- Improve academic performance.<br>- Reduce food insecurity and hunger.	Standards or rules apply to foods and beverages served at other mealtimes (e.g. breakfast, after-school services), Packed lunches and other foods or beverages brought from home.<br>- Criteria to determine which foods are prohibited, limited or encouraged are based on specific foods and beverages.
**KSA**	MOE [26]	2022	National	MOE and MOH	Schools	-	Guidelines for the provision of school feeding services:

							Included a list of allowed foods and another list for restricted foods<br>*Allowed foods:*<br>- Milk: fresh or dry powder milk (UHT, full fat or low fat) and fortified with vitamin D and Calcium; milk with natural flavours.<br>- Zabadi: Prepared zabadi and fruit-flavoured zabadi (natural).<br>- Juices: 100% fresh juices without any preservatives and colourings; packaged juices that contain at least 30% of fruit juice.<br>- Sandwiches and pies: Arabic bread, Samooli or toast, preferably brown bread; low fat and low salt cheeses to be used; sandwiches and pies that contain cheese, labneh, thyme, jam, honey, legumes; peanut butter for intermediate and secondary school students; pizzas, manaeesh; egg sandwiches; potato pies (baked or mashed or boiled).<br>- Fresh and seasonal fruits and vegetables.<br>- Dried fruits, except those coated with sugar or other sweeteners.<br>- Rice, whole grain breakfast cereals, oats, granola.

							- Popcorn, regular<br>- Biscuits and maamoul made from whole flour or regular (unsalted).<br>- Unsalted nuts.<br><br>*Restricted foods:*<br>- Carbonated beverages; Energy and sports drinks; Flavoured water; Cold tea; Packaged juices that contain less than 30% of fruit juice; Fruit syrups; Milk and zabadi with artificial flavours and colourings; All kinds of meat (red meats, white meats, chicken and fish); Sausages and hot dogs and other processed meats like mortadella and luncheon meats; Sandwiches and pies containing custard, chocolate or toffee or vanilla; All kinds of chips and deeply fried foods; Salted nuts; Icecream; Sweets containing sugar and colourings; chewing gum and sticky sweets; Puffed grains; All kinds of chocolate, including wafers; Pickled foods; Mayonnaise and butter; Salted foods; Doughnuts; Foods containing MSG; Croissants; Peanut butter for primary students.

	Al-Eid et al 2017 [27] and MOH 2017 [28]	2017-2020	National	MOH in cooperation with the MOE	School-aged students	- Increase awareness about the importance of a healthy lifestyle.<br>- Improve the school environment.<br>**-** Reduce the prevalence of obesity among school-aged children and adolescents in some selected schools at a rate of 5% by the end of 2020.<br>**-** Improve the nutritional behavior among students.<br>- Provide the preventive and therapeutic services to overweight and obese students.	**The RASHAKA Initiative:**<br>- Provide healthy food choices in school cafeterias (fruits, vegetables, whole-grain bread, etc.), and inhibit the sales of high-energy (high fat and sugar) snacks and drinks.
	WHO GINA [29]	2017-2027	National	MOH	School canteens	Keep the incidence rate of obesity unchanged in the coming 10 years:<br>- Primary protection against obesity - reduction of the incidence rate of obesity through controlling its risk factors.<br>- Secondary protection against obesity – early detection of cases.<br>- Improving the quality of the three-level health services provided for the obese people.<br>- Supporting the means of evaluation, follow-up, surveys, and studies on obesity.<br>- Enabling the obese people and their families to contribute to controlling obesity and its complications, in addition to playing a role in providing health services and ensuring their quality.<br>- Promoting the community partnership to combat obesity.	**Obesity Control Program:**<br>- Improve food quality provided by school canteens.

	Garemo et al 2019 [4]	-	-	-	Children aged 6-18 years	**-**	**Health requirements for School Canteens:**<br>- Provide specific regulations regarding energy (Less than 200 kcal), salt, fat (less than 35% of kcal of total fat, less than 10% of kcal of saturated fat), and fibre (1.5 grams) content for individual food products provided at schools.<br>- Provide a list of foods not allowed to be supplied by the school: which includes fizzy drinks, energy drinks, ice tea, caffeinated drinks, meat, poultry and fish, processed meats, salted nuts, confectionaries, pickles, mayonnaise.
	Editorial 2019 [30]	-	-	-	Schools	Reverse overweight and obesity among growing children	Review school meals.
	WHO 2018 [1]	-	National	MOE and MOH	Kindergartens and schools	- Reduce or prevent child undernutrition (stunting, wasting, micronutrient deficiencies).<br>- Reduce or prevent childhood overweight or obesity.<br>- Foster healthy diet and lifestyle habits.<br>- Educate children and improve knowledge about healthy diet and lifestyle habits.<br>- Improve children's skills (e.g. cooking, food hygiene).	Standards or rules apply to foods and beverages served at other mealtimes (e.g. breakfast, after-school services), Packed lunches and other foods or beverages brought from home, foods and beverages available at school events (e.g. sports days). - Criteria to determine which foods are prohibited, limited or encouraged are

						- Improve school enrolment.<br>- Improve school attendance.<br>- Improve academic performance.	based on specific foods and beverages, nutrient content.
**Kuwait**	Garemo et al 2019 [4]			MOE and MOH	School premises	Improve the health among children in Kuwait.	- Ban of unhealthy foods and beverages such as fizzy drinks, crisps, chocolates and similar food on school premises.<br>- Promote increased intake of fruits and vegetables from the canteens during breakfast.
	MOH et al 2021 [31]; WHO EMRO 2016 [32]	-	National	Led by the FNA on behalf of the Kuwait MOH, in partnership with the WHO and the food industries	School canteens	Improve the health among children in Kuwait.	- Improve school canteen food options, so that only the Nectar products with reduced sugar content are allowed into the school canteens.
	Behbehani 2014 [33]; Evans et al 2015 [24]	2013-2017	National	MOE; MOH; Private School Council; Ministry of Commerce; Kuwait Counsellor Network; Ministry of Labour and Social Affairs; Council of Religious Affairs	Schools	- Increase the prevalence of sustained PA among the population by 20% and reduce.<br>- Reduce mean BMI significantly by 2% among overweight and obese children, youths and adults.<br>- Reduce mean waist-hip ratio significantly by 5% among overweight and obese children, youths and adults.<br>- Reduce the prevalence of overweight adults in the population by 10%.<br>- Reduce the prevalence of overweight children and youths aged 6–18 years in the population by 15%.	**The Kuwait National Programme for Healthy Living:**<br>Provide healthy food options in all school shops.

						- Reduce the mean energy intake among the population by 10%.	
	WHO 2018 [1]	1987	National	MOE and MOH	Kindergartens and schools	- Reduce or prevent child undernutrition (stunting, wasting, micronutrient deficiencies).<br>- Reduce or prevent childhood overweight or obesity.<br>- Foster healthy diet and lifestyle habits.<br>- Educate children and improve knowledge about healthy diet and lifestyle habits.<br>- Improve academic performance.	Standards or rules apply to foods and beverages served for lunch in school canteens/cafeterias, foods and beverages served at other mealtimes (e.g. breakfast, after-school services), Packed lunches and other foods or beverages brought from home, all foods and beverages being sold in school shops/stores, foods and beverages available at school events (e.g. sports days).<br>- Criteria to determine which foods are prohibited, limited or encouraged are based on specific foods and beverages, nutrient content.
**Lebanon**	Garemo et al 2019 [4]	-	-	MOPH	Nurseries	-	National Guidelines for Early Childhood include recommendations of sending the canteen menus home to parents on a weekly basis.
	WHO 2018 [1]	1980	National	MOEHE and MOPH, in addition to WHO, UNICEF, local NGOs	Kindergartens and schools	- Reduce or prevent childhood overweight or obesity.<br>- Foster healthy diet and lifestyle habits.<br>- Educate children and improve knowledge about healthy diet and lifestyle habits.<br>- Tackle health inequalities.	Standards or rules apply to foods and beverages served for lunch in school canteens/cafeterias.<br>- Criteria to determine which foods are prohibited, limited or encouraged are based on nutrient content.

**Morocco**	WHO 2018 [1]	-	National	MOE and MOH in addition to WHO, UNICEF and WFP	Kindergartens and schools	- Foster healthy diet and lifestyle habits.<br>- Educate children and improve knowledge about healthy diet and lifestyle habits.<br>- Improve children's skills (e.g. cooking, food hygiene).<br>- Improve school enrolment.<br>- Improve school attendance.<br>- Improve academic performance.<br>- Tackle health inequalities.	Standards or rules apply to foods and beverages served for lunch in school canteens/cafeterias.<br>- Criteria to determine which foods are prohibited, limited or encouraged are based on specific foods and beverages, nutrient content.
	WHO GINA [34] and Ministry of National Education [35]	2012 although published in 2011<br><br>Total duration 2011-2019	National	MOH	School canteens and environment; boarding schools	- Improve the health status of the population by acting on one of its major determinants, nutrition.<br>- Promote a healthy lifestyle to prevent nutritional disorders and nutrition-related chronic diseases.<br>- Strengthen institutional and professional skills in nutrition.	**National Nutrition Strategy 2011-2019:**<br>- Apply and strengthen quality standards in school canteens.<br>- Promote the consumption of foods rich and/or enriched in micronutrients.<br>- Develop a guide of dietary and health guidelines for the preparation of menus in school canteens and boarding schools.
**Oman**	WHO 2013 [36]; Aldinger and Whitman 2009 [7]	2004 – 2009 (for four academic years)	Regional	WHO global initiative, with representation from MOH and MOE	School canteens; implemented in 19 schools	- Raise health awareness of the students and their families.<br>- Provide comprehensive health services that deal with the physical, mental, and social health needs and problems of this population.<br>- Ensure healthy school environments.<br>- Create a healthier environment and lifestyle in schools and in society.	**HPS Initiative:**<br>- Review and amend the school canteen polices<br>- Enable healthy foods in school canteens
	WHO 2018 [1]	1996	National	MOE and MOH	Kindergartens and schools	- Reduce or prevent child undernutrition (stunting, wasting, micronutrient deficiencies).	Standards or rules apply to all foods and beverages being sold in school shops/stores.

						- Reduce or prevent childhood overweight or obesity.<br>- Foster healthy diet and lifestyle habits.<br>- Educate children and improve knowledge about healthy diet and lifestyle habits.	
**Pakistan**	WHO EMRO [14]	2020	National	Government of Pakistan	Schools	- Aim to address micronutrient malnutrition among Pakistani adolescents.	**Adolescent Nutrition Supplementation Guidelines:**<br>- Prohibit the sale of unhealthy snacks and drinks in schools, nutrition standards for school meals.
	WHO GINA [37]	2014	Regional; Khyber Pakhtunk	Government - Department of Elementary and Secondary School Education	School canteens	- Improve basic facilities in primary schools.	- Regulate schools' canteen and vendors for nutritious and safe foods: Instructions to schools for ensuring availability of milk, fruits and good quality/standard snacks and discourage sale of candies, toffees and junk foods to the students.
**Palestine**	MOEHE [38]	-	National	MOEHE	School canteens	- Provide students with healthy eating habits and behaviours.<br>- Improve the nutritional status in schools.<br>- Ensure good and healthy food quality and ensure that it is free from contamination.	- Ensure good quality and healthy food.
**Qatar**	WHO GINA [39]	2018-2019	National	MOEHE;<br>MOH	School canteens	- Enhance the nutritional and educational role of the canteen.	<u>Guidance for supervisors of school canteens for the academic year 2018-2019:</u><br>Provides a list of daily allowed foods, weekly allowed foods

							and prohibited foods in school canteens.
	WHO 2018 [1]	2010	National	MOEHE, MOPH, Ministry of Municipality and Environment, as well as Qatar Diabetes Association	Kindergartens and schools	- Reduce or prevent childhood overweight or obesity.<br>- Foster healthy diet and lifestyle habits.	Standards or rules apply to all foods and beverages being sold in school shops/stores. - Criteria to determine which foods are prohibited, limited or encouraged are based on specific foods and beverages, nutrient content, portion size.
	Garemo et al 2019 [4]	2007	-	-	Preschoolers aged 3-6 years; child-care facilities	-	**Healthy and safety standards:**<br>- Food prepared should have a wide variety, and the quantity and quality should meet the nutritional needs of the children.<br>- Food brought from home should be in accordance with the nursery's dietary guidelines and goals to establish healthy eating habits.
	Garemo et al 2019 [4]; Choudhury et al 2018 [40]	-	National	MOE	School canteens; 191 public independent schools, where more than 100,000 students are enrolled.	Promote healthy eating behaviors among elementary schoolchildren, aiming to diminish the future impact of obesity and type diabetes mellitus in Qatar.	Foods high in fat, salt and sugar will no longer be served in government-funded independent schools.<br><br>Banned foods include chocolate, chips, fried foods, processed meats and sugary drinks.<br><br>The mandate also included the following:

							- Cakes and biscuits will now only be served once a week instead of twice.<br>- Increase the provision of milk and dairy products, fruits, vegetables and salads.<br>- Replace refined grain products with wholegrain products.<br>- Decrease the frequency of serving of biscuits and cakes to once weekly.
**UAE**	MOH [41]	2019	National	MOH	Schools	**-**	**Healthy recipes for school lunch bags:**<br>Voluntary; provide healthy guidelines.
	Garemo et al 2019 [4]	2017	Regional; Dubai	Dubai Municipality	Children 4 years and above	**-**	**Guidelines and Requirements for Food and Nutrition in Schools in Dubai**<br>**-** Provide guidance on portion sizes, lists of non-permitted foods, and information on how to pack lunch boxes.
	Abdullatif et al 2022 [42]; Ahmed 2011 [43]; Khaleej Times 2017 [44]	2011 and in 2017 an update of this policy was released	Regional; Dubai	Government; Dubai Health Authority (DHA)	School canteens	Improve health to prevent overweight and obesity among students.	-Ban the sale of highly concentrated sugary drinks, carbonated drinks, energy drinks, all types of fruit drinks, high sugar food content such as chocolate, candy, sweets, chewing gum, as well milk and yogurt with synthetic flavors.<br>- Ban foods with high content of saturated fat, artificial flavors, monosodium, sodium such as chips, processed meat such as

							sausages, mortadella, pastrami, instant noodles.<br>- The regulations will also limit the sale of food and drinks that are harmful to children with allergies, diabetes and other conditions.<br>- Encourage healthy food options, such as fresh fruits and vegetables.<br>- School meals will be inspected for nutritional value.
	WHO GINA [3]	2017-2021	National	MOHAP	Schools and their environment	Improve the nutritional status of all population residing in the UAE with a collective vision of a healthier and sustainable future; guided by the international, regional and national policies and strategies to promote health.<br><br>**Strategic objectives:**<br>- Reduce morbidity and mortality from NCDs by following healthy diet and physical activity, through achieving the following targets:<br>10% relative reduction in prevalence of insufficient physical activity.<br>30% relative reduction in mean population intake of salt.<br>Reduce the prevalence of obesity among children (5-17 years) by 2.4% (baseline 14.4% target 12%).	**National Action Plan in Nutrition 2017-2021:**<br>- Standards for foods and drinks sold in schools.<br>- Regulate the availability of healthy foods in the school environment.

	Algurg et al 2021 [45]; AlGurg et al 2020 [46]; Garemo et al 2019 [4]	2011<br><br>2017	Regional; Dubai	Dubai Municipality and DHA<br><br>Food Safety Department of Dubai Municipality	School canteens	Reduce obesity prevalence to 12% by the year 2021.	**School canteen standards and guidelines**<br>- The guidelines incorporate age specific nutrient requirements, energy distribution per meal, recommended food groups and number of servings. Moreover, it also provides examples of compliant meals and food that can be consumed on special diets or in the case of allergens. It further includes food preparation guidelines.<br><br>**Food labeling**<br>Guidelines to ensure that children have access to nutritious, safe, and wholesome food during their school time.
	Emirates 24/7 [47]	2013	Regional; Abu Dhabi	Abu Dhabi Quality and Conformity Council (QCC); Abu Dhabi Education Council (ADEC); relevant stakeholders.	Government and private schools, educational institutions, as well as relevant authorities and food establishments that cater to school canteens in Abu Dhabi	- Protect the health and safety of students.<br>- Reduce the potential risks of chronic diseases among students such as diabetes and high blood pressure.	**New school canteen rules for Abu Dhabi**<br>**-** Outline the rules and health requirements that are to be met by school canteens and food establishments and details the health guidelines and practices to be followed across the different stages of food sale, cooking, handling, distribution and disposal.<br>- Ensure that the requirements for healthy meals are met by suppliers to school canteens.

	Garemo et al 2019 [4]	2012 and updated in 2017	-	-	Preschool children	**-**	**Health Requirements for Nurseries**<br>According to the requirements it is not permitted to provide fast food or food/meals with low nutritional value (such as sweets, chips and soft drinks) for children.
	Abu Dhabi Education Council 2014 [48]	2012, reviewed in 2014	Regional; Abu Dhabi	ADEC	School canteens in public schools	**-**	**ADEC Public Schools Policy Manual**<br>*Policy 4220: School Canteens and Healthy Eating*<br><br>- Ensure that school canteens comply with the current School Canteen standards and promote healthy eating within their school communities.<br><br>All ADEC schools shall adhere at all times to:<br>• Nutrition and healthy eating standards.<br>• Food specifications and requirements.<br>• Age- appropriateness of food.<br>• List of food items allowed in school canteens.<br>• Food allergies.
	Aldinger and Whitman 2009 [7]	2004	National	MOH	School canteens and cafeterias	Promote, protect, and improve the health of students in UAE, through a comprehensive and coordinated school health program.	**HPS project**<br>Ensure that all types of food offered in school canteens and cafeterias, together with their

							methods of preparation, transportation, and storage, meet required standards.
**Several countries: Lebanon, Jordan, Palestine, Bahrain, KSA and UAE**	Habib-Mourad et al 2022 [49]; Habib-Mourad et al 2014 [50]; MOE Jordan [51]; Evans et al 2015 [24]	2010 Lebanon; 2015 Jordan<br><br>(3-4 months duration, each)	National	Public-private partnership: Nestlé Middle East FZE; AUB; MOE; MOH	School canteens	- Tackle childhood obesity by addressing nutritional and physical activity habits of schoolchildren.<br><br>- Promote healthy eating and physical activity habits among 9–11-year-old student.<br><br>- Raise the degree of nutritional and health awareness among students, their families, school health and nutrition officials and teaching staff, and promote positive trends and modify incorrect trends.	Provide healthy food choices, offering less energy dense snacks and drinks options.<br><br>Provide relevant recommendations to include a healthy list of snacks and drinks and exclude unhealthy choices for children in the school shop.<br><br>The program continues to be implemented in four out of six countries: Lebanon, Jordan, Palestine, and Bahrain.
**Standards for marketing of food and non-alcoholic beverage**							
**Bahrain**	WHO EMRO [14]	-	-	-	School	-	- Restrict the marketing and provision of energy drinks.<br>- Banned carbonated drinks in 1997 and has recently proposed to regulate the marketing of energy drinks.
	Garemo et al 2019 [4]; WHO 2013 [2]	-	National	MOH	Kindergartens, primary and secondary school premises	Improve the nutrition status of children in Bahrain.	Marketing of unhealthy foods like high-fat/energy and micronutrient-poor foods and beverages are not allowed on premises.
	WHO 2018 [1]	-	National	MOE and MOH in collaboration with the	Kindergartens and schools	- Reduce or prevent child undernutrition (stunting, wasting, micronutrient deficiencies).	Standards for marketing of food and non-alcoholic beverages to children in school setting.

				WHO, UNICEF and food suppliers		- Reduce or prevent childhood overweight or obesity.<br>- Educate children and improve knowledge about healthy diet and lifestyle habits.<br>- Improve school attendance.<br>- Improve academic performance.	
**Iran**	WHO EMRO 2018 [13]	2014	-	MOH and MOE	Schools	-	- A list of 24 food items prohibited from advertising in all media.
		-	-	-	Schools	-	- Ban the marketing of soft drinks, potato crisps and sweet biscuits in schools.
	WHO 2013 [2]	-	National	MOH	Kindergartens, primary and secondary school premises	-	Marketing of unhealthy foods like high-fat/energy and micronutrient-poor foods and beverages are not allowed on premises.
	Omidvar et al 2021 [11]	1978	National	Ministry of Culture, Islamic Guidance and MOE	Kindergartens and Schools (all children aged 2-18 years)	Ban on all marketing and advertising in kindergartens and schools.	**The ban law of food marketing and advertising in kindergartens and schools:** Regulatory measures for restriction of marketing and advertisements of unhealthy food and sweetened beverages in kindergartens and schools.
**Iraq**	WHO GINA [20, 21]	2018-2022	National	General Secretariat for the Council of Ministers (MOH; in collaboration	School students	Promote interventions to reduce the main shared modifiable risk factors for NCDs: tobacco use, unhealthy diet and physical inactivity.	National strategy for the prevention and control of non-communicable diseases 2018-2022:<br>- Identify food products and beverages marketed (those high in SFA, sugars and salt).

				with the MOE, MOF)			
**Jordan**	WHO EMRO 2018 [13]	-	-	-	Schools	-	Ban the marketing of soft drinks, potato crisps and sweet biscuits in schools.
	WHO 2018 [1]	1999	National	MOE and MOH	Kindergartens and schools	- Reduce or prevent child undernutrition (stunting, wasting, micronutrient deficiencies).<br>- Improve academic performance.<br>- Reduce food insecurity and hunger.	Standards for marketing of food and non-alcoholic beverages to children in school setting
**KSA**	WHO EMRO 2018 [13]	-	-	-	Schools	-	Ban the marketing of soft drinks, potato crisps and sweet biscuits in schools.
	WHO 2018 [1]	-	National	MOE and MOH	Kindergartens and schools	- Reduce or prevent child undernutrition (stunting, wasting, micronutrient deficiencies).<br>- Reduce or prevent childhood overweight or obesity.<br>- Foster healthy diet and lifestyle habits.<br>- Educate children and improve knowledge about healthy diet and lifestyle habits.<br>- Improve children's skills (e.g. cooking, food hygiene).<br>- Improve school enrolment.<br>- Improve school attendance.<br>- Improve academic performance.	Standards for marketing of food and non-alcoholic beverages to children in school setting
**Kuwait**	WHO EMRO 2018 [13]	-	-	-	Schools	-	Ban the marketing of soft drinks, potato crisps and sweet biscuits in schools.
	WHO 2018 [1]	-	National	MOE and MOH	Kindergartens and schools	- Reduce or prevent child undernutrition (stunting, wasting, micronutrient deficiencies).	Standards for marketing of food and non-alcoholic

						- Reduce or prevent childhood overweight or obesity.<br>- Foster healthy diet and lifestyle habits.<br>- Educate children and improve knowledge about healthy diet and lifestyle habits.<br>- Improve academic performance.	beverages to children in school setting.
	WHO 2013 [2]	-	National	MOH	Kindergartens, primary and secondary school premises	-	Marketing of unhealthy foods like high-fat/energy and micronutrient-poor foods and beverages are not allowed on premises.
**Lebanon**	WHO EMRO 2018 [13]	-	-	-	Schools	-	Ban the marketing of soft drinks, potato crisps and sweet biscuits in schools.
**Oman**	WHO 2018 [1]	1996	National	MOE and MOH	Kindergartens and schools	- Reduce or prevent child undernutrition (stunting, wasting, micronutrient deficiencies).<br>- Reduce or prevent childhood overweight or obesity.<br>- Foster healthy diet and lifestyle habits.<br>- Educate children and improve knowledge about healthy diet and lifestyle habits.<br>- Improve school attendance.<br>- Improve academic performance.	Standards for marketing of food and non-alcoholic beverages to children in school setting.
	WHO EMRO 2018 [13]	-	-	-	Schools	-	Ban the marketing of soft drinks, potato crisps and sweet biscuits in schools.

	WHO 2013 [2]	-	National	Schools and regional municipalities, schools' administrators, school health teams	Kindergartens and schools (primary and secondary schools)	-	Marketing of unhealthy foods like high-fat/energy and micronutrient-poor foods and beverages are not allowed on premises.
**Palestine**	WHO EMRO 2018 [13]	-	-	-	Schools	-	Ban the marketing of soft drinks, potato crisps and sweet biscuits in schools.
**Qatar**	WHO EMRO 2018 [13]	-	-	-	Schools	-	Ban the marketing of soft drinks, potato crisps and sweet biscuits in schools.

Abbreviations: ADEC: Abu Dhabi Education Council; AUB: American University of Beirut; DHA: Dubai Health Authority; EMRO: Regional Office for the Eastern Mediterranean; FNA: Food and Nutrition Authority; FZE: free zone establishments; GCC: Gulf Cooperation Council; GINA: Global Database on the Implementation of Nutrition Action; GSO: GCC Standardization Organization; HPS: health promoting schools; HSC: Healthy School Canteens; IHPS: Iranian health promoting schools; KSA: Kingdom of Saudi Arabia; MOE: Ministry of Education; MOEHE: Ministry of Education and Higher Education; MOF: Ministry of Finance; MOH: Ministry of Health; MOHAP: Ministry of Health and Prevention; MOHME: Ministry of Health and Medical Education; MOPH: Ministry of Public Health; MSG: monosodium glutamate; NFP: nutrition focal points; NGO: non-governmental organizations; QCC: Quality and Conformity Council; SFA: saturated fatty acids; TFA: trans fatty acids; UAE: United Arab Emirates; UHT: ultra high temperature; UNICEF: United Nations International Children's Emergency Fund; WFP: World Health programme; WHO: World Health Organization.

References

1. World Health Organization. *Global nutrition policy review 2016–2017: Country progress in creating enabling policy environments for promoting healthy diets and nutrition*; World Health Organization: Geneva, Switzerland, 2018; Available online: https://www.who.int/publications/i/item/9789241514873.
2. World Health Organization. *Global nutrition policy review: what does it take to scale up nutrition action?*; World Health Organization: Geneva, Switzerland, 2013; Available online: https://apps.who.int/iris/bitstream/handle/10665/84408/9789241505529_eng.pdf?sequence=1&isAllowed=y.
3. Ministry of Health and Prevention-UAE. *National Action Plan in Nutrition 2017-2021*; 2017; Available online: https://extranet.who.int/nutrition/gina/sites/default/filesstore/ARE%202017%20National%20Strategy%20Plan%20in%20Nutrition.pdf.
4. Garemo, M.; Elamin, A.; Van De Venter, A. A review of the nutritional guidelines for children at nurseries and schools in Middle Eastern countries. *Mediterr J Nutr Metab* **2019**, *12*, 255-70.
5. Public Health Directorate Bahrain. *Annual report*; Public Health Directorate: Manama, Bahrain, 2014; Available online: https://www.moh.gov.bh/Content/Files/Publications/X_635906124344978750.pdf.
6. Ministry of Health-Bahrain. MOH organize a workshop for school canteen operators in collaboration with school health section at the ministry of Education. Available online: https://www.moh.gov.bh/News/Details/3453?lang=en (accessed on 8 February 2023).
7. Aldinger, C. E.; Whitman, C. V. *Case studies in global school health promotion: from research to practice*; Springer Science and Business Media: New York, US, 2009.
8. AlMulla AlHarmasAlHajeri, M.; Al Thukair, L. A. A. A.; Sarhan, N. Bahrain: National Comprehensive School Health Program, Health-Promoting Schools. *Case Stud Glob Sch Health Promot: From Res Pract* **2009**, 239-49.
9. Ministry of Education and Technical Education-Egypt. The Ministers of "Health" and "Education" are discussing ways of cooperation to provide healthy meals for school students. Available online: https://moe.gov.eg/en/what-s-on/news/the-ministers-of-health-and-education/ (accessed on 16 February 2023).
10. Yazdi-Feyzabadi, V.; Rashidian, A.; Rarani, M. A. Socio-economic inequality in unhealthy snacks consumption among adolescent students in Iran: A concentration index decomposition analysis. *Public Health Nutr* **2019**, *22*, 2179-88.
11. Omidvar, N.; Babashahi, M.; Abdollahi, Z.; Al-Jawaldeh, A. Enabling food environment in kindergartens and schools in iran for promoting healthy diet: Is it on the right track? *Int J Environ Res Public Health* **2021**, *18*.
12. Sayyari, A. A.; Abdollahi, Z.; Ziaodini, H.; Olang, B.; Fallah, H.; Salehi, F.; Heidari-Beni, M.; Imanzadeh, F.; Abasalti, Z.; Fozouni, F. et al. Methodology of the comprehensive program on prevention and control of overweight and obesity in iranian children and adolescents: The Iran-ending childhood obesity (Iran-ECHO) program. *Int J Prev Med* **2017**, *8*.
13. World Health Organization Regional Office for the Eastern Mediterranean. *Implementing the WHO Recommendations on the Marketing of Food and Non-Alcoholic Beverages to Children in the Eastern Mediterranean Region*; World Health Organization: Cairo, Egypt, 2018; Available online: https://apps.who.int/iris/bitstream/handle/10665/328213/EMROPUB_2018_2248_en.pdf?sequence=1&isAllowed=y.
14. World Health Organization Regional Office for the Eastern Mediterranean. Nutrition: Success stories. Available online: http://www.emro.who.int/nutrition/resources/success-stories.html (accessed on 27 January 2023).

15. Ministry of Health and Medical Education. *National Guidelines for Healthy Nutrition Canteen in Schools*; Ministry of Health and Medical Education: Tehran, Iran, 2017; Available online:
16. Babashahi, M.; Omidvar, N.; Joulaei, H.; Zargaraan, A.; Zayeri, F.; Veisi, E.; Doustmohammadian, A.; Kelishadi, R. Scrutinize of healthy school canteen policy in Iran's primary schools: a mixed method study. *BMC Public Health* **2021**, *21*, 1566.
17. Feyzabadi, V. Y.; Mohammadi, N. K.; Omidvar, N.; Karimi-Shahanjarini, A.; Nedjat, S.; Rashidian, A. Factors associated with unhealthy snacks consumption among adolescents in Iran's schools. *International journal of health policy and management* **2017**, *6*, 519.
18. Al-Jawaldeh, A.; Hammerich, A.; Doggui, R.; Engesveen, K.; Lang, K.; McColl, K. Implementation of who recommended policies and interventions on healthy diet in the countries of the eastern mediterranean region: From policy to action. *Nutrients* **2020**, *12*, 1-19.
19. Sartipizadeh, M.; Yazdi-Feyzabadi, V.; Alipouri Sakha, M.; Zarrin, A.; Bazyar, M.; Zahirian Moghadam, T.; Zandian, H. Evaluating the Health Promoting Schools in Iran: Across-Sectional Study. *Health Education* **2021**, *121*, 125-39.
20. Supreme Committee for the Prevention and Control of Non-communicable Diseases; Ministry of Health-Iraq. *National strategy for the prevention and control of non-communicable diseases 2018-2022*; Baghdad, Iraq, 2018; Available online: https://extranet.who.int/nutrition/gina/en/node/40323.
21. Supreme Committee for the Prevention and Control of Non-communicable Diseases; Ministry of Health-Iraq. *The National Strategy for Prevention and Control of Noncommunicable Diseases* 2013; Available online: https://extranet.who.int/nutrition/gina/sites/default/filesstore/IRQ_The%20National%20Strategy%20for%20Prevention%20and%20Control%20of%20Noncommunicable%20Diseases-2013.pdf.
22. Ministry of Health-Iraq. *National Nutrition Strategy 2012-2021*; 2012; Available online: https://extranet.who.int/nutrition/gina/en/node/8387.
23. Ministry of Education-Jordan. *Health requirements for school canteens and foods allowed and prohibited to be sold for the year 2012*; 2012; Available online: https://extranet.who.int/nutrition/gina/en/node/66515.
24. Evans, C. E. L.; Albar, S. A.; Vargas-Garcia, E. J.; Xu, F. School-based interventions to reduce obesity risk in children in high- and middle-income countries. *Adv Food Nutr Res* **2015**, *76*, 29-77.
25. Ministry of Health-Jordan; WHO. *Nutrition in Jordan Update and plan of Action*; 2006; Available online: https://extranet.who.int/nutrition/gina/sites/default/filesstore/aNutrition%20in%20Jordan-Policy.pdf.
26. Ministry of Education-Kingdom of Saudi Arabia. *Health Requirements in the Provision of School Feeding Services*; 2022; Available online: https://moe.gov.sa/ar/education/generaleducation/Documents/%D8%AE%D8%AF%D9%85%D8%A7%D8%AA_%D8%A7%D9%84%D8%AA%D8%BA%D8%B0%D9%8A%D8%A9.pdf.
27. Al-Eid, A. J.; Al-Ahmed, Z. A.; Al-Omary, S. A.; Al-Harbi, S. M. RASHAKA program: a collaborative initiative between Ministry of Health and Ministry of Education to control childhood obesity in Saudi Arabia. *Saudi J Obes* **2017**, *5*, 22-27.
28. Ministry of Health-Kingdom of Saudi Arabia. School-Based Obesity Control (Rashaqa). Available online: https://www.moh.gov.sa/en/Ministry/Projects/agility/Pages/default.aspx (accessed on 16 February 2023).
29. Ministry of Health-KSA. *Obesity Control Program*; General Department for the Control of Genetic and Chronic Diseases: 2017; Available online: https://extranet.who.int/nutrition/gina/en/node/39427.

30. Editorial. *East Mediterr Health J* **2019**, *25*, 223-24.
31. Ministry of Health-Kuwait; Gulf Health Council; United Nations Development Programme; World Health Organization; Secretariat of the UN Inter-Agency Task Force on NCDS. *The Case for Investment in Prevention and Control of Non-Communicable Diseases in Kuwait*; Ministry of Health: Kuwait, 2021; Available online:
32. World Health Organization Regional Office for the Eastern Mediterranean. *Kuwait health profile 2015*; World Health Organization Regional Office for the Eastern Mediterranean: 2016; Available online: https://apps.who.int/iris/bitstream/handle/10665/253770/EMROPUB_2016_EN_19271.pdf?sequence=1&isAllowed=y.
33. Behbehani, K. Kuwait national programme for healthy living: First 5-year plan (2013-2017). *Med Princ Pract* **2014**, *23*, 32-42.
34. Ministry of Health-Morocco; UNICEF. *La Stratégie Nationale de la Nutrition*; 2011; Available online: https://extranet.who.int/nutrition/gina/en/node/17819.
35. Ministry of National education-Morocco. Nutrition education. Available online: https://www.men.gov.ma/Fr/Pages/Edunutri.aspx (accessed on 20 February 2023).
36. World Health Organization. *Health-promoting schools initiative in Oman. A WHO case study in intersectoral action*; World Health Organization: Cairo, Egypt, 2013; Available online: https://applications.emro.who.int/dsaf/EMROPUB_2013_EN_1587.pdf.
37. Government of Khyber Pakhtunkhwa. *Khyber Pakhtunkhwa Multi-sectoral Integrated Nutrition Strategy*; Planning and Development Department: 2014; Available online: https://extranet.who.int/nutrition/gina/sites/default/filesstore/PAK_2014_Khyber%20Pakhtunkhwa%20Integrated%20Nutrition%20Strategy.pdf.
38. Ministry of Education and Higher Education-Palestine. General Directorate of School Health. Available online: https://www.mohe.ps/home/%D8%A7%D9%84%D8%A5%D8%AF%D8%A7%D8%B1%D8%A9-%D8%A7%D9%84%D8%B9%D8%A7%D9%85%D8%A9-%D9%84%D9%84%D8%B5%D8%AD%D8%A9-%D8%A7%D9%84%D9%85%D8%AF%D8%B1%D8%B3%D9%8A%D8%A9/ (accessed on 23 February 2023).
39. Ministry of Education and Higher Education-Qatar. *Guidance for supervisors of school canteens for the academic year 2018-2019*; Health and Safety Department: 2018; Available online: https://www.edu.gov.qa/ar/Deputy/sharedservicesaffairs/healthandsafety-dept/Documents/FilesLibrary/2-GuidetoSchoolCafeteriaSupervisors.pdf.
40. Choudhury, S.; Omar, O.; Arora, T.; Rifai, N.; Chagoury, O. Qatar obesity reduction study (QORS): report on a pilot school-based nutrition education campaign in Qatar. *J Child Obes* **2018**, *2*, 007.
41. Ministry of Health-UAE. *Healthy Recipes for School Lunch Bag. MA'KOM for a Balanced Diet, 1st ed*; Health Education and Promotion Department: Dubai, United Arab Emirates, 2019; Available online:
42. Abdullatif, M.; AlAbady, K.; Altheeb, A.; Rishmawi, F.; Jaradat, H.; Farooq, S. Prevalence of Overweight, Obesity, and Dietary Behaviors among Adolescents in Dubai Schools: A Complex Design Survey 2019. *Dubai Med J* **2022**.
43. Ahmed, A. Junk food banned from every Dubai school canteen, The National [Internet]. Available online: https://www.thenationalnews.com/uae/junk-food-banned-from-every-dubai-school-canteen-1.365770 (accessed on 1 June 2023).
44. Khaleej Times. Food items banned in Dubai school canteens [Internet]. Available online: https://www.khaleejtimes.com/news/uae-health/11-food-items-banned-in-dubai-school-canteens?_refresh=true (accessed on 1 June 2023).

45. Algurg, R.; Mahfouz, N. A.; Otaki, F.; Alameddine, M. Towards the Upscaling of School Nutrition Programs in Dubai: an Exploratory Study (pre-print). *Res Sq* **2021**, *10*, 1-11.
46. AlGurg, R. S.; Mahfouz, N. H. A.; Otaki, F. Interviews with key informants to exploe school nutrition programs: a case-study in Dubai. *IA13-COVER* **2020**, 106.
47. Emirates 24/7. New school canteen rules for Abu Dhabi. Available online: https://www.emirates247.com/news/emirates/new-school-canteen-rules-for-abu-dhabi-2015-09-05-1.602379 (accessed on 2 June 2023).
48. Abu Dhabi Education Council. *ADEC Public Schools (P-12) Policy Manual* 2014; Available online: https://docplayer.net/25406908-Adec-public-schools-p-12-policy-manual-september-2014.html.
49. Habib-Mourad, C.; Hwalla, N.; Maliha, C.; Zahr, S.; Antoniades, K. Ajyal Salima a novel public–private partnership model for childhood obesity prevention in the Arab countries. *Front Public Health* **2022**, *10*.
50. Habib-Mourad, C.; Ghandour, L. A.; Moore, H. J.; Nabhani-Zeidan, M.; Adetayo, K.; Hwalla, N.; Summerbell, C. Promoting healthy eating and physical activity among school children: Findings from Health-E-PALS, the first pilot intervention from Lebanon. *BMC Public Health* **2014**, *14*, 940.
51. Ministry of Education-Jordan. Department of School Nutrition and Health. Available online: https://moe.gov.jo/node/21657 (accessed on 16 February 2023).
