# Supplementary material for: School-Based Nutrition Programs in the Eastern Mediterranean Region: A Systematic Review
Source: Int J Environ Res Public Health. 2023 Nov 10;20(22):7047. doi: 10.3390/ijerph20227047 (PMC10671197; doi:10.3390/ijerph20227047)
Supplement: Supplementary file 1 [file ijerph-20-07047-s001.zip › Table S7.pdf]

Table S7. Fruit, Vegetable and Milk Schemes in Countries of the EMR

Country	Reference	Year and Status	National or Regional	Leadership	Target Population	Objective	Brief Description of the Policy/Intervention
**School fruit and vegetable scheme**							
**Bahrain**	WHO GINA [1]	2012<br><br>Total duration: 4 years	National	MOH	School students	- Sustain the population's health through health promotion and prevention:<br>Promote healthy lifestyles to reduce non-communicable diseases.	- Increase the % of people consuming fruit and vegetables to 3-5 intakes everyday.
	WHO 2013 [2]	-	National	MOH	Kindergartens, primary and secondary schools	- Address obesity and diet-related NCDs.	- Provide fruits and vegetables.
**Iran**	Babashahi et al 2021 [3]; Omidvar et al 2021 [4]	2014	National	MOE and MOHME	School environment and canteens; students aged 7-18 years	- Increase access to healthy snacks.<br>- Prevent the supply of foods with low nutritional value.	Improve visibility and attractiveness of fruits and vegetables in school cafeterias using environmental nudges.
**Iraq**	WHO 2018 [5]	-	National	MOH and MOE	Kindergartens and schools	- Reduce or prevent child undernutrition (stunting, wasting, micronutrient deficiencies).<br>- Reduce or prevent childhood overweight or obesity.<br>- Foster healthy diet and lifestyle habits.<br>- Educate children and improve knowledge about healthy diet and lifestyle habits.<br>-Improve children's skills (e.g. cooking, food hygiene).<br>- Improve school enrolment.<br>- Improve school attendance.<br>- Improve academic performance.<br>- Tackle health inequalities.<br>- Reduce food insecurity and hunger.	Fruits and vegetables are part of the menu daily.

						- Support the agriculture sector by creating farm to school linkages (e.g. cereals, milk, fruit and vegetables supply).	
**Jordan**	Evans et al 2015 [6]	2012	National	MOH	Schools	- Reduce the intake of fat and sugar consumed by students.	**National food standard regulations as a school health strategy:**<br>- Offer fruit and vegetables for breakfast at the shops in schools.
	WHO 2018 [5]	1999	Regional	MOE and MOH	Kindergartens and schools	- Reduce or prevent child undernutrition (stunting, wasting, micronutrient deficiencies).<br>- Improve academic performance.<br>- Reduce food insecurity and hunger.	Fresh fruits and vegetables provided daily.
**KSA**	MOE [7]	2022	National	MOE and MOH	Schools	-	<u>Guidelines for the provision of school feeding services:</u><br>*Allowed foods:*<br>- Fresh and seasonal fruits and vegetables.<br>- Dried fruits, except those coated with sugar or other sweeteners.
	Al-Eid et al 2017 [8] and MOH 2017 [9]	2017-2020	National	MOH in cooperation with the MOE	Schools	- Increase awareness about the importance of a healthy lifestyle.<br>- Improve the school environment.<br>- Reduce the prevalence of obesity among school-aged children and adolescents in some selected schools at a rate of 5% by the end of 2020.<br>- Improve the nutritional behavior among students.	**The RASHAKA Initiative:** Provide fruits, vegetables in school cafeterias.

						- Provide the preventive and therapeutic services to overweight and obese students.	
**Kuwait**	Garemo et al 2019 [10]	**-**	-	MOE and MOH	School premises	Improve the health among children in Kuwait.	Promote increased intake of fruits and vegetables from the canteens during breakfast.
	WHO 2018 [5]	-	Regional	MOE and MOH	Kindergartens and schools	- Reduce or prevent child undernutrition (stunting, wasting, micronutrient deficiencies).<br>- Reduce or prevent childhood overweight or obesity.<br>- Foster healthy diet and lifestyle habits.<br>- Educate children and improve knowledge about healthy diet and lifestyle habits.<br>- Improve academic performance.	Fresh fruits and vegetables, dried fruits and 100% fruit juices are provided daily.
	WHO 2013 [2]	-	National	MOH	Kindergartens, primary and secondary schools	-	- Provide fruits and vegetables.
**Morocco**	WHO 2018 [5]	-	National	MOE and MOH in addition to WHO, UNICEF and WFP	Kindergartens and schools	- Foster healthy diet and lifestyle habits.<br>- Educate children and improve knowledge about healthy diet and lifestyle habits.<br>- Improve children's skills (e.g. cooking, food hygiene).<br>- Improve school enrolment.<br>- Improve school attendance.<br>- Improve academic performance.<br>- Tackle health inequalities.	Fruits and vegetables are part of the menu daily.

**Oman**	WHO 2013 [11]; Aldinger and Whitman 2009 [12]	2004 – 2009 (for four academic years)	Regional	WHO global initiative, with representation from MOH and MOE	All grades; implemented in 19 schools	- Create a healthier environment and lifestyle in schools and in society.<br>- Address many challenges, especially the unhealthy lifestyle emerging among school students.<br>- Raise health awareness of the students and their families, by provision of adequate knowledge of good healthy habits.<br>- Provide comprehensive health services that deal with the physical, mental, and social health needs and problems of this population.<br>- Ensure healthy school environments.	**HPS Initiative:**<br>Schools should emphasize changing the poor nutritional habits of students, especially those who fail to eat sufficient fruits and vegetables.
	WHO 2018 [5]; WHO 2013 [2]	1996	National	MOE and MOH, in collaboration with schools and regional muncipalities, schools' adminstrators, school health teams	Kindergartens and schools (primary and secondary schools)	- Reduce or prevent child undernutrition (stunting, wasting, micronutrient deficiencies).<br>- Reduce or prevent childhood overweight or obesity.<br>- Foster healthy diet and lifestyle habits.<br>- Educate children and improve knowledge about healthy diet and lifestyle habits.<br>- Improve school attendance.<br>- Improve academic performance.	- Provide fruits and vegetables.
**Pakistan**	WHO EMRO [13]	2020	National	Government of Pakistan	Schools	- Aim to address micronutrient malnutrition among Pakistani adolescents.	**Adolescent Nutrition Supplementation Guidelines:**<br>Improve intakes of fruits and vegetables.
	WHO GINA [14]	2014	Regional; Khyber Pakhtunk	Government - Department of Elementary and	School canteens	- Improve population nutrition wellbeing.	- Regulate schools’ canteen and vendors for nutritious and safe foods: Instructions to

				Secondary School Education		- Focus on remedial measures for addressing nutritional issues that have not only been adversely affecting the behvioural, cognitive, scholastic, physical performances but have also been increasing morbidity and mortality and impairing socioeconomic development.	schools for ensuring availability of fruits.
**Qatar**	Garemo et al 2019 [10]	-	-	MOE	Schools	- Support a healthy upbringing.<br>- Implement a national school snack program, and introduce material about nutrition to the school curriculum.	Increase the provision of fruits, vegetables and salads.
**Tunisia**	WHO GINA [15, 16]	2012 and 2015-2016	National	MOH	Kindergartens and primary schools	Reduce morbidity, disabilities and premature mortality related to NCDs and their risk factors:<br>- Strengthen the promotion of healthy lifestyles and the prevention of NCDs.<br>- Ensure quality management of NCDs<br>- Improve governance at all levels of competence and responsibility.<br>- Develop a monitoring and evaluation system for NCDs and their risk factors.	**Obesity Prevention and Control Strategy 2012; National Multisectoral Strategy for the Prevention and Control of Non-Communicable Diseases (NCD) 2018-2025:**<br>- Implement nutrition education in preschools and schools to help increase the consumption of fruits and vegetables.
	WHO 2013 [2]	-	National	-	Kindergartens, primary and secondary schools	**-**	- Provide fruits and vegetables.
**UAE**	WHO GINA [17]	2017-2021	National	MOHAP	School children	Improve the nutritional status of all population residing in the UAE with a collective vision of a healthier and sustainable future; guided by the international, regional and national	**National Action Plan in Nutrition 2017-2021:**<br>- Make available fruits and vegetables to school children.

						policies and strategies to promote health.<br><br>**Strategic objectives:**<br>- Reduce morbidity and mortality from NCDs by following healthy diet and physical activity, through achieving the following targets:<br>10% relative reduction in prevalence of insufficient physical activity.<br>30% relative reduction in mean population intake of salt.<br>Reduce the prevalence of obesity among children (5-17 years) by 2.4% (baseline 14.4% target 12%).	
	Abdullatif et al 2022 [18]; Ahmed 2011 [19]; Khaleej Times 2017 [20]	2011 and in 2017 an update of this policy was released	Regional; Dubai	Government (DHA)	School canteens	Improve health to prevent overweight and obesity among students.	-Encourage healthy food options, such as fresh fruits and vegetables.
**School milk scheme**							
**Bahrain**	WHO 2013 [2]	-	National	MOH	Kindergartens, primary and secondary schools	**-**	- Provide milk.
**Iran**	Omidvar et al 2021 [4]	2001	National	MOE; MOHME; Planning and Budget Organization; Ministry of Industry, Mine and Trade; and National	Students aged 4-18 years in schools	- Promote milk consumption.<br>- Improve health level in students.	**School milk program:**<br>- Direct provision of milk.

				Standardization Organization			
	WHO 2018 [5]	-	National	MOE and MOH	Kindergartens and schools	- Reduce or prevent child undernutrition (stunting, wasting, micronutrient deficiencies).<br>- Reduce or prevent childhood overweight or obesity.<br>- Foster healthy diet and lifestyle habits.<br>- Educate children and improve knowledge about healthy diet and lifestyle habits.<br>- Tackle health inequalities.<br>- Reduce food insecurity and hunger.	- Provide milk, including full-fat or whole milk.
**KSA**	MOE [7]	2022	National	MOE and MOH	Schools	-	Guidelines for the provision of school feeding services:<br>*Allowed foods:*<br>Milk: fresh or dry powder milk (UHT, full fat or low fat) and fortified with vitamin D and Calcium; milk with natural flavours;<br>low fat and low salt cheeses to be used;<br>sandwiches and pies that contain cheese, labneh.<br><br>*Restricted foods:*<br>Milk and zabadi with artificial flavours and colourings.
	WHO 2018 [5]	-	National	MOE and MOH	Kindergartens and schools	- Reduce or prevent child undernutrition (stunting, wasting, micronutrient deficiencies).	- Provide milk.

						- Reduce or prevent childhood overweight or obesity.<br>- Foster healthy diet and lifestyle habits.<br>- Educate children and improve knowledge about healthy diet and lifestyle habits.<br>- Improve children's skills (e.g. cooking, food hygiene).<br>- Improve school enrolment.<br>- Improve school attendance.<br>- Improve academic performance.	
**Kuwait**	Garemo et al 2019 [10]	**-**	-	MOE and MOH	School premises	Improve the health among children in Kuwait.	Promote increased intake of milk and milk products from the canteens during breakfast.
	WHO 2013 [2]	-	National	MOH	Kindergartens and schools	-	- Provide milk, including full-fat or whole milk.
**Morocco**	WHO 2018 [5]	-	National	MOE and MOH in addition to WHO, UNICEF and WFP	Kindergartens and schools	- Foster healthy diet and lifestyle habits.<br>- Educate children and improve knowledge about healthy diet and lifestyle habits.<br>- Improve children's skills (e.g. cooking, food hygiene).<br>- Improve school enrolment.<br>- Improve school attendance.<br>- Improve academic performance.<br>- Tackle health inequalities.	School milk scheme.
**Oman**	WHO 2018 [5]; WHO 2013 [2]	1996	National	MOE and MOH, in collaboration with schools and regional muncipalities, schools'	Kindergartens and schools (primary and secondary schools)	- Reduce or prevent child undernutrition (stunting, wasting, micronutrient deficiencies).<br>- Reduce or prevent childhood overweight or obesity.	- Provide milk.

				adminstrators, school health teams		- Foster healthy diet and lifestyle habits.<br>- Educate children and improve knowledge about healthy diet and lifestyle habits.<br>- Improve school attendance.<br>- Improve academic performance.	
**Pakistan**	WHO GINA [14]	2014	Regional; Khyber Pakhtunk	Government - Department of Elementary and Secondary School Education	Schools	- Improve population nutrition wellbeing.<br>- Focus on remedial measures for addressing nutritional issues that have not only been adversely affecting the behavioral, cognitive, scholastic, physical performances but have also been increasing morbidity and mortality and impairing socioeconomic development.	- School milk/food/fortified food and vitamin supplement programme for malnourished children especially middle/high school girls (adolescents).<br>- Regulate schools' canteen and vendors for nutritious and safe foods: Instructions to schools for ensuring availability of milk.
**Qatar**	Garemo et al 2019 [10]	-	-	MOE	Schools	- Support a healthy upbringing.<br>- Implement a national school snack program, and introduce material about nutrition to the school curriculum.	Increase the provision of milk and dairy products.
**Tunisia**	WHO 2013 [2]	-	National	-	Kindergartens, primary and secondary schools	**-**	- Provide milk.

Abbreviation: DHA: Dubai Health Authority; EMRO: Regional Office for the Eastern Mediterranean; GINA: Global Database on the Implementation of Nutrition Action; HPS: health promoting schools; KSA: Kingdom of Saudi Arabia; MOE: Ministry of Education; MOH: Ministry of Health; MOHAP: Ministry of Health and Prevention; MOHME: Ministry of Health and Medical Education; NCD: non-communicable diseases; UAE: United Arab Emirates; UHT: ultra high temperature; UNICEF: United Nations International Children's Emergency Fund; WFP: World Health programme; WHO: World Health Organization.

References

1. Ministry of Health-Bahrain. *Bahrain's Health Agenda. Health Improvement Strategy*; Directorate of Health Planning: 2012; Available online: https://extranet.who.int/nutrition/gina/sites/default/filesstore/BHR%202012%20%20Health%20Improvement%20Strategy.pdf.
2. World Health Organization. *Global nutrition policy review: what does it take to scale up nutrition action?*; World Health Organization: Geneva, Switzerland, 2013; Available online: https://apps.who.int/iris/bitstream/handle/10665/84408/9789241505529_eng.pdf?sequence=1&isAllowed=y.
3. Babashahi, M.; Omidvar, N.; Joulaei, H.; Zargaraan, A.; Zayeri, F.; Veisi, E.; Doustmohammadian, A.; Kelishadi, R. Scrutinize of healthy school canteen policy in Iran's primary schools: a mixed method study. *BMC Public Health* **2021,** *21*.
4. Omidvar, N.; Babashahi, M.; Abdollahi, Z.; Al-Jawaldeh, A. Enabling food environment in kindergartens and schools in iran for promoting healthy diet: Is it on the right track? *International Journal of Environmental Research and Public Health* **2021,** *18*.
5. World Health Organization. *Global nutrition policy review 2016–2017: Country progress in creating enabling policy environments for promoting healthy diets and nutrition*; World Health Organization: Geneva, Switzerland, 2018; Available online: https://www.who.int/publications/i/item/9789241514873.
6. Evans, C. E. L.; Albar, S. A.; Vargas-Garcia, E. J.; Xu, F. School-Based Interventions to Reduce Obesity Risk in Children in High- and Middle-Income Countries. *Advances in Food and Nutrition Research* **2015,** *76*, 29-77.
7. Ministry of Education-Kingdom of Saudi Arabia. *Health Requirements in the Provision of School Feeding Services*; 2022; Available online: https://moe.gov.sa/ar/education/generaleducation/Documents/%D8%AE%D8%AF%D9%85%D8%A7%D8%AA_%D8%A7%D9%84%D8%AA%D8%BA%D8%B0%D9%8A%D8%A9.pdf.
8. Al-Eid, A. J.; Al-Ahmed, Z. A.; Al-Omary, S. A.; Al-Harbi, S. M. RASHAKA program: a collaborative initiative between Ministry of Health and Ministry of Education to control childhood obesity in Saudi Arabia. *Saudi Journal of Obesity* **2017,** *5*, 22-27.
9. Ministry of Health-Kingdom of Saudi Arabia. School-Based Obesity Control (Rashaqa). Available online: https://www.moh.gov.sa/en/Ministry/Projects/agility/Pages/default.aspx (accessed on 16 February 2023).
10. Garemo, M.; Elamin, A.; Van De Venter, A. A review of the nutritional guidelines for children at nurseries and schools in Middle Eastern countries. *Mediterranean Journal of Nutrition and Metabolism* **2019,** *12*, 255-270.
11. World Health Organization. *Health-promoting schools initiative in Oman. A WHO case study in intersectoral action*; World Health Organization: Cairo, Egypt, 2013; Available online: https://applications.emro.who.int/dsaf/EMROPUB_2013_EN_1587.pdf.
12. Aldinger, C.; Whitman, C. V. *Case studies in global school health promotion: from research to practice*; Springer: New York, US, 2009.
13. World Health Organization Regional Office for the Eastern Mediterranean. Nutrition: Success stories. Available online: http://www.emro.who.int/nutrition/resources/success-stories.html (accessed on 27 January 2023).
14. Government of Khyber Pakhtunkhwa. *Khyber Pakhtunkhwa Multi-sectoral Integrated Nutrition Strategy*; Planning and Development Department: 2014; Available online: https://extranet.who.int/nutrition/gina/sites/default/filesstore/PAK_2014_Khyber%20Pakhtunkhwa%20Integrated%20Nutrition%20Strategy.pdf.

15. Ministry of Health-Republic of Tunisia. *National Multisectoral Strategy for the Prevention and Control of Non-Communicable Diseases (NCD) 2018-2025*; 2018; Available online: https://extranet.who.int/nutrition/gina/sites/default/filesstore/TUN%202018%20Strat%C3%A9gie%20Nationale%20MNT.pdf.
16. Ministry of Health-Republic of Tunisia. *Obesity Prevention and Control Strategy*; 2012; Available online: https://extranet.who.int/nutrition/gina/sites/default/filesstore/TUN_2014_SPLO.pdf.
17. Ministry of Health and Prevention-UAE. *National Action Plan in Nutrition 2017-2021*; 2017; Available online: https://extranet.who.int/nutrition/gina/sites/default/filesstore/ARE%202017%20National%20Strategy%20Plan%20in%20Nutrition.pdf.
18. Abdullatif, M.; AlAbady, K.; Altheeb, A.; Rishmawi, F.; Jaradat, H.; Farooq, S. Prevalence of Overweight, Obesity, and Dietary Behaviors among Adolescents in Dubai Schools: A Complex Design Survey 2019. *DUBAI MEDICAL JOURNAL* **2022**.
19. Ahmed, A. Junk food banned from every Dubai school canteen, The National [Internet]. Available online: https://www.thenationalnews.com/uae/junk-food-banned-from-every-dubai-school-canteen-1.365770 (accessed on 1 June 2023).
20. Khaleej Times. Food items banned in Dubai school canteens [Internet]. Available online: https://www.khaleejtimes.com/news/uae-health/11-food-items-banned-in-dubai-school-canteens?_refresh=true (accessed on 1 June 2023).
