# Supplementary material for: School-Based Nutrition Programs in the Eastern Mediterranean Region: A Systematic Review
Source: Int J Environ Res Public Health. 2023 Nov 10;20(22):7047. doi: 10.3390/ijerph20227047 (PMC10671197; doi:10.3390/ijerph20227047)
Supplement: Supplementary file 1 [file ijerph-20-07047-s001.zip › Table S8.pdf]

Table S8. Programs on School Hygienic Cooking Facilities, Clean Environments and School Gardens in Countries of the EMR

Country	Reference	Year and Status	National or Regional	Leadership	Target Population	Objective	Brief Description of the Policy/Intervention
**School gardens**							
**Afghanistan**	MOPH 2010 [1]	2002	National	Public Nutrition Department<br><br>MAIL and MOE	Schools	- Promote healthy nutrition practices.<br>- Fight all types of malnutrition, including chronic and acute malnutrition, micronutrient deficiency diseases, and over-nutrition.<br>- Reduce child mortality.	School garden project.
**Jordan**	WHO 2018 [2]	1999	National	MOE and MOH	Kindergartens and schools	- Reduce or prevent child undernutrition (stunting, wasting, micronutrient deficiencies).<br>- Improve academic performance.<br>- Reduce food insecurity and hunger.	School gardening.
**Morocco**	WHO 2018 [2]	-	Regional	MOE and MOH in addition to WHO, UNICEF and WFP	Kindergartens and schools	- Foster healthy diet and lifestyle habits.<br>- Educate children and improve knowledge about healthy diet and lifestyle habits.<br>- Improve children's skills (e.g. cooking, food hygiene).<br>- Improve school enrolment.	School gardening activities in some schools.

						- Improve school attendance.<br>- Improve academic performance.<br>- Tackle health inequalities.	
**Palestine**	Bajraktarevic et al 2021 [3]; WHO 2021 [4]	2018	National	UNICEF-supported intervention; supporting MOE and MOH	School-aged children	- Establish healthy dietary and physical activity habits and improve the nutritional status of school-age children.<br>- Strengthen the involvement of parents, families and communities, complementing formal ongoing school interventions and creating an enabling environment for sustainable positive change around nutrition and healthy lifestyles.	Develop school gardens and plant vegetables for consumption by children.
**Sudan**	WHO GINA [5, 6]	2009-2012	National	MOH in collaboration with the Child and Adolescent Health Directorate, Ministry of Agriculture and Forestry, MOE, School Gardening and Nutrition Education Department and WFP	Schools	- Ensure the prevention and treatment of nutrition related disorders in emergency and non-emergency situations.<br>- Reduce nutritional risk for individuals throughout their life-cycle.<br>- Reduce nutrition risk and improve malnutrition prevention and treatment programming.	**National Nutrition Policy and Key Strategies 2009 and 2008-2012:**<br>- Establish school gardens linked to nutrition education.

						- Increased knowledge and awareness and improved nutrition practice at community level.<br>- Multi-sectoral coordination and collaboration to address malnutrition comprehensively and effectively, to bring about sustained change in population nutrition status.	
**Tunisia**	WHO 2018 [2]	-	National	MOE and WFP	Kindergartens and schools	- Reduce or prevent child undernutrition (stunting, wasting, micronutrient deficiencies).<br>- Reduce or prevent childhood overweight or obesity.<br>- Foster healthy diet and lifestyle habits<br>- Educate children and improve knowledge about healthy diet and lifestyle habits.<br>- Improve children's skills (e.g. cooking, food hygiene).<br>- Improve school enrolment.<br>- Improve school attendance.	School gardening being optional based on space and teaching practices.

						- Improve academic performance.<br>- Tackle health inequalities.<br>- Reduce food insecurity and hunger.	
**Hygienic cooking facilities and clean eating environment in schools**							
**Afghanistan**	Hees and Sankei 2013 [7]	2009-2010	Regional (8 provinces)	WFP's Afghanistan Protracted Relief Operation (under Food and Education)	Students in grades one through nine. 54 schools in 15 food-insecure districts across eight provinces, targeting 46,000 children	- Increase access to education for girls and boys in World Food Programme (WFP)-assisted schools.<br>- Address micronutrient deficiencies through the provision of food fortified with micronutrient powder (MNP) among 46,000 students in grades one through nine.	Construct efficient and hygienic school kitchens and canteens – including repair or construction work on kitchens and food stores.
**Bahrain**	Garemo et al 2019 [8]	-	-	-	School premises	-	Clean eating environment and hygienic cooking facilities.<br><br>The Bahraini government has endorsed the Hygiene Conditions for School Canteens and Handled Food (GSO 1971/2014). These standards, issued by GCC countries, include guidelines on food storage, content, hygiene and safety.

	WHO 2013 [9]	-	National	MOH	Kindergartens, primary and secondary schools	-	Hygienic cooking facilities and clean eating environment.
	Aldinger and Whitman 2009 [10]; AlMulla AlHarmasAlHajeri et al 2009 [11]	2004	Regional; Muharraq governorate	MOH in collaboration with the MOE, GCC and WHO EMRO	School environment	- Provide instruction to develop the knowledge, skills, attitudes, and behaviors related to healthy living.<br>- Support the provision of support services for students and their families.<br>- Create a healthy social and physical environment within the school.<br>- Integrate the concepts of personal health management, health promotion, and education.<br>- Incorporate strategies that are comprehensive, interdisciplinary, and outcome based.<br>- Be taught by teachers who are competent and qualified in health education and promotion.<br>- Provide sufficient instruction time to elicit behavior change.	**HPS program:**<br>Healthy school environment, and Nutrition and food safety are two key program elements.
**Iran**	Garemo et al 2019 [8]	-	-	-	Schools	-	Guidelines provide details on how food preparation and storage should occur.

	Sartipizadeh et al 2021 [12]; Yazdi-Feyzabadi et al 2018 [13]	2010 Piloted	5 provinces	MOE and MOHME	School environment and cafeteria	- Promote a healthy school food environment (compliance with healthy food canteen bylaw) combined with health education and improved eating behaviors in adolescents.<br>- Develop concepts of self-care and health promotion in both individual and society or community aspects and as an integrated and coordinated system for school health programs.<br>**-** Improve high-risk behaviours of adolescents.	**Iranian health promoting schools (IHPSs) program:**<br>- Supervise the way of preserving, distributing and consuming the food stuff at school.<br>- Have the health authenticity certificate and work permission of the base person in charge and the food stuff salesperson.
	WHO 2013 [9]	-	National	MOH	Kindergartens, primary and secondary schools	**-**	Hygienic cooking facilities and clean eating environment
**Iraq**	WHO 2013 [9]	-	National	-	Kindergartens and schools	**-**	Hygienic cooking facilities and clean eating environment
**KSA**	Garemo et al 2019 [8]	-	-	-	School premises	-	KSA endorsed the Hygiene Conditions for School Canteens and Handled Food (GSO 1971/2014). These standards, issued by GCC countries, include guidelines on food storage, content, hygiene and safety.

**Kuwait**	WHO 2013 [9]	-	National	MOH	Kindergartens, primary and secondary schools	-	Hygienic cooking facilities and clean eating environment
**Lebanon**	Garemo et al 2019 [8]	-	-	MOPH	Nurseries	-	National Guidelines for Early Childhood Care provide clear instructions on the preparation and storage of food in nursery kitchens.
**Oman**	Garemo et al 2019 [8]	-	-	-	School premises	-	Oman has endorsed the Hygiene Conditions for School Canteens and Handled Food, which focuses on food safety aspects.
	WHO 2013 [9]	-	National	Schools and regional muncipalities, schools' adminstrators, school health teams	Kindergartens and schools (primary and secondary schools)	-	Hygienic cooking facilities and clean eating environment
**Palestine**	Bajraktarevic et al 2021 [3]; WHO 2021 [4]	2018	National	UNICEF-supported intervention; supporting MOE and MOH	Schools	- Establish healthy dietary and physical activity habits and improve the nutritional status of school-age children.<br>- Strengthen the involvement of parents, families and communities, complementing formal ongoing school interventions and creating an enabling environment for sustainable positive	**Nutrition Friendly Schools Initiative:**<br>- Refurbish 10 schools to improve the water and sanitation facilities as part of the enabling environment interventions.<br>- Provide canteen equipment and hygiene materials.

						change around nutrition and healthy lifestyles.	
**Qatar**	Garemo et al 2019 [8]	2007	-	-	Preschoolers aged 3-6 years; child-care facilities	-	**Healthy and safety standards:**<br>- Food should be prepared and stored in a hygienic manner.
**Tunisia**	WHO 2013 [9]	-	National	-	Kindergartens, primary and secondary schools	-	Hygienic cooking facilities and clean eating environment

Abbreviations: EMRO: Regional Office for the Eastern Mediterranean; GCC: Gulf Cooperation Council; GINA: Global Database on the Implementation of Nutrition Action; GSO: GCC Standardization Organization; HPS: health promoting schools; IHPS: Iranian health promoting schools; KSA: Kingdom of Saudi Arabia; MAIL: Ministry of Agriculture, Irrigation and Livestock; MNPs: micronutrient powders; MOE: Ministry of Education; MOH: Ministry of Health; MOHME: Ministry of Health and Medical Education; MOPH: Ministry of Public Health; UNICEF: United Nations International Children's Emergency Fund; WHO: World Health Organization; WFP: World Food Programme.

References

1. Ministry of Public Health-Islamic Republic of Afghanistan. *National Public Nutrition Policy & Strategy 1388 – 1392 (2009-2013)*; Ministry of Public Health: 2010; Available online: https://extranet.who.int/nutrition/gina/sites/default/filesstore/AFG%202009%20National%20Public%20Nutrition%20Policy%20and%20Strategy.pdf.
2. World Health Organization. *Global nutrition policy review 2016–2017: Country progress in creating enabling policy environments for promoting healthy diets and nutrition*; World Health Organization: Geneva, Switzerland, 2018; Available online: https://www.who.int/publications/i/item/9789241514873.
3. Bajraktarevic, S.; Qadi, K.; Jouda, A.; Awadallah, Y.; Abueita, R. Improving the nutritional well-being of school-age children through the nutrition-friendly schools initiative (NFSI) in the State of Palestine. *Field Exchange - Emergency Nutrition Network ENN* **2021,** 47-50.
4. World Health Organization. *Nutrition action in schools: a review of evidence related to the nutrition-friendly schools initiative*; World Health Organization: Geneva, Switzerland, 2021; Available online: https://www.who.int/publications/i/item/9789241516969.
5. Federal Ministry of Health-Republic of Sudan. *National Nutrition Policy and Key Strategies*; Maternal and Child Health Directorate: 2009; Available online: http://www.fmoh.gov.sd/Health-policy/nationalnutritionpolicy.pdf.
6. Federal Ministry of Health-Republic of Sudan. *National Nutrition Strategy & Key Strategies (2008 - 2012)*; Maternal and Child Health Directorate: 2008; Available online: https://extranet.who.int/nutrition/gina/sites/default/filesstore/SDN%202008_National%20Nutrition%20Policy.pdf.
7. Hees, J. v.; Sankei, K. Home fortification in school feeding. Basel: Sight and Life; 2013. p. 39-41.
8. Garemo, M.; Elamin, A.; Van De Venter, A. A review of the nutritional guidelines for children at nurseries and schools in Middle Eastern countries. *Mediterr J Nutr Metab* **2019,** *12*, 255-270.
9. World Health Organization. *Global nutrition policy review: what does it take to scale up nutrition action?*; World Health Organization: Geneva, Switzerland, 2013; Available online: https://apps.who.int/iris/bitstream/handle/10665/84408/9789241505529_eng.pdf?sequence=1&isAllowed=y.
10. Aldinger, C.; Whitman, C. V. *Case studies in global school health promotion: from research to practice*; Springer: New York, US, 2009.
11. AlMulla AlHarmasAlHajeri, M.; Al Thukair, L. A. A. A.; Sarhan, N. Bahrain: National Comprehensive School Health Program, Health-Promoting Schools. *Case Studies in Global School Health Promotion: From Research to Practice* **2009,** 239-249.
12. Sartipizadeh, M.; Yazdi-Feyzabadi, V.; Alipouri Sakha, M.; Zarrin, A.; Bazyar, M.; Zahirian Moghadam, T.; Zandian, H. Evaluating the Health Promoting Schools in Iran: Across-Sectional Study. *Health Education* **2021,** *121*, 125-139.
13. Yazdi-Feyzabadi, V.; Omidvar, N.; Mohammadi, N. K.; Nedjat, S.; Karimi-Shahanjarini, A.; Rashidian, A. Is an Iranian health promoting school status associated with improving school food environment and snacking behaviors in adolescents? *Health Promotion International* **2018,** *33*, 1010-1021.
