# Supplementary material for: School-Based Nutrition Programs in the Eastern Mediterranean Region: A Systematic Review
Source: Int J Environ Res Public Health. 2023 Nov 10;20(22):7047. doi: 10.3390/ijerph20227047 (PMC10671197; doi:10.3390/ijerph20227047)
Supplement: Supplementary file 1 [file ijerph-20-07047-s001.zip › Table S9.pdf]

Table S9. Monitoring and Process Evaluation of School-based Programs in Countries of the EMR

Country	Reference	Year	National or Regional	Leadership and Target	Program/ Intervention	Monitoring/Evaluation Process and/or indicators
Afghanistan	Hees and Sankei 2013 [1]	2009-2010	Regional (8 provinces)	WFP's Afghanistan Protracted Relief Operation (under Food and Education).<br><br>**Target**: Students in grades 1-9	Meal fortification with MNPs.	Number of children receiving MNP fortified meals, the number of feeding days versus school days, and the number of days MNP was used.
	MOPH 2010 [2]	2002	National	Public Nutrition Department.<br>MAIL, and MOE<br><br>**Target**: Schools	School gardens project	Number of schools with school gardens available; Number of children participating in school gardening recreational activities.
Bahrain	Aldinger and Whitman 2009 [3]; AlMulla AlHarmasAlHajeri et al 2009 [4]	2004	Regional; Muharraq governorate	MOH in collaboration with the MOE, GCC and WHO EMRO<br><br>**Target**: School staff and administrators	HPS program	Coordinators visited the schools to review the implementation of the action plan and discuss the difficulties and opportunities for action.<br>Auditing of the program before, during, and after the implementation, in collaboration with the research committee in the MOE.
Iran	Sartipizadeh et al 2021 [5]; Yazdi-Feyzabadi et al 2018 [6]	2010 Piloted	5 provinces; plan to increase number of schools nationwide	MOE and MOHME<br><br>**Target**: Schools	Iranian health promoting schools (IHPSs) program.	A coordination team including the school principal and staff conducts an internal audit based on an internal audit checklist.<br>The results will be transferred to the County Committee for an external audit, using a checklist. This "External Audit Checklist" is prepared by the MOHME.<br><br>A team of health experts from a district health center of the primary health care (PHC) system run a two-stage external audit by visiting schools,

						observation and interviewing with students, staff and teachers.
	Babashahi et al 2021 [7]	2014	National	MOE and MOHME<br><br>**Target**: School environment and canteens; students aged 7-18 years	Healthy schools canteen bylaw	Separate supervisory checklists used by MOE and MOHME.<br>Various supervisory groups from the Student Organization, the Office of Health in the Department of Education, the Nutrition and Environmental Health Unit of Community Health centers, monitor the implementation process of the policy. Several committees at the MOHME or M0H and provincial committees are set up to review and coordinate policy implementation.
	Kheirouri 2014 [8]	-	National	Directory of Student Health in the MOE; Directory of Population, Family and School Health in the MOHME<br><br>**Target**: Adolescent girls attending public or private senior high schools in rural and urban areas	National iron supplementation program in 2001	Three separate process evaluation assessment tools were developed:<br>1) Collection of students' opinions on the program<br>2) Collection of school administrator opinions on the program<br>3) Collection of teacher opinions on the program
Iraq	WHO GINA [9]	2012-2021	National	General Secretariat for the Council of Ministers<br><br>**Target:** Schools, students and their canteens	National Nutrition Strategy 2012-2021	Establishing a high level committee to identify gaps in the nutrition curriculum.
KSA	Al-Eid et al 2017 and MOH [10, 11]	2017-2020	National	MOH in cooperation with the MOE<br><br>**Target**: School-aged students	The RASHAKA Initiative	Provision: number of primary care centers included in the initiative in the region; number of teachers, physicians, nurses, nutrition specialists, and health counselors in primary healthcare centers; number of education campaigns conducted in a year; the number of schools that carry out the initiative; and the number of training

						sessions per month given to primary healthcare workers and teachers.<br><br>Utilization: how many schools conduct RSHAKA Initiative activities, and how many school cafeterias follow the rules and meet the standards established by them.<br><br>Coverage: how many students have been screened, the percentage of obese students, and how many have been referred for medical care.<br><br>Impact: the number of students who eat healthy food, and decrease their weight.
Kuwait	Behbehani 2014 [12]; Evans et al 2015 [13]	2013-2017	National	MOE; MOH; Private School Council; Ministry of Commerce; Kuwait Counsellor Network; Ministry of Labour and Social Affairs; Council of Religious Affairs<br><br>**Targe**t: Schools and their staff	The Kuwait National Programme for Healthy Living	Schools will liaise with food-regulating bodies to evaluate the nutritional value of the food currently being provided in schools.<br>Impact assessment in schools: monitoring and recording anthropometric measurements in youth aged 5–18 years by the school nurse at regular intervals during the school year to understand whether any interventions have had a significant effect on reducing obesity in schools.
Lebanon	Habib-Mourad et al 2022 [14]	2010<br><br>2014: adoption by the MOEHE<br><br>(3-4 months	National	MOEHE; AUB; Nestlé Middle East FZE;<br><br>**Target**: School teachers and health educators, parents, school shops and environment	Ajyal Salima	Pre-test 1 week prior to the start of the intervention and a post-test 1 week after the completion of the program (3–4 months duration) in all schools.

		duration, each)				
	Jamaluddine et al 2022 [15]	2016	National	WFP<br><br>**Target:** Lebanese and Syrian refugee children attending primary public schools (Grades 1–6) in vulnerable communities	An emergency school feeding programme (SFP)	Regular post-distribution monitoring
Morocco	Hanel et al 2021 [16]; WFP 2016 [17]	2013-2017<br><br>Endorsed by the governmen t by July 2016	National (70% in rural areas)	WFP in collaboration with the government (Ministry of National Education and the Ministry of Agriculture); National School Meals Steering Committee<br><br>**Target**: Primary schools	National School Meals Programme	In-depth assessment of the National School Meals Programme, which took place in 2015.<br>Findings and the lessons learned were incorporated into the Plan of Action for the enhancement of the national school meals programme (2016-2018).<br><br>*Recommendations:*<br>Decentralize school meals management and transfer some responsibilities to the regional level.<br>Strengthen the program's monitoring and management systems.<br>Increase capacity of stakeholders through education and training of staff.<br>Improve supply chains for school meals.<br>Develop guidelines for school meals management, nutritional guidelines and nutritious menus.
Oman	WHO 2013 [18]; Aldinger and Whitman 2009 [3]	2004 – 2009	Regional	WHO global initiative, with representation from MOH and MOE<br><br>**Target**: All grades; implemented in 19 schools	HPS Initiative	Three levels of assessment:<br>1. Self-assessment: Conducted at the school level. (continuous process that helps the schools to measure. their achievements and update their activities accordingly.<br>2. Regional assessment: Conducted by the regional teams to determine which schools should get bronze or silver certificates.

						3. National assessment: Conducted by the national team to determine which schools have fulfilled the criteria of golden level.
Pakistan	WHO GINA [19]	2014	Regional; Khyber Pakhtunk	Government - Department of Elementary and Secondary School Education<br><br>**Target:** Schools and canteens	School health and nutrition assessment programme.	- Periodical visits to a selected district and schools to evaluate success and impediments in implementation of the programme.<br>- Qualitative and quantitative output indicators shall be set to measure the efficiency and effectiveness of the programme.
Palestine	Bajraktarevic et al 2021 [20]; WHO 2021 [21]	2018-2021	National	UNICEF-supported intervention; supporting MOE and MOH<br><br>**Target:** Students; school staff and administrators, parents, families and communities	Nutrition Friendly Schools Initiative	Generating evidence and measuring the results of the innovative NFSI approach and impact on the nutritional status of school-aged children.
Qatar	WHO GINA [22]	2018-2019	National	MOEHE; MOH<br><br>**Target**: School canteens	Guidance for supervisors of school canteens for the academic year 2018-2019	- Daily supervision of the canteen and monitoring the extent of compliance of the involved personnel.<br>- Implementing fines and penalties based on the nature of the violation.
	WHO GINA [23]	2017	National	MOEHE<br><br>**Target**: Schools	Food safety and health guide	- Evaluate the students' health nutrition programmes and activities and measure their impact on their health.<br>- Monitor and evaluate breakfast and food meals provided in schools or sent from home.
	WHO GINA [24]	2011-2016; adopted	National	MOPH<br><br>**Target:** Schools	National Nutrition and Physical Activity Action Plan 2011-2016	- Number of schools participating in the national school snack program out of the total number targeted.

UAE	WHO GINA - [25]	2017-2021	National	MOHAP<br><br>**Target:** Schools and their environment	National Action Plan in Nutrition 2017-2021	- Number of schools that have developed and implemented policy on healthy diet.<br>- Number of schools that have regulated availability of food in the school environment i.e. number of schools that have developed and implemented regulatory measures for marketing of unhealthy foods to children and adolescents.<br>- Number of schools that have made fruits and vegetables available to students.<br>- Number of schools that have incorporated and implemented nutrition education and activities in the school curriculum.<br>- Number of published evidence on health and nutrition status of school children.
	Aldinger and Whitman 2009 [3]	2004	National	MOH<br><br>**Target:** Students;<br>More than 17,000 students have therefore benefited from the project in 46 schools	HPS project	- Monitoring and evaluation were undertaken through school visits, meetings, and documentation of the outcomes. Health-related school activities were assessed by process and outcome evaluation.<br>-Process evaluation focused on level of awareness about the HPS concepts, documentation of the directives, minutes for the meeting, reviewing the framework of the work plans<br>-Outcome evaluation focused on comparing the program results to the baseline data and the set objectives of the project or the program.
Several countries: Lebanon, Jordan, Palestine, Bahrain, KSA and UAE	Habib-Mourad et al 2022 [14]	2018	-	MOE; MOH; Nestlé Middle East FZE; AUB<br><br>**Target:** Schools, canteens and teachers	Ajyal salima	A pre-test 1 week prior to the start of the intervention and a post-test 1 week after the completion of the program (3–4 months duration) in all schools.<br>Evaluation and monitoring involved all partners and stakeholders to ensure program sustainability and success.

Abbreviation: AUB: American University of Beirut; EMRO: Regional Office for the Eastern Mediterranean; FZE: free zone establishments; GCC: Gulf Cooperation Council; GINA: Global Database on the Implementation of Nutrition Action; HPS: health promoting schools; IHPS: Iranian health promoting schools; KSA: Kingdom of Saudi Arabia; MAIL: Ministry of Agriculture, Irrigation and Livestock; MOE: Ministry of Education; MOEHE: Ministry of Education and Higher Education; MOH: Ministry of Health; MOHAP: Ministry of Health and Prevention; MOHME: Ministry of Health and Medical Education; MOPH: Ministry of Public Health; MNP: micronutrient powders; NFSI: nutrition friendly schools initiative; PHC: primary health care; SFP: school feeding programme; UAE: United Arab Emirates; ; UNICEF: United Nations International Children's Emergency Fund;WFP: World Food Programme; WHO: World Health Organization.

References

1. Hees, J. v.; Sankei, K. Home fortification in school feeding. Basel: Sight and Life; 2013. p. 39-41.
2. Ministry of Public Health-Islamic Republic of Afghanistan. *National Public Nutrition Policy & Strategy 1388 – 1392 (2009-2013)*; Ministry of Public Health: 2010; Available online: https://extranet.who.int/nutrition/gina/sites/default/filesstore/AFG%202009%20National%20Public%20Nutrition%20Policy%20and%20Strategy.pdf.
3. Aldinger, C.; Whitman, C. V. *Case studies in global school health promotion: from research to practice*; Springer: New York, US, 2009.
4. AlMulla AlHarmasAlHajeri, M.; Al Thukair, L. A. A. A.; Sarhan, N. Bahrain: National Comprehensive School Health Program, Health-Promoting Schools. *Case Studies in Global School Health Promotion: From Research to Practice* **2009,** 239-249.
5. Sartipizadeh, M.; Yazdi-Feyzabadi, V.; Alipouri Sakha, M.; Zarrin, A.; Bazyar, M.; Zahirian Moghadam, T.; Zandian, H. Evaluating the Health Promoting Schools in Iran: Across-Sectional Study. *Health Education* **2021,** *121*, 125-139.
6. Yazdi-Feyzabadi, V.; Omidvar, N.; Mohammadi, N. K.; Nedjat, S.; Karimi-Shahanjarini, A.; Rashidian, A. Is an Iranian health promoting school status associated with improving school food environment and snacking behaviors in adolescents? *Health Promotion International* **2018,** *33*, 1010-1021.
7. Babashahi, M.; Omidvar, N.; Joulaei, H.; Zargaraan, A.; Zayeri, F.; Veisi, E.; Doustmohammadian, A.; Kelishadi, R. Scrutinize of healthy school canteen policy in Iran's primary schools: a mixed method study. *BMC Public Health* **2021,** *21*.
8. Kheirouri, S.; Alizadeh, M. Process evaluation of a national school-based iron supplementation program for adolescent girls in Iran. *BMC Public Health* **2014,** *14*.
9. Ministry of Health-Iraq. *National Nutrition Strategy 2012-2021*; 2012; Available online: https://extranet.who.int/nutrition/gina/en/node/8387.
10. Al-Eid, A. J.; Al-Ahmed, Z. A.; Al-Omary, S. A.; Al-Harbi, S. M. RASHAKA program: a collaborative initiative between Ministry of Health and Ministry of Education to control childhood obesity in Saudi Arabia. *Saudi Journal of Obesity* **2017,** *5*, 22-27.
11. Ministry of Health-Kingdom of Saudi Arabia. School-Based Obesity Control (Rashaqa). Available online: https://www.moh.gov.sa/en/Ministry/Projects/agility/Pages/default.aspx (accessed on 16 February 2023).
12. Behbehani, K. Kuwait national programme for healthy living: First 5-year plan (2013-2017). *Medical Principles and Practice* **2014,** *23*, 32-42.

13. Evans, C. E. L.; Albar, S. A.; Vargas-Garcia, E. J.; Xu, F. School-Based Interventions to Reduce Obesity Risk in Children in High- and Middle-Income Countries. *Advances in Food and Nutrition Research* **2015,** *76*, 29-77.
14. Habib-Mourad, C.; Hwalla, N.; Maliha, C.; Zahr, S.; Antoniades, K. Ajyal Salima a novel public–private partnership model for childhood obesity prevention in the Arab countries. *Front Public Health* **2022,** *10*.
15. Jamaluddine, Z.; Akik, C.; Safadi, G.; Abou Fakher, S.; El-Helou, N.; Moussa, S.; Anid, D.; Ghattas, H. Does a school snack make a difference? An evaluation of the World Food Programme emergency school feeding programme in Lebanon among Lebanese and Syrian refugee children. *Public Health Nutrition* **2022,** *25*, 1678-1690.
16. Hanel, T.; Miller, B.; Aboul-Enein, B.; Benajiba, N.; Kruk, J. Farm-to-school nutrition programs with special reference to Egypt and Morocco. *The North African Journal of Food and Nutrition Research* **2021,** *5*, 100-104.
17. World Food Programme. *Capacity Development and Support for the National School Feeding Programme. Standard Project Report*; 2016; Available online: https://docs.wfp.org/api/documents/39eb14d12a174f2a996ebb37ce2b56c9/download/.
18. World Health Organization. *Health-promoting schools initiative in Oman. A WHO case study in intersectoral action*; World Health Organization: Cairo, Egypt, 2013; Available online: https://applications.emro.who.int/dsaf/EMROPUB_2013_EN_1587.pdf.
19. Government of Khyber Pakhtunkhwa. *Khyber Pakhtunkhwa Multi-sectoral Integrated Nutrition Strategy*; Planning and Development Department: 2014; Available online: https://extranet.who.int/nutrition/gina/sites/default/filesstore/PAK_2014_Khyber%20Pakhtunkhwa%20Integrated%20Nutrition%20Strategy.pdf.
20. Bajraktarevic, S.; Qadi, K.; Jouda, A.; Awadallah, Y.; Abueita, R. Improving the nutritional well-being of school-age children through the nutrition-friendly schools initiative (NFSI) in the State of Palestine. *Field Exchange - Emergency Nutrition Network ENN* **2021,** 47-50.
21. World Health Organization. *Nutrition action in schools: a review of evidence related to the nutrition-friendly schools initiative*; World Health Organization: Geneva, Switzerland, 2021; Available online: https://www.who.int/publications/i/item/9789241516969.
22. Ministry of Education and Higher Education-Qatar. *Guidance for supervisors of school canteens for the academic year 2018-2019*; Health and Safety Department: 2018; Available online: https://www.edu.gov.qa/ar/Deputy/sharedservicesaffairs/healthandsafety-dept/Documents/FilesLibrary/2-GuidetoSchoolCafeteriaSupervisors.pdf.
23. Ministry of Education and Higher Education-Qatar. *Food safety and health guide*; Health and Safety Department: 2017; Available online: https://www.edu.gov.qa/ar/Deputy/sharedservicesaffairs/healthandsafety-dept/Documents/FilesLibrary/8-FoodSafetyandHealthGuide.pdf.
24. Ministry of Public Health-Qatar. *National Nutrition and Physical Activity Action Plan*; 2011; Available online: https://extranet.who.int/nutrition/gina/sites/default/filesstore/QAT%202011%20National%20Nutrition%20and%20Physical%20Activity%20Action%20Plan.pdf.
25. Ministry of Health and Prevention-UAE. *National Action Plan in Nutrition 2017-2021*; 2017; Available online: https://extranet.who.int/nutrition/gina/sites/default/filesstore/ARE%202017%20National%20Strategy%20Plan%20in%20Nutrition.pdf.
